# X-ray phase-contrast tomography for high-spatial-resolution zebrafish muscle imaging

**DOI:** 10.1038/srep16625

**Published:** 2015-11-13

**Authors:** William Vågberg, Daniel H. Larsson, Mei Li, Anders Arner, Hans M. Hertz

**Affiliations:** 1 Department of Applied Physics, KTH Royal Institute of Technology/Albanova, Stockholm, Sweden; 2 Department of Physiology and Pharmacology, Karolinska Institutet, Stockholm, Sweden

## Abstract

Imaging of muscular structure with cellular or subcellular detail in whole-body animal models is of key importance for understanding muscular disease and assessing interventions. Classical histological methods for high-resolution imaging methods require excision, fixation and staining. Here we show that the three-dimensional muscular structure of unstained whole zebrafish can be imaged with sub-5 μm detail with X-ray phase-contrast tomography. Our method relies on a laboratory propagation-based phase-contrast system tailored for detection of low-contrast 4–6 μm subcellular myofibrils. The method is demonstrated on 20 days post fertilization zebrafish larvae and comparative histology confirms that we resolve individual myofibrils in the whole-body animal. X-ray imaging of healthy zebrafish show the expected structured muscle pattern while specimen with a dystrophin deficiency (*sapje*) displays an unstructured pattern, typical of Duchenne muscular dystrophy. The method opens up for whole-body imaging with sub-cellular detail also of other types of soft tissue and in different animal models.

Phase-contrast X-ray imaging is an attractive emerging bio-imaging method when classical absorption does not provide sufficient contrast, e.g., in soft tissues^1^,^2^. Several studies have demonstrated improved discrimination between different types of soft tissue^1^ but little detail is provided of the structure within each tissue type, in particular for fully differentiated tissue in, e.g., animal models. In the present study we show that phase-contrast tomography allows three-dimensional (3D) imaging of muscles in unstained whole-body zebrafish with sub-5 μm detail, showing both overall muscle structure as well as subcellular detail (myofibrils).

The zebrafish, *Danio rerio*, is emerging as an important model for studies of the molecular genetics behind the initial development of muscle cells^3^,^4^. In addition to their general features (zebrafish reproduce easily, develop rapidly, their genome is well understood, they allow *in-vivo* studies and genetic modifications), the zebrafish muscle proteins, structure, function, and development show significant similarities with that of humans. Thus, it is a suitable animal model for studies of muscular disease such as, e.g., muscular dystrophy. Zebrafish models of muscle disease have been developed, exhibiting the morphological alterations seen in patients^5^. Such studies benefit from high-resolution imaging of the muscle structure in the whole-body animals.

Present methods for zebrafish imaging include several technologies^6^ but for imaging soft tissue such as muscles with cellular and sub-cellular detail confocal fluorescence microscopy is the standard method^7^. However, the limited penetration depth of visible light restricts whole-body studies to young zebrafish, a few days post fertilization (dpf). Furthermore, it requires fluorophore staining of the sample, and specificity and availability of zebrafish antibodies as well as penetration of the antibodies into the whole animal can be problematic. Multi-photon microscopy, second-harmonic generation imaging, and selective plane illumination microscopy have the potential to increase the penetration depth by approximately a factor two and thus operate on somewhat older fish^8^,^9^. Histology and electron microscopy provide higher detail, but do not allow whole-animal imaging since they involve sectioning and staining^6^.

X-rays provide significantly larger penetration depth and allow 3D whole-animal imaging via computed tomography (CT). When working in synchrotron absorption micro-CT, it is possible to easily obtain high spatial resolution images of skeletal features in zebrafish specimens while soft-tissue imaging typically requires staining and results in lower observable resolution^6^. Reciprocal-space imaging X-ray methods, such as small-angle-scattering (SAXS), are suitable to characterize repetitive structures such as the muscle myofibrils and have been used for functional studies of muscle contraction in 5–7 dpf larvae^10^.

There are several X-ray phase-contrast imaging techniques available: interferometry, analyzer-based imaging (ABI), grating-based imaging (GBI) and propagation-based imaging (PBI)^1^. All methods work at synchrotron light sources while GBI and PBI have also been successfully combined with laboratory sources^11^,^12^. Here we use PBI since it directly provides high spatial resolution when combined with small-spot high-brightness laboratory sources. The experimental arrangement is simple, an X-ray source, the object, and free-space propagation to the high-resolution detector^11^,^13^. PBI requires spatially coherent illumination but is fairly insensitive to the spectral width of the source. Minute refraction due to the phase shift ϕ of the X-rays within the object results in intensity variations at the detector. For short propagation distances these variations are, to a first approximation, proportional to the transverse Laplacian of the phase (∇^2^ϕ), typically visible as edge enhancement in the images. To interpret the data quantitatively, the detected image must be processed (phase retrieved) before the tomographic reconstruction^14^,^15^ (cf. Methods). Simulations for unstained zebrafish muscle fibrils show that this simple arrangement achieves orders of magnitude better observable resolution than conventional absorption-contrast imaging at equivalent exposure.

Muscles have been imaged with x-ray phase contrast previously, e.g., excised esophagus with PBI^16^, excised rat heart with GBI and PBI^17^, and several studies on insects with PBI, including sacrificed beetles^18^,^19^ and live blowfly^20^. These studies were all performed at synchrotron sources and, with the exception of the sacrificed beetles, the typical observable spatial detail ranges from several tens to a few 100:s of μm:s. The study on the dried insects^18^ shows beautiful 7–8 μm striations, possibly the sarcomeres. Laboratory studies for muscle imaging include X-ray-tube-based GBI to image chicken heart, resolving mm-sized structures^21^. Common to these studies is that little information is provided about the cellular- and sub-cellular-level structure inside homogeneous muscle tissue except for the special case of the dried insect. Higher spatial detail (10 μm and below) has indeed been demonstrated in other types of soft tissue without staining or drying but then only in heterogeneous tissue like tumors with lipid enclosures^17^ or mouse kidney with artificial contrast for microvasculature imaging^15^. Finally, we note that embryonic tissue has been imaged with cellular spatial resolution^22^.

In the present paper we show that PBI-based tomography can resolve subcellular muscle detail (individual myofibrils) as well as map the overall muscle structure in whole-body zebrafish. The imaging is performed without any staining, despite the fact that the homogeneous muscle tissue in itself provides very low natural contrast. As X-rays with energies around 10 keV have orders of magnitude greater penetration depth in tissue than visible light, the method can also be applied to older and optically opaque zebrafish. We demonstrate the method on 20 dpf larvae, both on healthy specimens and on specimen of the *sapje* mutant, which is a promising model for human Duchenne muscular dystrophy^23^. Finally, we note that the method relies on a laboratory X-ray source^24^ making it a compact system which is much more accessible than if a large facility such as a synchrotron-radiation source is employed.

## Results

### Detecting muscle myofibrils with propagation-based phase-contrast imaging (PBI)

**Figure 1:**
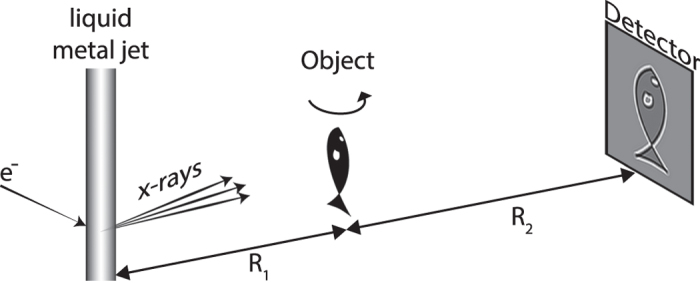
Laboratory propagation-based phase-contrast imaging of zebrafish muscle fibril structure. The small-spot liquid-metal-jet microfocus source, emitting a spectrum dominated by line emission at 9.2 keV, illuminates the 20 dpf zebrafish. The wavefront from source is perturbed by density differences in the object, producing an edge-enhanced intensity distribution at the high-resolution detector after propagating. The source-object-distance (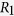) and the object-detector-distance (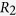) are set for maximum contrast given the source and detector properties. The object is rotated around the vertical axis for the tomography.

Figure 1 depicts the experimental arrangement. This is a classical PBI system with a liquid-metal-jet microfocus X-ray source, the zebrafish sample, and a high-resolution detector. The ability to image the muscle structure in the whole-body sample relies on the observation that the PBI signal stems from the density/composition difference between the subcellular myofibrils and their surrounding environment and not from the larger muscle cells. A prerequisite for detecting this weak and high-spatial-frequency contrast is that the imaging system must have a very small total point spread function to provide enough contrast at the spatial frequencies that need to be resolved. In our case we operate with large magnification, meaning that the source spot must be smaller than the typical myofibril diameter (i.e., here 4–6 μm) and be without tails that reduce the spatial coherence. Furthermore, the X-ray spot as well as the sample need to be kept aligned within a few microns during the full exposure time. The Methods section describes the several steps taken to achieve these necessary source and system parameters.

Also the remaining system parameters are tuned for highest myofibril contrast. The distances R_1_ and R_2_ are chosen for maximum contrast by optimizing the effective (parallel-beam equivalent) propagation distance^25^ *z*_*eff*_ = *R*_*1*_*R*_*2*_*/(R*_*1*_ + *R*_*2*_) and the object-to-detector magnification *M* = *(R*_*1*_ + *R*_*2*_*)/R*_*1*_. The contrast transfer function^25^ peaks at *z*_*eff*_ = 1/(2λ*u*^*2*^), where λ is the X-ray wavelength and *u* the spatial frequency of the smallest myofibrils. Simultaneously the magnification *M* is set to minimize the total blur induced by the finite X-ray spot size and the finite detector resolution. With our 27 μm point-spread-function detector, approximately *M* = 10 is necessary to detect few-μm details in the object. Here, the cone beam from the laboratory source is an advantage compared to a parallel synchrotron beam since it allows for a compact arrangement to produce the necessary geometric magnification. After recording the 180-degree tomographic data set, all projections are phase-retrieved with algorithms tailored for high-spatial-frequency myofibril contrast before the tomographic reconstruction. Experimental and computational details are given in the Methods section.

### Healthy 20 dpf zebrafish

**Figure 2:**
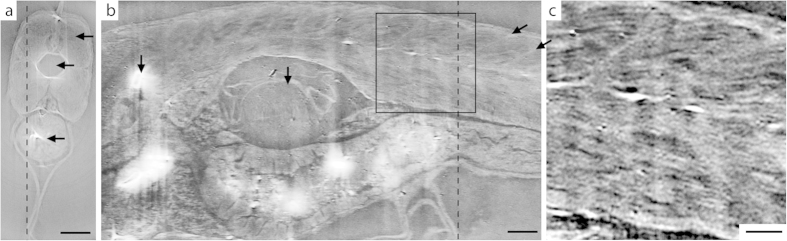
Phase-contrast tomography of 20 dpf healthy zebrafish. (**a**) Axial slice, corresponding to the dashed line through (**b**). Arrows indicate (from top) muscle tissue, notochord, and stomach. Scale bar is 100 μm. (**b**) Sagittal slice, corresponding to the dashed line through (**a**). The myofibril pattern is clearly visible. The arrows (from left) indicate bone, swim bladder and two myosepta. The scale bar is 100 μm. **(c)** Enlargement of the boxed area in (**b**). Scale bar is 50 μm.

Figure 2 shows a tomographic reconstruction of the healthy zebrafish. This 20 days post fertilization (dpf) zebrafish is 600–700 μm in diameter across the head and abdomen and 6–7 mm long. Figure 2a,b show two perpendicular slices of the whole-animal 3D data set (Supplementary Material). From Fig. 2b it is immediately obvious that propagation-based phase-contrast imaging provides a method to observe and analyze the overall structure of the muscle tissue. In addition to the thread-like pattern, shown below to be myofibrils, the myosepta are clearly visible. A myoseptum is a structure at about 45° angle from the muscle fiber long axis, separating the muscle into segments, providing attachment points for the muscle fibers and force transmission. These structures are essential for normal muscle function and exploring their role in muscle disease is of interest^26^.

Figure 2c shows an enlargement of the boxed area in Fig. 2b. The thread-like structures observed are in the 4–6 μm diameter range and the smallest observable detail is 4 μm in diameter. These numbers are significantly smaller than the typical size of a muscle cell but agree well with the size of myofibrils/myofibrillar bundles in zebrafish^27^,^28^.

### Comparison with histology

**Figure 3:**
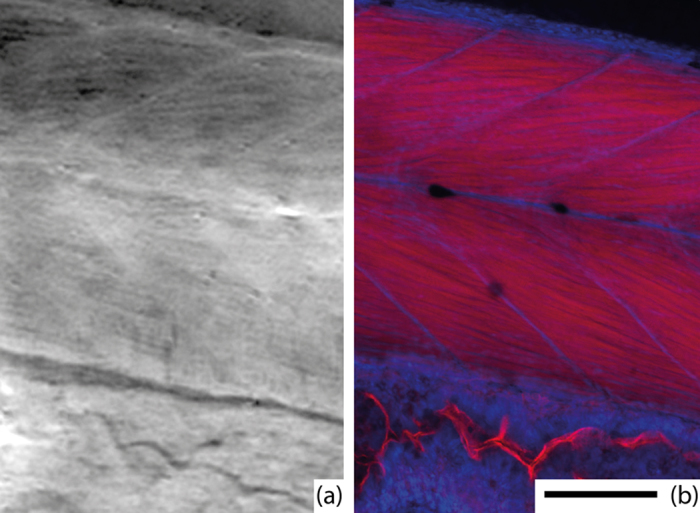
Comparison with histology. (**a**) A slice from X-ray phase-contrast tomography and **(b)** epifluorescent confocal microscopy. The images show the approximately same part of the fish. Muscle myofibrils and myosepta are clearly visible in both images. In the X-ray image, brighter corresponds to stronger phase shift, which is approximately proportional to density, whereas the confocal microscopy shows the red-stained myofibrils and blue-stained nuclei. The scale bar is 100 μm and applies to both images.

In order to verify that the thread-like structure in Fig. 2c corresponds to muscle myofibrils the same fish was doubled-stained for myofibrils (red) and nucleus (blue) and imaged by confocal microscopy. The imaging was performed at a thin sample area, towards the tail end of the fish, to allow for confocal microscopy with high resolution. Figure 3 shows the comparison of the confocal microscopy (b) and a slice from the phase-contrast tomography dataset (a). The slice is extracted from the 3D dataset and visually correlated to the confocal image to approximately cover the same area and angle as imaged by the confocal microscopy. The confocal microscopy clearly shows the 4–6 μm diameter myofibrils as well as the myosepta. The phase-contrast image shows a striking overall similarity. The myofibril contrast is better where the separation between the fibrils is larger in the confocal image.

### Muscle structure in sick and healthy zebrafish

**Figure 4:**
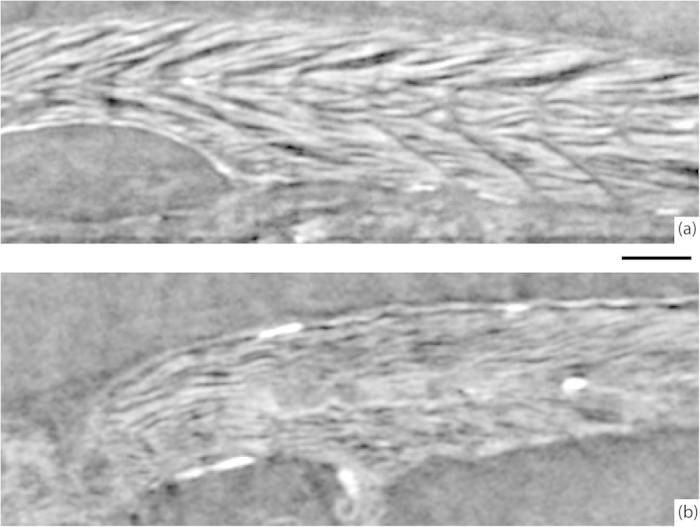
Comparison of healthy and sapje zebrafish. (**a**) Muscle structure in a 20 dpf healthy zebrafish. (**b**) Muscle structure in a 20 dpf *sapje* zebrafish. The slices are from the same anatomical location in both fish. The scale bar is 100 μm and applies to both images.

In Fig. 4, we compare sagittal slices of healthy and *sapje* zebrafish. Both fish were 20 dpf and prepared identically. The images were recorded at somewhat lower resolution than in the fish presented in Fig. 2, since the goal was to observe the overall muscle structure. Clearly, the *sapje* fish displays a significantly more disorganized muscle structure compared to the healthy control fish. The muscle myofibril pattern appear patchy and not well registered between the myosepta, in contrast to the appearance of the healthy specimen. The difference between the two fish is similar to that observed at earlier larval stages (5 dpf) where the fish body is smaller, enabling confocal imaging^23^.

## Discussion

We have demonstrated that laboratory X-ray phase-contrast tomography allows imaging of the muscular structure in whole unstained zebrafish larvae with a sufficient resolution to observe subcellular detail as myofibrils. Compared to methods based on visible light, the 10 keV X-rays allow studies on older fish due to the larger penetration depth, provides a large field of view for structural overview studies, and avoids staining. The technique has enabled us to obtain 3D structural information from muscle of whole body animal preparations at later developmental stages and observe structural defects in diseased muscle.

The method relies on the high spatial coherence of the source emission in combination with appropriate phase-retrieval algorithms. For high-spatial resolution imaging of small low-contrast structures PBI is advantageous since the magnification can be made large and the contrast transfer function^25^ enhances high spatial frequencies resulting in the phase-contrast signal being several orders of magnitude stronger than the absorption contrast signal. In the present arrangement, exposure times are long due to limited source power. However, the power-scalability of liquid-metal-jet microfocus sources^29^ shows promise for a reduction of exposure times by 1–2 orders of magnitude. Furthermore, improvements in detector technology, such as higher detective quantum efficiency^30^ at high spatial frequencies, could decrease both the dose and exposure time in future experiments.

Finally we note that methodological advances demonstrated on muscles in the present paper show promise to be applicable for imaging of other types of soft tissues, where contrast is low and high spatial resolution is desired. Possible examples include cartilage, kidney, and vascular imaging, all without staining or contrast agents. For laboratory arrangements, the key ingredients for this type of imaging is an x-ray source with a “clean”, small and stable spot in combination with high magnification and tailored phase-retrieval. Other laboratory micro- and nano-focus sources^31^,^32^,^33^ than the liquid-metal-jet should be applicable although limited flux and/or brightness may result in long exposure times. Synchrotron sources^18^ are attractive since they provide much larger flux and brightness but they naturally lack the accessibility of the laboratory sources. Inverse Compton sources^34^ have potential to develop into an important semi-laboratory tool.

## Methods

### Laboratory propagation-based phase-contrast arrangement

Figure 1 depicts the experimental arrangement with its X-ray source, sample and detector. The source is a liquid-metal-jet microfocus source (prototype from Excillum AB, Sweden) using a Galinstan alloy (Ga-In-Sn) as anode material. The emitted X-ray spectrum is dominated by the gallium K_α_ and K_β_ emission lines at 9.25 keV and 10.26 keV, respectively. The sample is placed on a rotation stage. The detector (FDI-VHR, Photonic Science, UK) has a 15 μm thick Gadox (Gd_2_O_2_S:Tb) scintillator, a 4008 × 2671 pixel CCD with a pitch of 9 μm, and a measured point spread function with a full width at half maximum (FWHM) of 27 μm.

For the high-spatial resolution imaging of the first specimen, the source was operated at 40 kVp acceleration voltage and 24 W electron-beam power in order to reduce the spot size. The X-ray emitting spot FWHM was 3 × 7 μm, as measured with a zone-plate arrangement. In order to compensate for slow thermal drifts in the source and in the 2.7 m long experimental arrangement two crossed tungsten wires (diameter 20 μm) were mounted close to the object and imaged with large magnification to the detector. This enabled accurate tracing of the effective spot movement, which was <±2μm for the full tomographic exposure. The projection images were corrected for the movement.

For the less challenging imaging the Fig. 4, the source was operated at 50 kVp/30 W, giving a spot FWHM of 5 × 9 μm, and without the crossed-wire arrangement.

### Zebrafish samples

Zebrafish of the Tubingen strain were used for imaging healthy samples. *Sapje* mutants (homozygotes for dystrophin deficiency) were used for imaging dystrophic muscles and compared with their healthy siblings. The zebrafish were kept at the Zebrafish facility at the Department of Cell and Molecular Biology, Karolinska Institutet. All methods were carried out in accordance with the European guidelines for animal research and complied with national regulations for the care of experimental animals. The protocol was approved by the Local Animal Ethical Committee for Animal Experiments in Stockholm (Permit Numbers: N193/14 and N386/11).

### Sample preparation

The zebrafish were first fixed in 4% paraformaldehyde (PFA) in phosphate buffered solution at 4 °C overnight, and then immobilized in 3% agarose gel (Sigma-Aldrich, CAS 39346-81-1) inside a plastic tube. This kept the fish stable during the exposure and the cylindrical shape of the tube is well suited for tomography. For the first specimen in Fig. 2, a polyetheretherketone (PEEK) tube (inner diameter 0.8 mm, outer diameter 1.15 mm) was used, whereas a polycarbonate tube (inner diameter 1.0 mm, outer diameter 2.0 mm) was used for two specimen in the comparison in Fig. 4.

### Data acquisition

For the high-resolution imaging in Fig. 2, the source-object-distance (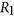) was 22.3 cm and the object-detector-distance (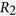) was 251.3 cm, giving the object a magnification of *M = *12.3 onto the detector. The tomography was done with 2000 projections, a step angle of 0.09°, an exposure time of 115 s per projection and a voxel size of 0.733 μm. The total exposure time was 64 hours and the total dose was calculated to 1.5 kGy. Both the healthy and the *sapje* zebrafish used in Fig. 4 were imaged using 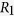 = 36 cm and 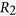 = 108 cm, giving *M = *4. The tomography was done with 1200 projections, a step angle of 0.075°, an exposure time of 94 s per projection and a voxel size of 2.25 μm. The total exposure time was 32 hours and the total dose was calculated to 750 Gy.

### Phase retrieval, data processing, and reconstruction

The flat-field corrected images were phase retrieved using the Fourier method in Rytov approximation^35^, i.e., the Fourier-space filter is


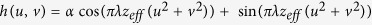


The parameters were set to optimize the visibility of individual muscle myofibrils (α = 0.022). The phase retrieval included deconvolution of the detector point spread function, which improved the resolution and contrast for small structures. To deal with the noise, which was most prominent at high spatial frequencies, the data processing also included a Wiener filter, to dampen the signal at spatial frequencies where the signal-to-noise ratio is low. From the phase-retrieved images, 3D representations of the fish were obtained by tomographic reconstruction using cone-beam-corrected filtered back projection in the Octopus software (Inside Matters, Aalst, Belgium). With the 0.73 μm voxel size the 3D dataset became 13 GB. The presented figures were gamma-corrected (γ = 1/10) and a Gaussian filter was applied to remove low-frequency variations.

### Histology

Zebrafish samples, fixed in 4% PFA from the X-ray tomography experiments, were stained with Rhodamine phalloidin (1:40, Invitrogen) for F-actin, and DRAQ5 (Biostatus, Leics, UK) for nuclei. Fluorescence confocal microscopy was performed on the whole mount preparation using a Zeiss LSM 510 microscope (Carl Zeiss, Jena, Germany).

## Additional Information

**How to cite this article**: Vågberg, W. *et al.* X-ray phase-contrast tomography for high-spatial-resolution zebrafish muscle imaging. *Sci. Rep.* **5**, 16625; doi: 10.1038/srep16625 (2015).

## Supplementary Material

Supplementary Information

Supplementary Dataset 1

Supplementary Dataset 2

Supplementary Dataset 3

Supplementary Dataset 4
