# Supplementary material for: X-ray phase-contrast tomography for high-spatial-resolution zebrafish muscle imaging
Source: Sci Rep. 2015 Nov 13;5:16625. doi: 10.1038/srep16625 (PMC4643221; doi:10.1038/srep16625)
Supplement: Supplementary Information [file srep16625-s1.pdf]

Supplementary material

X-ray phase-contrast tomography for high-spatial-resolution zebrafish muscle imaging

William Vågberg,[1] Daniel H. Larsson,[1] Mei Li,[2] Anders Arner,[2] and Hans M. Hertz[1]*

[1]Department of Applied Physics, KTH Royal Institute of Technology/Albanova, Stockholm, Sweden

[2]Department of Physiology and Pharmacology, Karolinska Institutet, Stockholm, Sweden

*Correspondence: hertz@biox.kth.se

Attached as Supplementary material is the full 3D-dataset of the high-resolution images of the zebrafish presented in Fig. 2 in the article. To comply with the data size limits of uploaded supplementary material, the 13.2 GB dataset was treated as follows: Surrounding material and some parts of the fish was cropped away, the data was binned 2x2x2 (binned voxel size 1.47 µm),and the 16-bit images were rescaled to match an 8-bit range. Although image quality certainly is reduced by the steps, the reduction was considered small by the authors. Furthermore, to comply with the 10 MB maximum upload file size, the dataset was split into four subsets. Regarding the full dataset (no cropping, 0.733 µm voxel, 16 bit, 13.2 GB), please contact the corresponding author or william.wagberg@biox.kth.se.
