# Supplementary figures and images for: X-ray phase-contrast tomography for high-spatial-resolution zebrafish muscle imaging (part 2 of 8)
Source: Sci Rep. 2015 Nov 13;5:16625. doi: 10.1038/srep16625 (PMC4643221; doi:10.1038/srep16625)

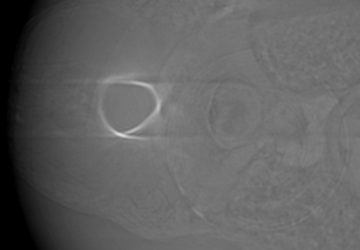

Supplement: Supplementary Dataset 1 [file srep16625-s2.zip › dataset1/0450.tif]

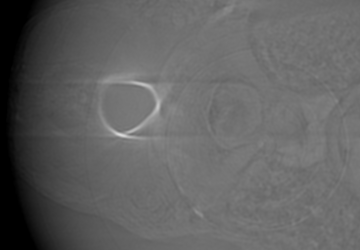

Supplement: Supplementary Dataset 1 [file srep16625-s2.zip › dataset1/0451.tif]

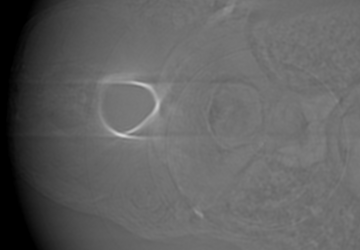

Supplement: Supplementary Dataset 1 [file srep16625-s2.zip › dataset1/0452.tif]

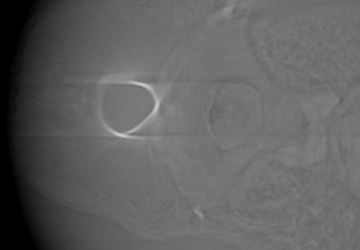

Supplement: Supplementary Dataset 1 [file srep16625-s2.zip › dataset1/0453.tif]

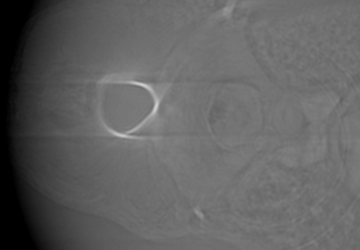

Supplement: Supplementary Dataset 1 [file srep16625-s2.zip › dataset1/0454.tif]

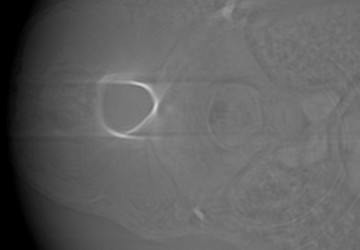

Supplement: Supplementary Dataset 1 [file srep16625-s2.zip › dataset1/0455.tif]

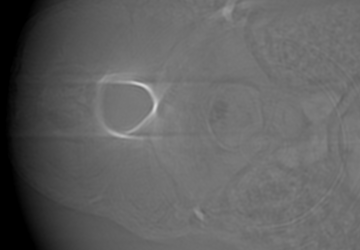

Supplement: Supplementary Dataset 1 [file srep16625-s2.zip › dataset1/0456.tif]

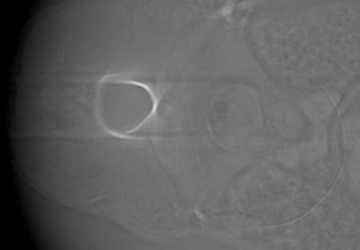

Supplement: Supplementary Dataset 1 [file srep16625-s2.zip › dataset1/0457.tif]

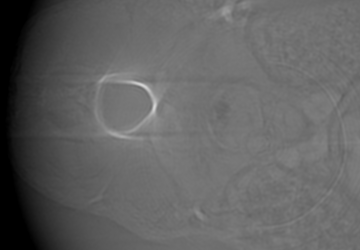

Supplement: Supplementary Dataset 1 [file srep16625-s2.zip › dataset1/0458.tif]

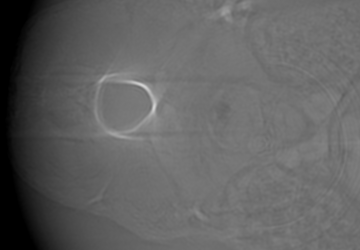

Supplement: Supplementary Dataset 1 [file srep16625-s2.zip › dataset1/0459.tif]

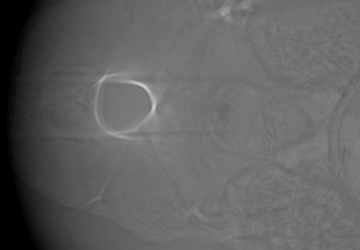

Supplement: Supplementary Dataset 1 [file srep16625-s2.zip › dataset1/0460.tif]

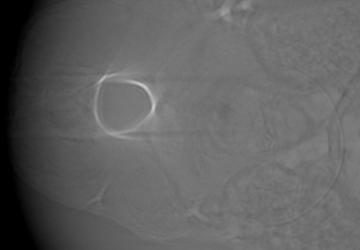

Supplement: Supplementary Dataset 1 [file srep16625-s2.zip › dataset1/0461.tif]

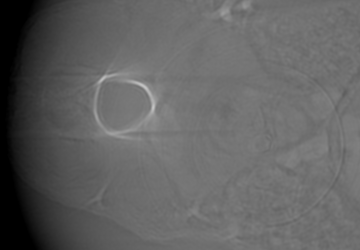

Supplement: Supplementary Dataset 1 [file srep16625-s2.zip › dataset1/0462.tif]

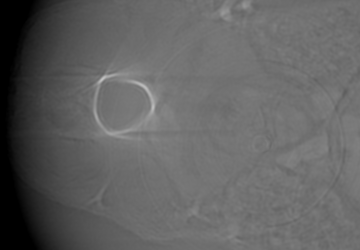

Supplement: Supplementary Dataset 1 [file srep16625-s2.zip › dataset1/0463.tif]

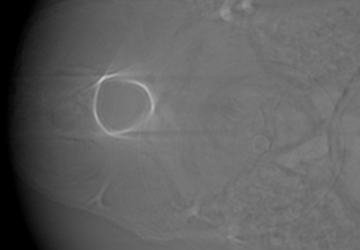

Supplement: Supplementary Dataset 1 [file srep16625-s2.zip › dataset1/0464.tif]

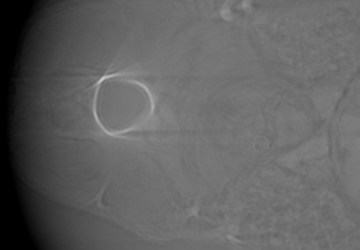

Supplement: Supplementary Dataset 1 [file srep16625-s2.zip › dataset1/0465.tif]

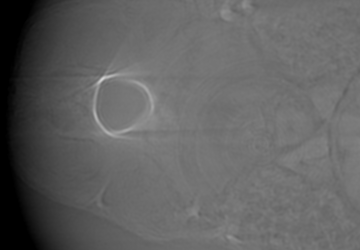

Supplement: Supplementary Dataset 1 [file srep16625-s2.zip › dataset1/0466.tif]

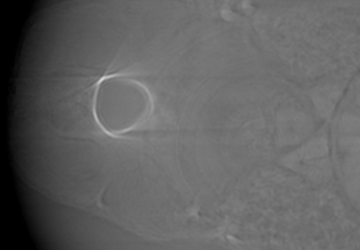

Supplement: Supplementary Dataset 1 [file srep16625-s2.zip › dataset1/0467.tif]

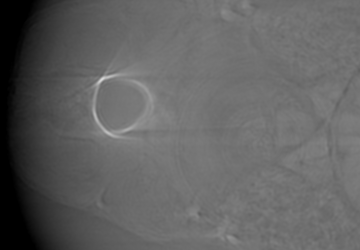

Supplement: Supplementary Dataset 1 [file srep16625-s2.zip › dataset1/0468.tif]

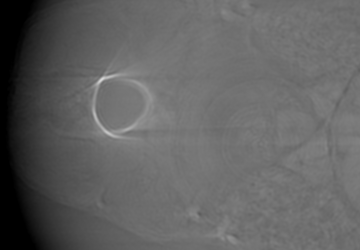

Supplement: Supplementary Dataset 1 [file srep16625-s2.zip › dataset1/0469.tif]

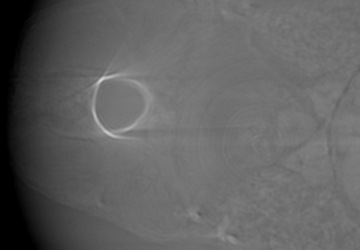

Supplement: Supplementary Dataset 1 [file srep16625-s2.zip › dataset1/0470.tif]

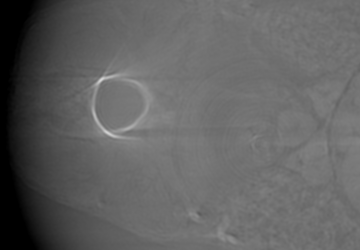

Supplement: Supplementary Dataset 1 [file srep16625-s2.zip › dataset1/0471.tif]

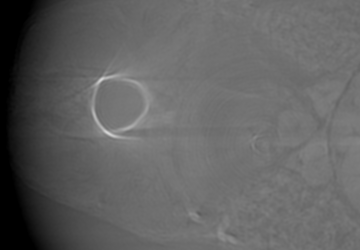

Supplement: Supplementary Dataset 1 [file srep16625-s2.zip › dataset1/0472.tif]

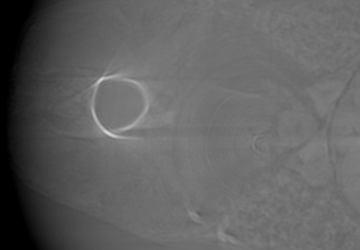

Supplement: Supplementary Dataset 1 [file srep16625-s2.zip › dataset1/0473.tif]

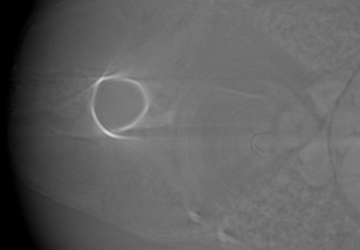

Supplement: Supplementary Dataset 1 [file srep16625-s2.zip › dataset1/0474.tif]

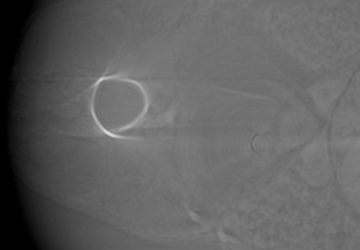

Supplement: Supplementary Dataset 1 [file srep16625-s2.zip › dataset1/0475.tif]

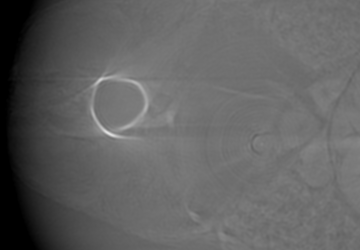

Supplement: Supplementary Dataset 1 [file srep16625-s2.zip › dataset1/0476.tif]

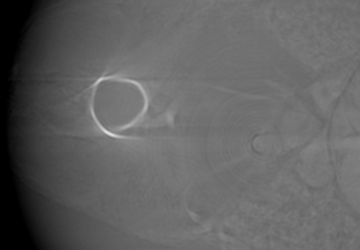

Supplement: Supplementary Dataset 1 [file srep16625-s2.zip › dataset1/0477.tif]

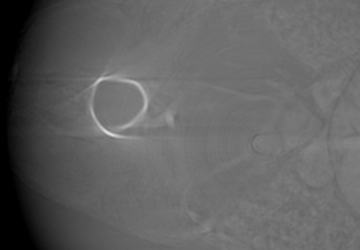

Supplement: Supplementary Dataset 1 [file srep16625-s2.zip › dataset1/0478.tif]

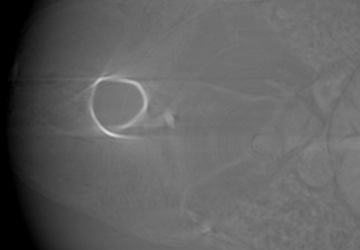

Supplement: Supplementary Dataset 1 [file srep16625-s2.zip › dataset1/0479.tif]

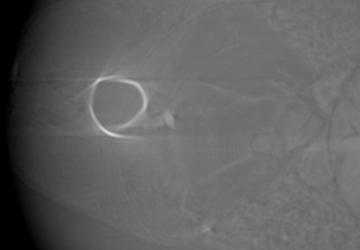

Supplement: Supplementary Dataset 1 [file srep16625-s2.zip › dataset1/0480.tif]

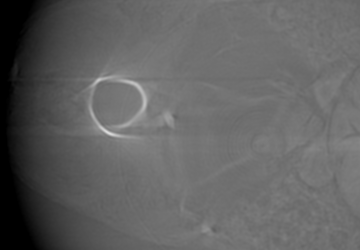

Supplement: Supplementary Dataset 1 [file srep16625-s2.zip › dataset1/0481.tif]

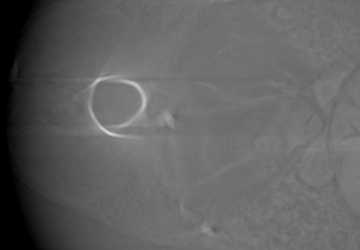

Supplement: Supplementary Dataset 1 [file srep16625-s2.zip › dataset1/0482.tif]

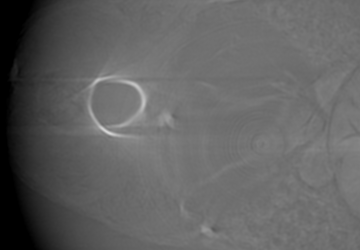

Supplement: Supplementary Dataset 1 [file srep16625-s2.zip › dataset1/0483.tif]

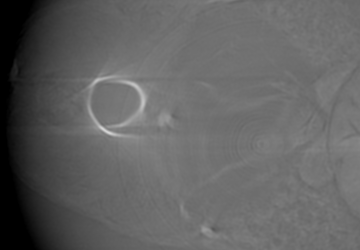

Supplement: Supplementary Dataset 1 [file srep16625-s2.zip › dataset1/0484.tif]

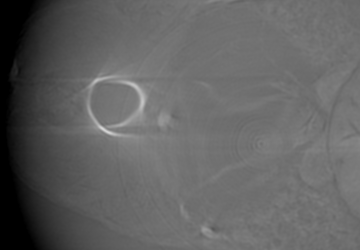

Supplement: Supplementary Dataset 1 [file srep16625-s2.zip › dataset1/0485.tif]

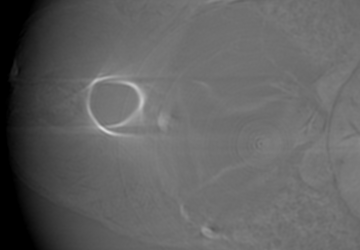

Supplement: Supplementary Dataset 1 [file srep16625-s2.zip › dataset1/0486.tif]

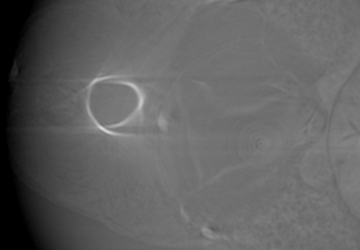

Supplement: Supplementary Dataset 1 [file srep16625-s2.zip › dataset1/0487.tif]

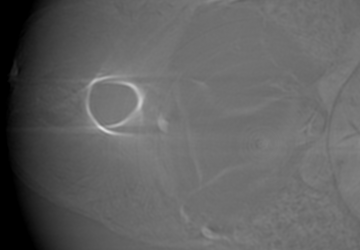

Supplement: Supplementary Dataset 1 [file srep16625-s2.zip › dataset1/0488.tif]

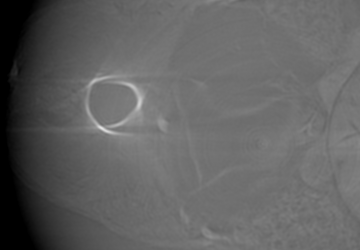

Supplement: Supplementary Dataset 1 [file srep16625-s2.zip › dataset1/0489.tif]

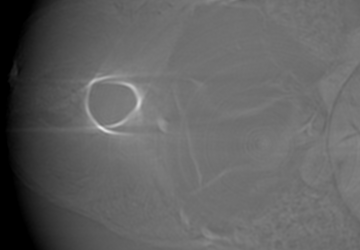

Supplement: Supplementary Dataset 1 [file srep16625-s2.zip › dataset1/0490.tif]

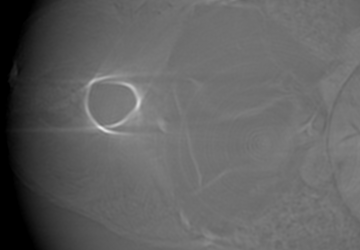

Supplement: Supplementary Dataset 1 [file srep16625-s2.zip › dataset1/0491.tif]

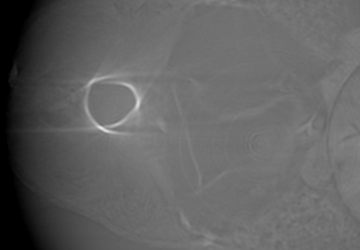

Supplement: Supplementary Dataset 1 [file srep16625-s2.zip › dataset1/0492.tif]

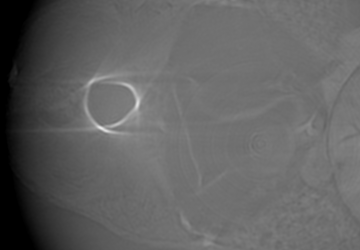

Supplement: Supplementary Dataset 1 [file srep16625-s2.zip › dataset1/0493.tif]

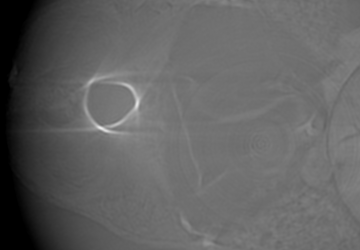

Supplement: Supplementary Dataset 1 [file srep16625-s2.zip › dataset1/0494.tif]

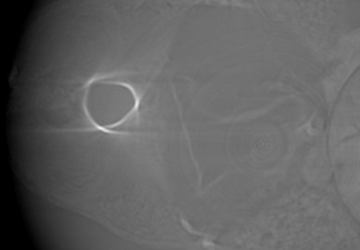

Supplement: Supplementary Dataset 1 [file srep16625-s2.zip › dataset1/0495.tif]

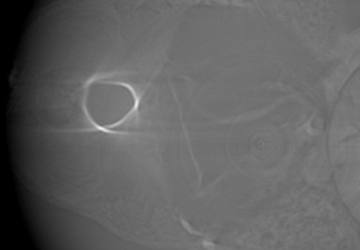

Supplement: Supplementary Dataset 1 [file srep16625-s2.zip › dataset1/0496.tif]

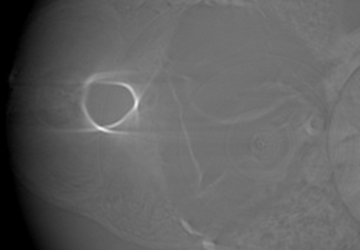

Supplement: Supplementary Dataset 1 [file srep16625-s2.zip › dataset1/0497.tif]

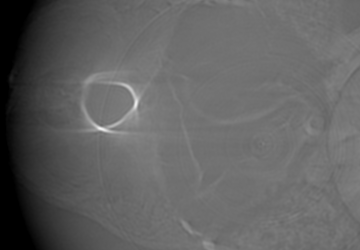

Supplement: Supplementary Dataset 1 [file srep16625-s2.zip › dataset1/0498.tif]

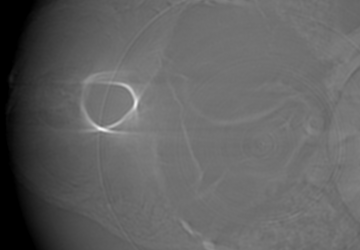

Supplement: Supplementary Dataset 1 [file srep16625-s2.zip › dataset1/0499.tif]

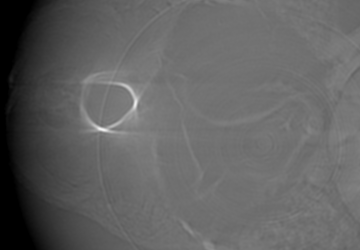

Supplement: Supplementary Dataset 1 [file srep16625-s2.zip › dataset1/0500.tif]

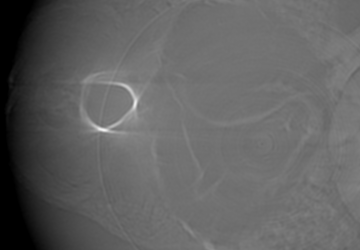

Supplement: Supplementary Dataset 1 [file srep16625-s2.zip › dataset1/0501.tif]

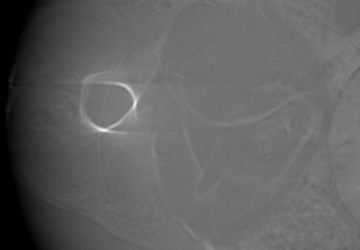

Supplement: Supplementary Dataset 1 [file srep16625-s2.zip › dataset1/0502.tif]

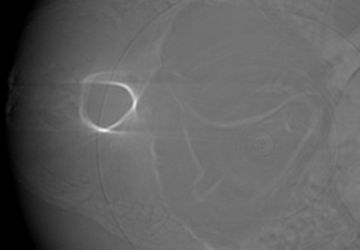

Supplement: Supplementary Dataset 1 [file srep16625-s2.zip › dataset1/0503.tif]

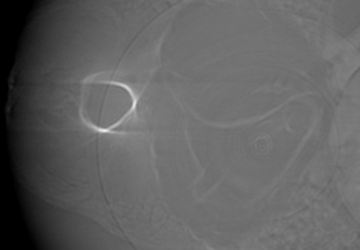

Supplement: Supplementary Dataset 1 [file srep16625-s2.zip › dataset1/0504.tif]

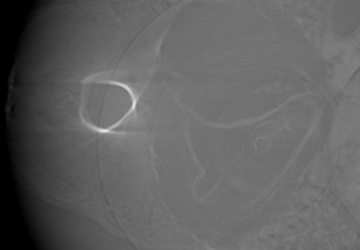

Supplement: Supplementary Dataset 1 [file srep16625-s2.zip › dataset1/0505.tif]

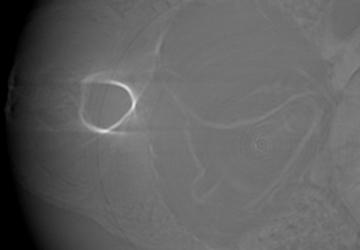

Supplement: Supplementary Dataset 1 [file srep16625-s2.zip › dataset1/0506.tif]

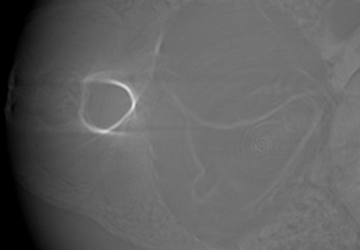

Supplement: Supplementary Dataset 1 [file srep16625-s2.zip › dataset1/0507.tif]

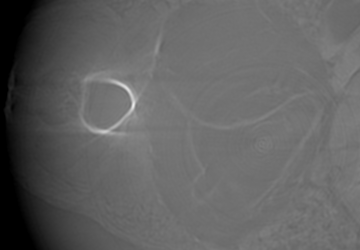

Supplement: Supplementary Dataset 1 [file srep16625-s2.zip › dataset1/0508.tif]

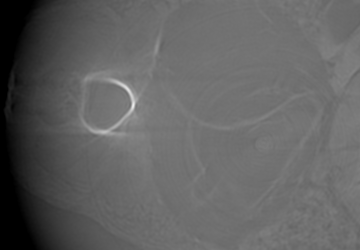

Supplement: Supplementary Dataset 1 [file srep16625-s2.zip › dataset1/0509.tif]

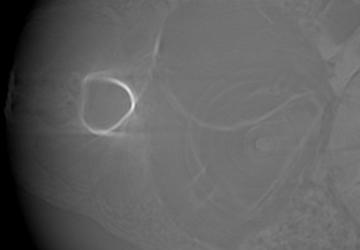

Supplement: Supplementary Dataset 1 [file srep16625-s2.zip › dataset1/0510.tif]

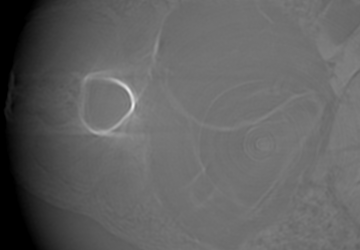

Supplement: Supplementary Dataset 1 [file srep16625-s2.zip › dataset1/0511.tif]

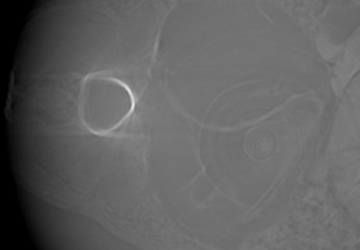

Supplement: Supplementary Dataset 1 [file srep16625-s2.zip › dataset1/0512.tif]

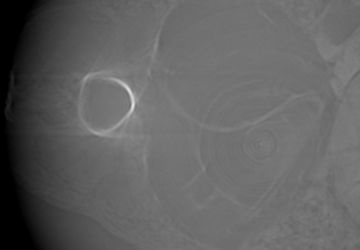

Supplement: Supplementary Dataset 1 [file srep16625-s2.zip › dataset1/0513.tif]

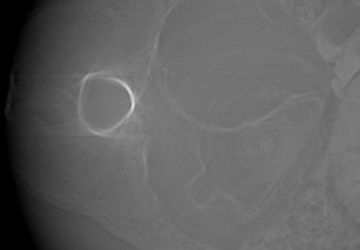

Supplement: Supplementary Dataset 1 [file srep16625-s2.zip › dataset1/0514.tif]

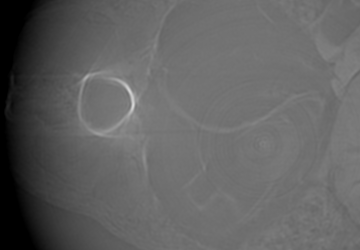

Supplement: Supplementary Dataset 1 [file srep16625-s2.zip › dataset1/0515.tif]

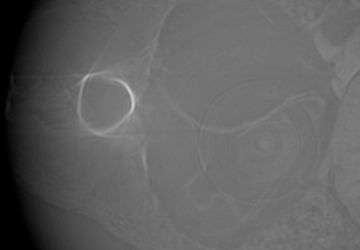

Supplement: Supplementary Dataset 1 [file srep16625-s2.zip › dataset1/0516.tif]

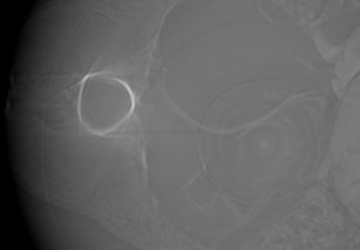

Supplement: Supplementary Dataset 1 [file srep16625-s2.zip › dataset1/0517.tif]

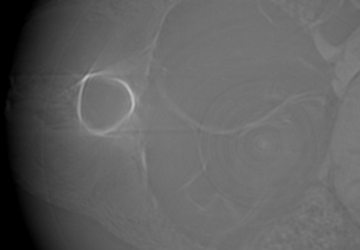

Supplement: Supplementary Dataset 1 [file srep16625-s2.zip › dataset1/0518.tif]

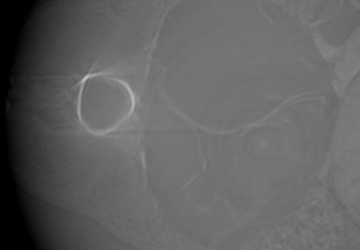

Supplement: Supplementary Dataset 1 [file srep16625-s2.zip › dataset1/0519.tif]

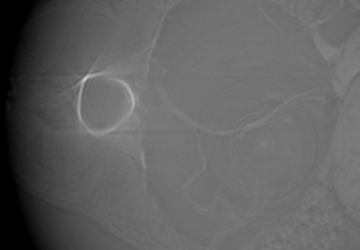

Supplement: Supplementary Dataset 1 [file srep16625-s2.zip › dataset1/0520.tif]

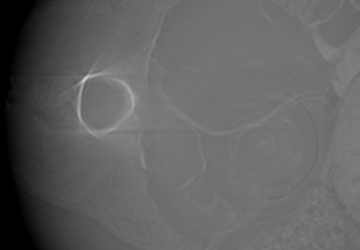

Supplement: Supplementary Dataset 1 [file srep16625-s2.zip › dataset1/0521.tif]

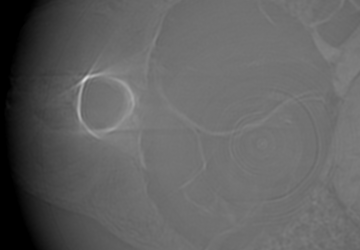

Supplement: Supplementary Dataset 1 [file srep16625-s2.zip › dataset1/0522.tif]

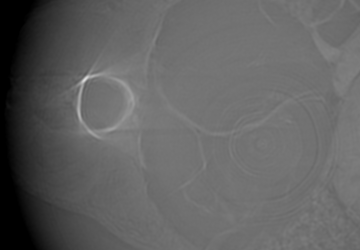

Supplement: Supplementary Dataset 1 [file srep16625-s2.zip › dataset1/0523.tif]

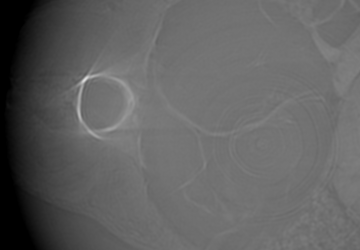

Supplement: Supplementary Dataset 1 [file srep16625-s2.zip › dataset1/0524.tif]

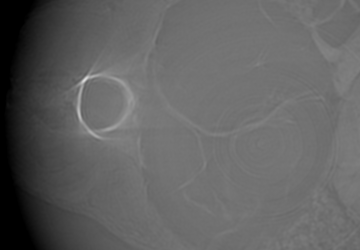

Supplement: Supplementary Dataset 1 [file srep16625-s2.zip › dataset1/0525.tif]

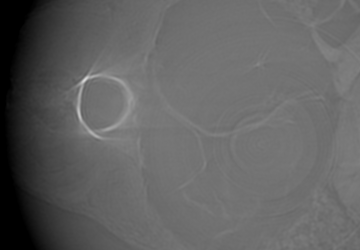

Supplement: Supplementary Dataset 1 [file srep16625-s2.zip › dataset1/0526.tif]

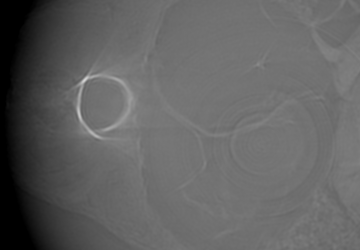

Supplement: Supplementary Dataset 1 [file srep16625-s2.zip › dataset1/0527.tif]

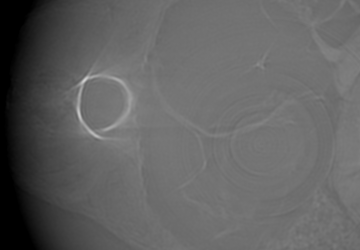

Supplement: Supplementary Dataset 1 [file srep16625-s2.zip › dataset1/0528.tif]

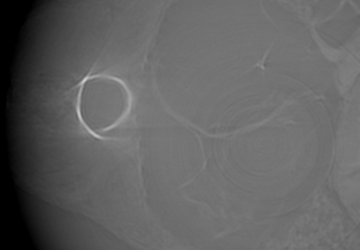

Supplement: Supplementary Dataset 1 [file srep16625-s2.zip › dataset1/0529.tif]

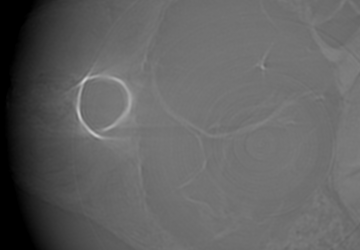

Supplement: Supplementary Dataset 1 [file srep16625-s2.zip › dataset1/0530.tif]

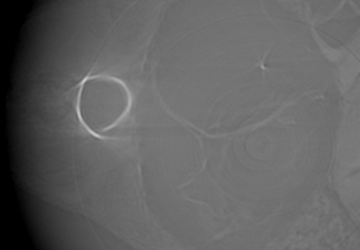

Supplement: Supplementary Dataset 1 [file srep16625-s2.zip › dataset1/0531.tif]

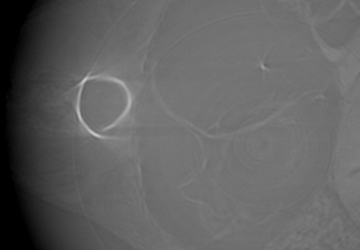

Supplement: Supplementary Dataset 1 [file srep16625-s2.zip › dataset1/0532.tif]

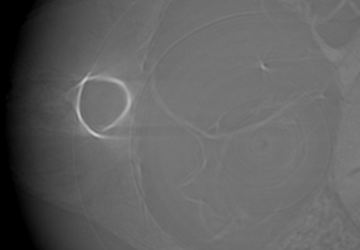

Supplement: Supplementary Dataset 1 [file srep16625-s2.zip › dataset1/0533.tif]

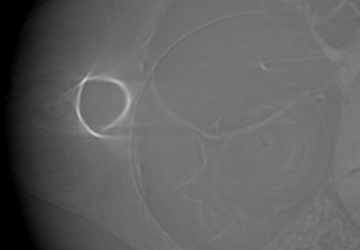

Supplement: Supplementary Dataset 1 [file srep16625-s2.zip › dataset1/0534.tif]

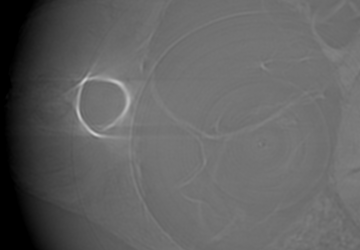

Supplement: Supplementary Dataset 1 [file srep16625-s2.zip › dataset1/0535.tif]

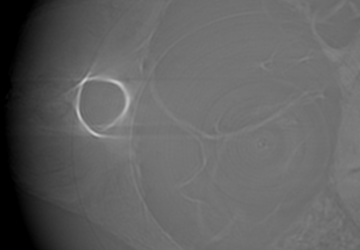

Supplement: Supplementary Dataset 1 [file srep16625-s2.zip › dataset1/0536.tif]

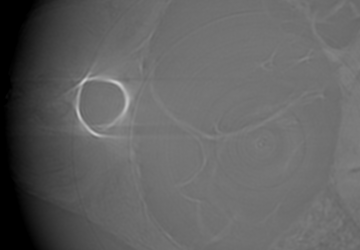

Supplement: Supplementary Dataset 1 [file srep16625-s2.zip › dataset1/0537.tif]

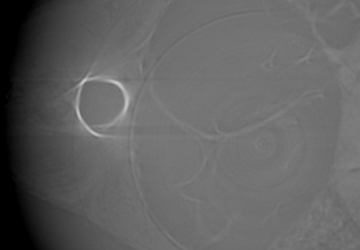

Supplement: Supplementary Dataset 1 [file srep16625-s2.zip › dataset1/0538.tif]

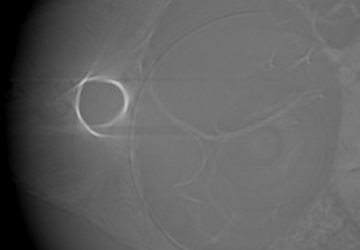

Supplement: Supplementary Dataset 1 [file srep16625-s2.zip › dataset1/0539.tif]

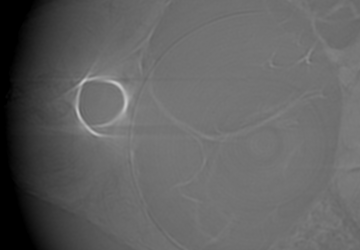

Supplement: Supplementary Dataset 1 [file srep16625-s2.zip › dataset1/0540.tif]

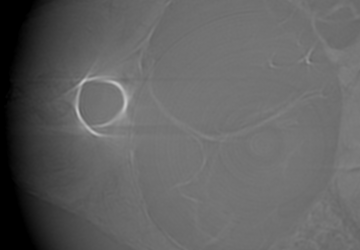

Supplement: Supplementary Dataset 1 [file srep16625-s2.zip › dataset1/0541.tif]

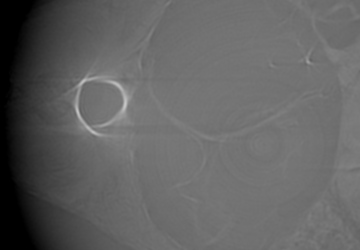

Supplement: Supplementary Dataset 1 [file srep16625-s2.zip › dataset1/0542.tif]

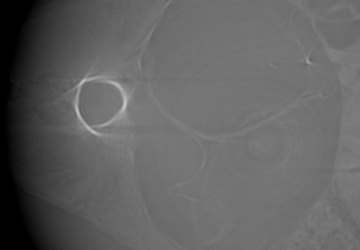

Supplement: Supplementary Dataset 1 [file srep16625-s2.zip › dataset1/0543.tif]

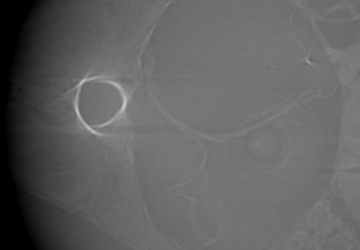

Supplement: Supplementary Dataset 1 [file srep16625-s2.zip › dataset1/0544.tif]

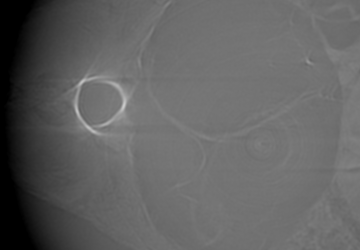

Supplement: Supplementary Dataset 1 [file srep16625-s2.zip › dataset1/0545.tif]

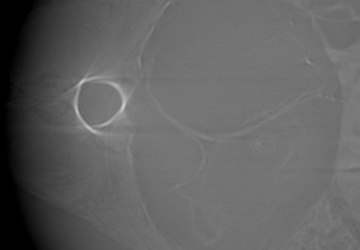

Supplement: Supplementary Dataset 1 [file srep16625-s2.zip › dataset1/0546.tif]

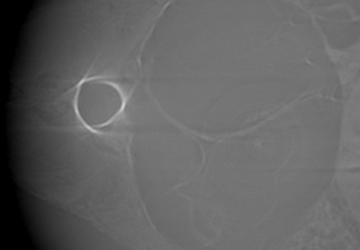

Supplement: Supplementary Dataset 1 [file srep16625-s2.zip › dataset1/0547.tif]

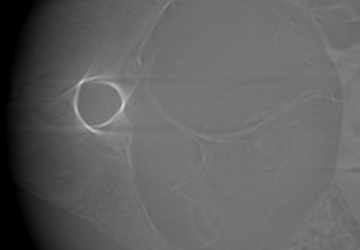

Supplement: Supplementary Dataset 1 [file srep16625-s2.zip › dataset1/0548.tif]

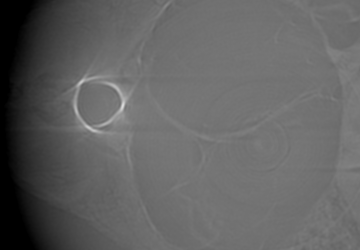

Supplement: Supplementary Dataset 1 [file srep16625-s2.zip › dataset1/0549.tif]
