# Supplementary figures and images for: X-ray phase-contrast tomography for high-spatial-resolution zebrafish muscle imaging (part 1 of 8)
Source: Sci Rep. 2015 Nov 13;5:16625. doi: 10.1038/srep16625 (PMC4643221; doi:10.1038/srep16625)

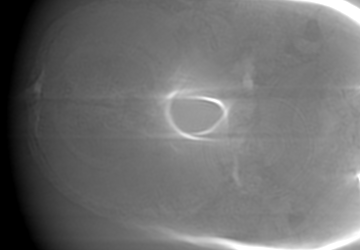

Supplement: Supplementary Dataset 1 [file srep16625-s2.zip › dataset1/0350.tif]

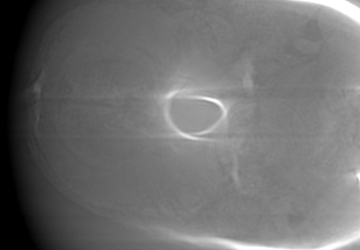

Supplement: Supplementary Dataset 1 [file srep16625-s2.zip › dataset1/0351.tif]

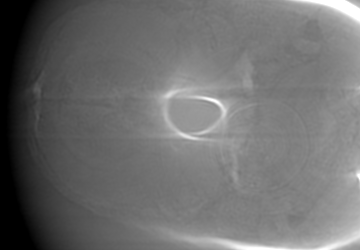

Supplement: Supplementary Dataset 1 [file srep16625-s2.zip › dataset1/0352.tif]

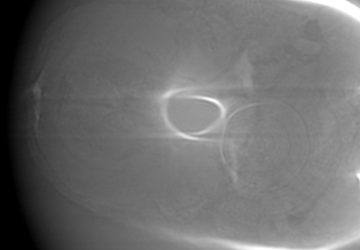

Supplement: Supplementary Dataset 1 [file srep16625-s2.zip › dataset1/0353.tif]

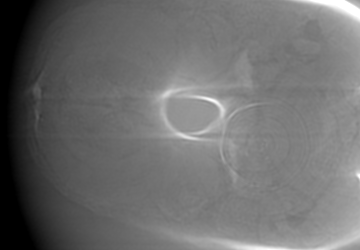

Supplement: Supplementary Dataset 1 [file srep16625-s2.zip › dataset1/0354.tif]

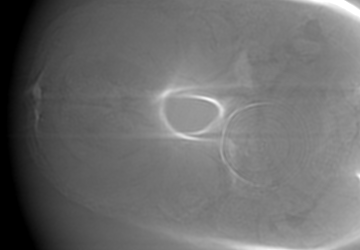

Supplement: Supplementary Dataset 1 [file srep16625-s2.zip › dataset1/0355.tif]

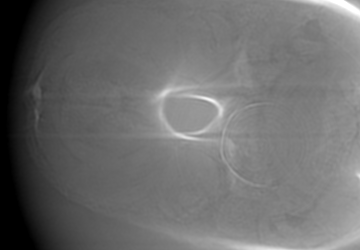

Supplement: Supplementary Dataset 1 [file srep16625-s2.zip › dataset1/0356.tif]

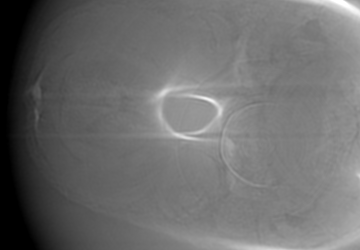

Supplement: Supplementary Dataset 1 [file srep16625-s2.zip › dataset1/0357.tif]

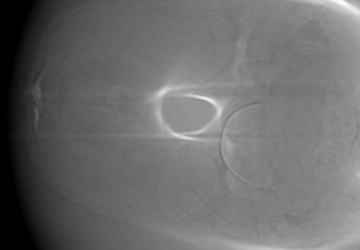

Supplement: Supplementary Dataset 1 [file srep16625-s2.zip › dataset1/0358.tif]

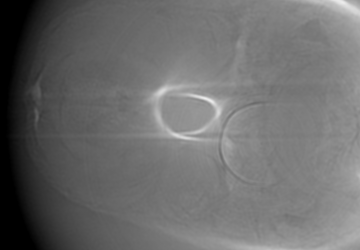

Supplement: Supplementary Dataset 1 [file srep16625-s2.zip › dataset1/0359.tif]

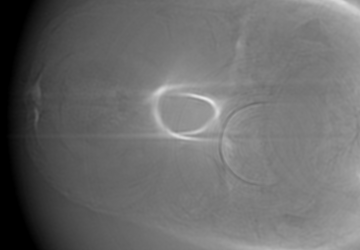

Supplement: Supplementary Dataset 1 [file srep16625-s2.zip › dataset1/0360.tif]

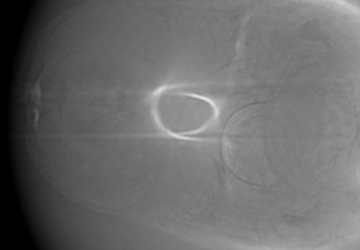

Supplement: Supplementary Dataset 1 [file srep16625-s2.zip › dataset1/0361.tif]

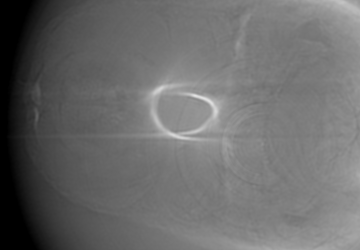

Supplement: Supplementary Dataset 1 [file srep16625-s2.zip › dataset1/0362.tif]

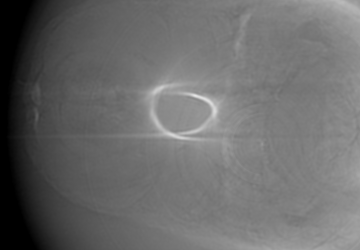

Supplement: Supplementary Dataset 1 [file srep16625-s2.zip › dataset1/0363.tif]

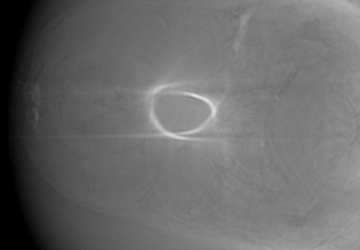

Supplement: Supplementary Dataset 1 [file srep16625-s2.zip › dataset1/0364.tif]

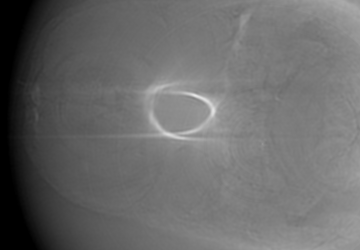

Supplement: Supplementary Dataset 1 [file srep16625-s2.zip › dataset1/0365.tif]

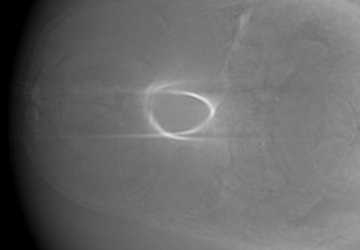

Supplement: Supplementary Dataset 1 [file srep16625-s2.zip › dataset1/0366.tif]

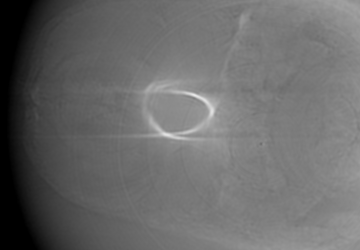

Supplement: Supplementary Dataset 1 [file srep16625-s2.zip › dataset1/0367.tif]

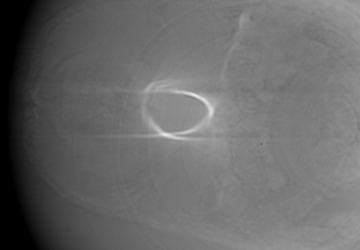

Supplement: Supplementary Dataset 1 [file srep16625-s2.zip › dataset1/0368.tif]

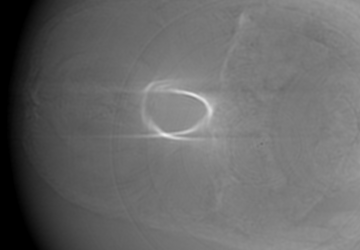

Supplement: Supplementary Dataset 1 [file srep16625-s2.zip › dataset1/0369.tif]

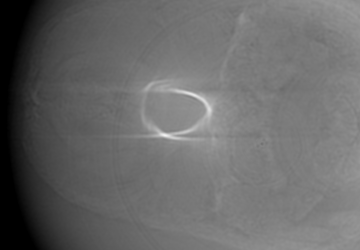

Supplement: Supplementary Dataset 1 [file srep16625-s2.zip › dataset1/0370.tif]

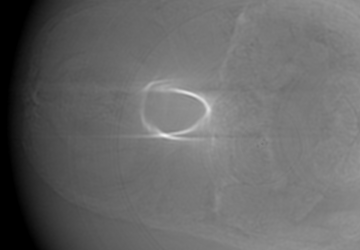

Supplement: Supplementary Dataset 1 [file srep16625-s2.zip › dataset1/0371.tif]

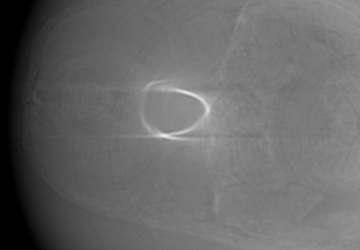

Supplement: Supplementary Dataset 1 [file srep16625-s2.zip › dataset1/0372.tif]

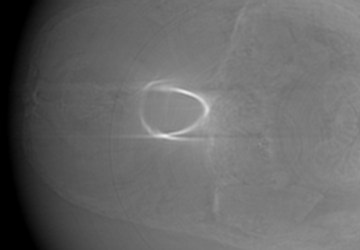

Supplement: Supplementary Dataset 1 [file srep16625-s2.zip › dataset1/0373.tif]

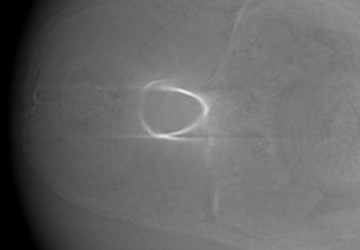

Supplement: Supplementary Dataset 1 [file srep16625-s2.zip › dataset1/0374.tif]

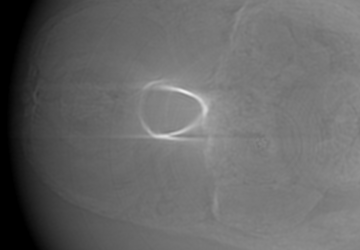

Supplement: Supplementary Dataset 1 [file srep16625-s2.zip › dataset1/0375.tif]

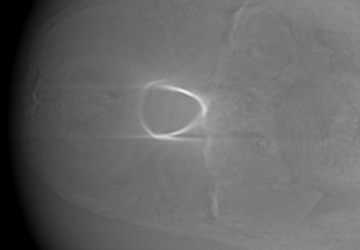

Supplement: Supplementary Dataset 1 [file srep16625-s2.zip › dataset1/0376.tif]

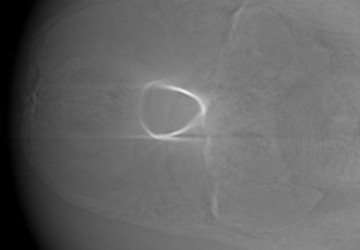

Supplement: Supplementary Dataset 1 [file srep16625-s2.zip › dataset1/0377.tif]

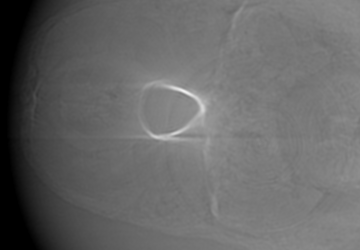

Supplement: Supplementary Dataset 1 [file srep16625-s2.zip › dataset1/0378.tif]

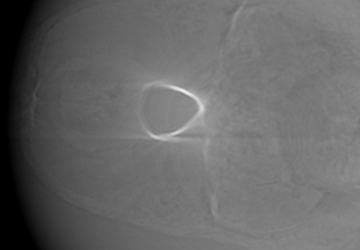

Supplement: Supplementary Dataset 1 [file srep16625-s2.zip › dataset1/0379.tif]

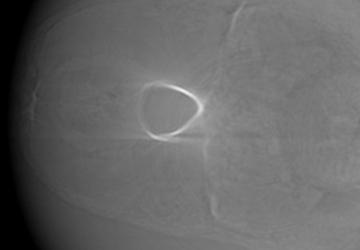

Supplement: Supplementary Dataset 1 [file srep16625-s2.zip › dataset1/0380.tif]

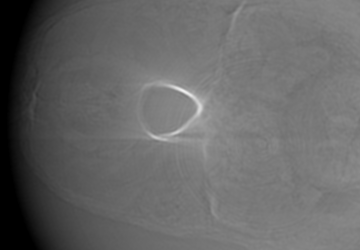

Supplement: Supplementary Dataset 1 [file srep16625-s2.zip › dataset1/0381.tif]

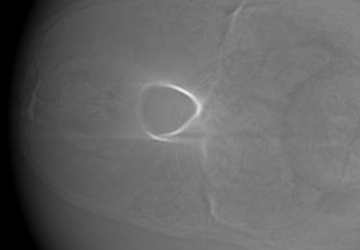

Supplement: Supplementary Dataset 1 [file srep16625-s2.zip › dataset1/0382.tif]

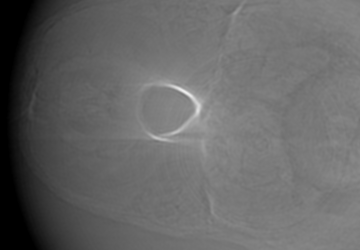

Supplement: Supplementary Dataset 1 [file srep16625-s2.zip › dataset1/0383.tif]

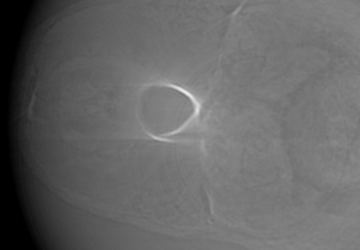

Supplement: Supplementary Dataset 1 [file srep16625-s2.zip › dataset1/0384.tif]

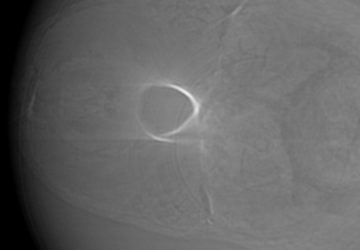

Supplement: Supplementary Dataset 1 [file srep16625-s2.zip › dataset1/0385.tif]

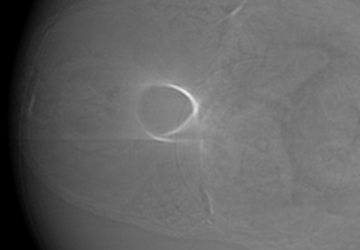

Supplement: Supplementary Dataset 1 [file srep16625-s2.zip › dataset1/0386.tif]

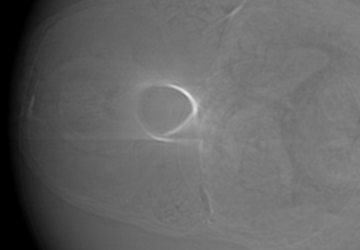

Supplement: Supplementary Dataset 1 [file srep16625-s2.zip › dataset1/0387.tif]

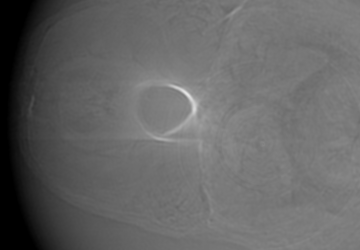

Supplement: Supplementary Dataset 1 [file srep16625-s2.zip › dataset1/0388.tif]

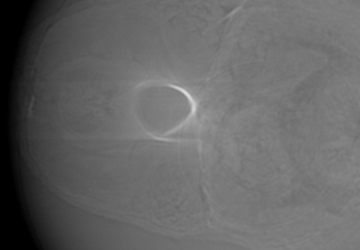

Supplement: Supplementary Dataset 1 [file srep16625-s2.zip › dataset1/0389.tif]

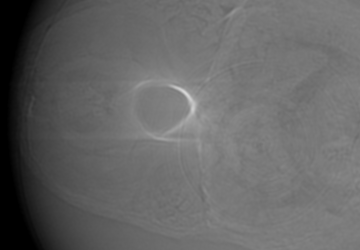

Supplement: Supplementary Dataset 1 [file srep16625-s2.zip › dataset1/0390.tif]

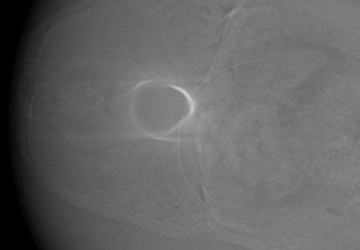

Supplement: Supplementary Dataset 1 [file srep16625-s2.zip › dataset1/0391.tif]

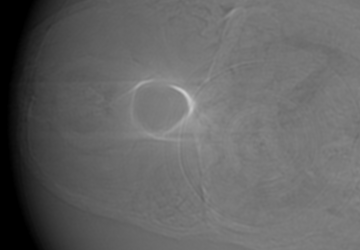

Supplement: Supplementary Dataset 1 [file srep16625-s2.zip › dataset1/0392.tif]

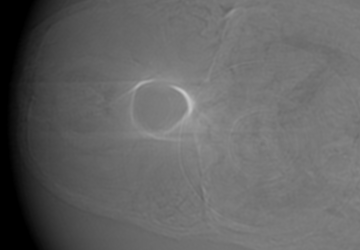

Supplement: Supplementary Dataset 1 [file srep16625-s2.zip › dataset1/0393.tif]

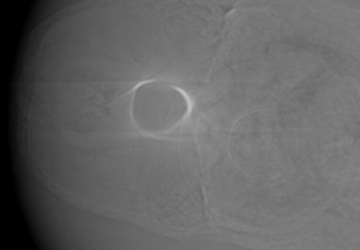

Supplement: Supplementary Dataset 1 [file srep16625-s2.zip › dataset1/0394.tif]

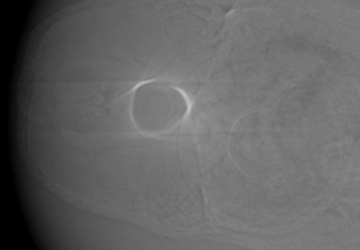

Supplement: Supplementary Dataset 1 [file srep16625-s2.zip › dataset1/0395.tif]

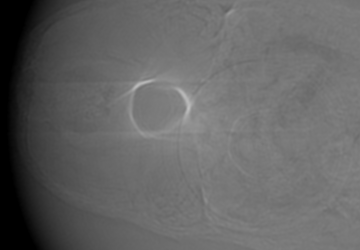

Supplement: Supplementary Dataset 1 [file srep16625-s2.zip › dataset1/0396.tif]

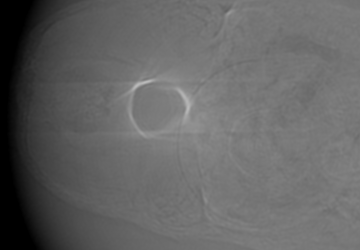

Supplement: Supplementary Dataset 1 [file srep16625-s2.zip › dataset1/0397.tif]

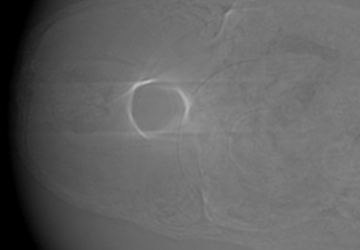

Supplement: Supplementary Dataset 1 [file srep16625-s2.zip › dataset1/0398.tif]

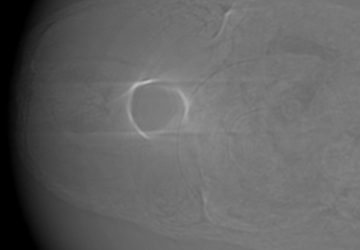

Supplement: Supplementary Dataset 1 [file srep16625-s2.zip › dataset1/0399.tif]

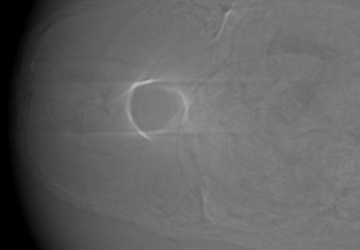

Supplement: Supplementary Dataset 1 [file srep16625-s2.zip › dataset1/0400.tif]

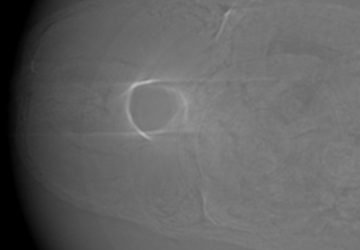

Supplement: Supplementary Dataset 1 [file srep16625-s2.zip › dataset1/0401.tif]

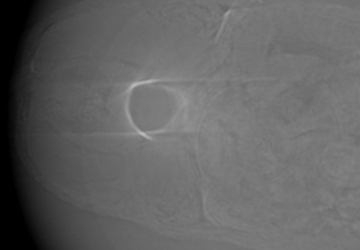

Supplement: Supplementary Dataset 1 [file srep16625-s2.zip › dataset1/0402.tif]

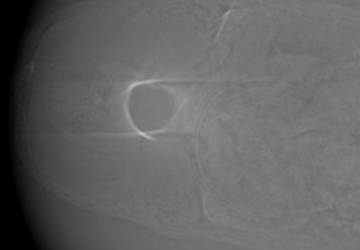

Supplement: Supplementary Dataset 1 [file srep16625-s2.zip › dataset1/0403.tif]

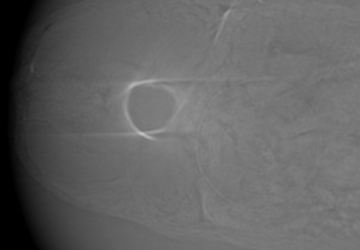

Supplement: Supplementary Dataset 1 [file srep16625-s2.zip › dataset1/0404.tif]

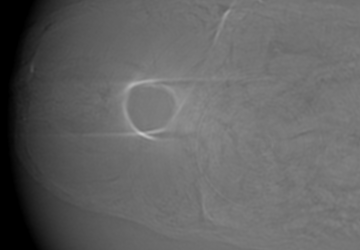

Supplement: Supplementary Dataset 1 [file srep16625-s2.zip › dataset1/0405.tif]

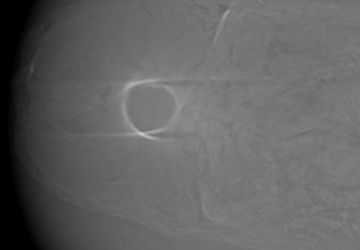

Supplement: Supplementary Dataset 1 [file srep16625-s2.zip › dataset1/0406.tif]

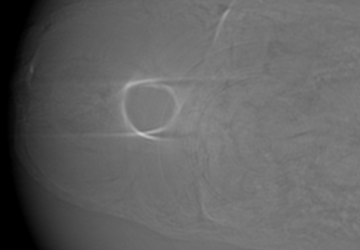

Supplement: Supplementary Dataset 1 [file srep16625-s2.zip › dataset1/0407.tif]

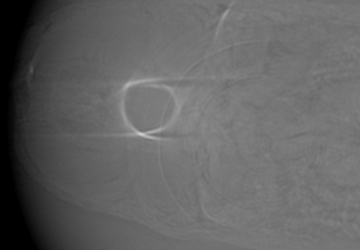

Supplement: Supplementary Dataset 1 [file srep16625-s2.zip › dataset1/0408.tif]

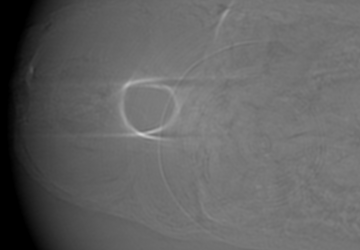

Supplement: Supplementary Dataset 1 [file srep16625-s2.zip › dataset1/0409.tif]

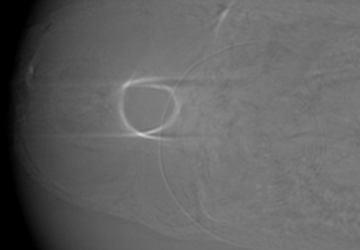

Supplement: Supplementary Dataset 1 [file srep16625-s2.zip › dataset1/0410.tif]

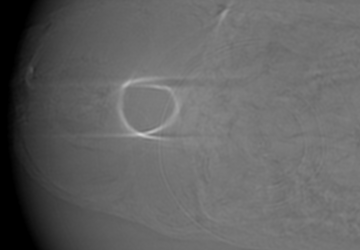

Supplement: Supplementary Dataset 1 [file srep16625-s2.zip › dataset1/0411.tif]

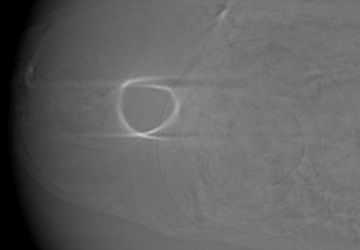

Supplement: Supplementary Dataset 1 [file srep16625-s2.zip › dataset1/0412.tif]

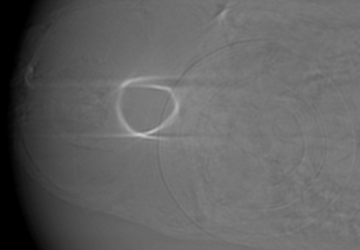

Supplement: Supplementary Dataset 1 [file srep16625-s2.zip › dataset1/0413.tif]

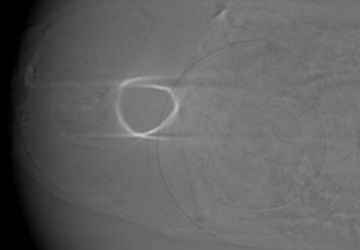

Supplement: Supplementary Dataset 1 [file srep16625-s2.zip › dataset1/0414.tif]

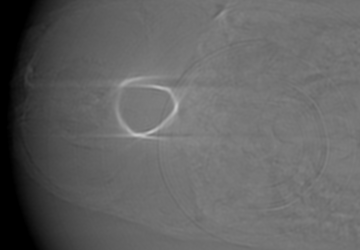

Supplement: Supplementary Dataset 1 [file srep16625-s2.zip › dataset1/0415.tif]

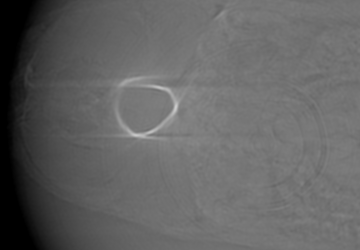

Supplement: Supplementary Dataset 1 [file srep16625-s2.zip › dataset1/0416.tif]

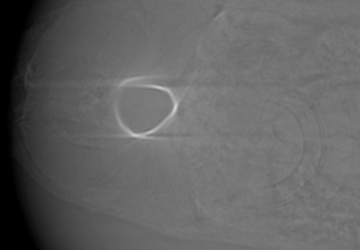

Supplement: Supplementary Dataset 1 [file srep16625-s2.zip › dataset1/0417.tif]

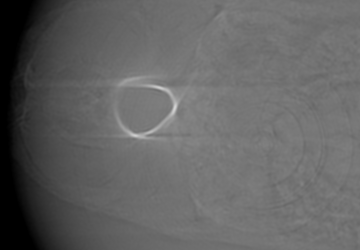

Supplement: Supplementary Dataset 1 [file srep16625-s2.zip › dataset1/0418.tif]

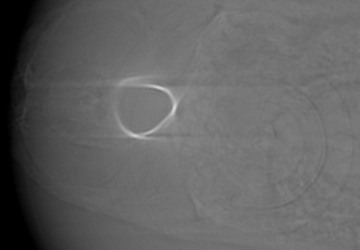

Supplement: Supplementary Dataset 1 [file srep16625-s2.zip › dataset1/0419.tif]

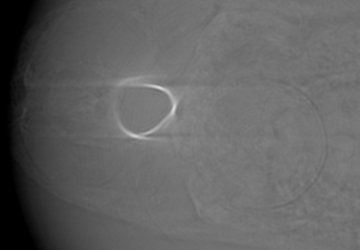

Supplement: Supplementary Dataset 1 [file srep16625-s2.zip › dataset1/0420.tif]

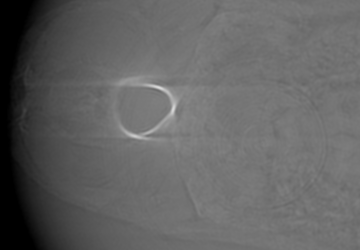

Supplement: Supplementary Dataset 1 [file srep16625-s2.zip › dataset1/0421.tif]

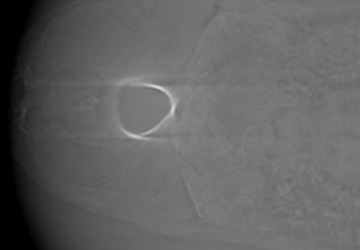

Supplement: Supplementary Dataset 1 [file srep16625-s2.zip › dataset1/0422.tif]

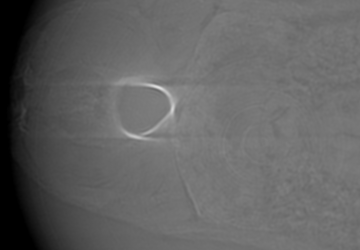

Supplement: Supplementary Dataset 1 [file srep16625-s2.zip › dataset1/0423.tif]

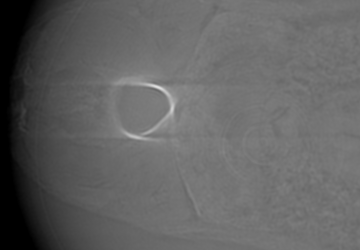

Supplement: Supplementary Dataset 1 [file srep16625-s2.zip › dataset1/0424.tif]

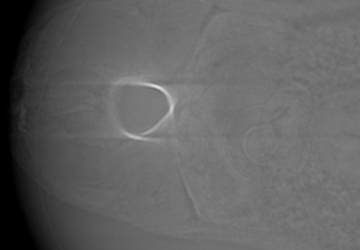

Supplement: Supplementary Dataset 1 [file srep16625-s2.zip › dataset1/0425.tif]

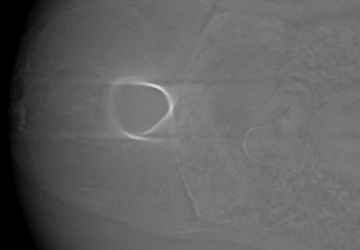

Supplement: Supplementary Dataset 1 [file srep16625-s2.zip › dataset1/0426.tif]

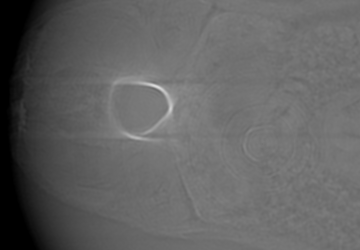

Supplement: Supplementary Dataset 1 [file srep16625-s2.zip › dataset1/0427.tif]

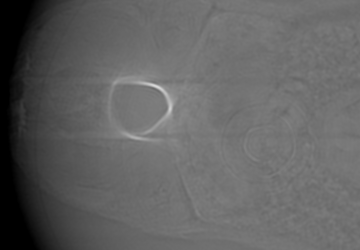

Supplement: Supplementary Dataset 1 [file srep16625-s2.zip › dataset1/0428.tif]

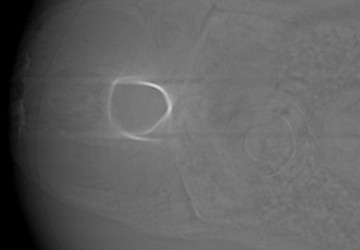

Supplement: Supplementary Dataset 1 [file srep16625-s2.zip › dataset1/0429.tif]

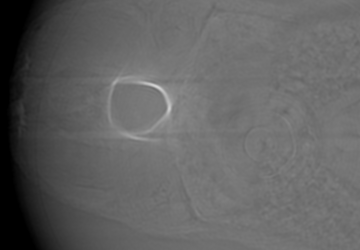

Supplement: Supplementary Dataset 1 [file srep16625-s2.zip › dataset1/0430.tif]

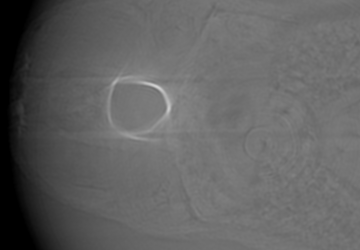

Supplement: Supplementary Dataset 1 [file srep16625-s2.zip › dataset1/0431.tif]

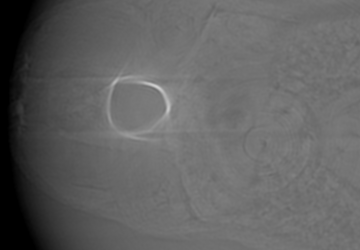

Supplement: Supplementary Dataset 1 [file srep16625-s2.zip › dataset1/0432.tif]

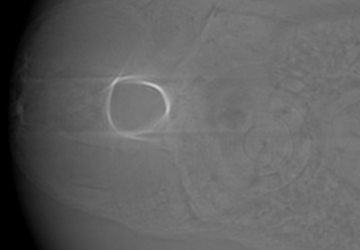

Supplement: Supplementary Dataset 1 [file srep16625-s2.zip › dataset1/0433.tif]

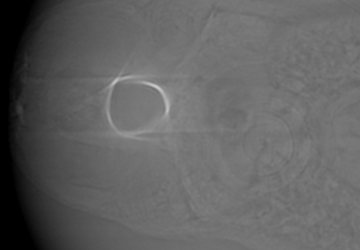

Supplement: Supplementary Dataset 1 [file srep16625-s2.zip › dataset1/0434.tif]

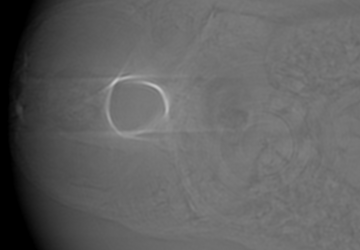

Supplement: Supplementary Dataset 1 [file srep16625-s2.zip › dataset1/0435.tif]

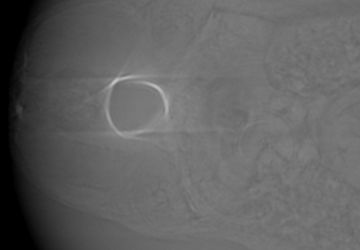

Supplement: Supplementary Dataset 1 [file srep16625-s2.zip › dataset1/0436.tif]

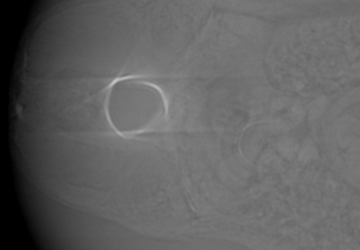

Supplement: Supplementary Dataset 1 [file srep16625-s2.zip › dataset1/0437.tif]

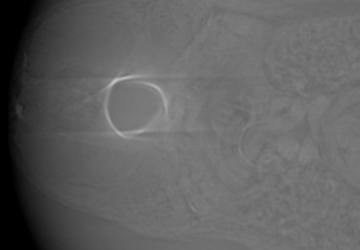

Supplement: Supplementary Dataset 1 [file srep16625-s2.zip › dataset1/0438.tif]

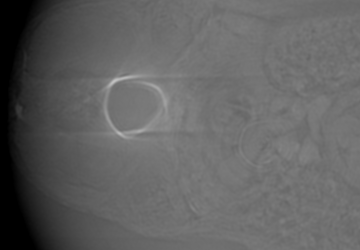

Supplement: Supplementary Dataset 1 [file srep16625-s2.zip › dataset1/0439.tif]

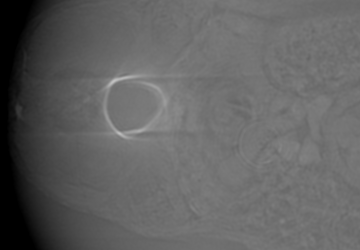

Supplement: Supplementary Dataset 1 [file srep16625-s2.zip › dataset1/0440.tif]

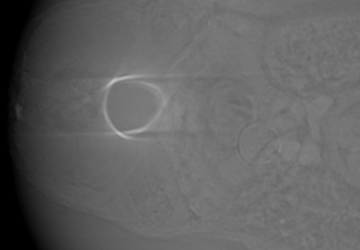

Supplement: Supplementary Dataset 1 [file srep16625-s2.zip › dataset1/0441.tif]

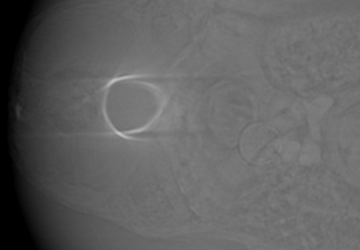

Supplement: Supplementary Dataset 1 [file srep16625-s2.zip › dataset1/0442.tif]

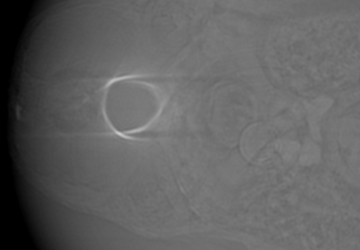

Supplement: Supplementary Dataset 1 [file srep16625-s2.zip › dataset1/0443.tif]

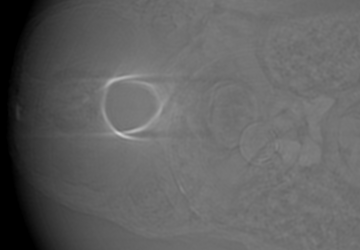

Supplement: Supplementary Dataset 1 [file srep16625-s2.zip › dataset1/0444.tif]

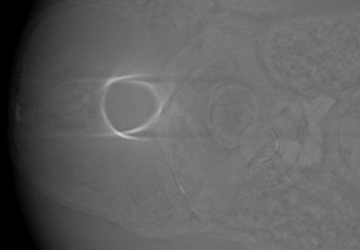

Supplement: Supplementary Dataset 1 [file srep16625-s2.zip › dataset1/0445.tif]

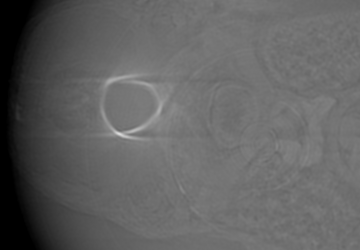

Supplement: Supplementary Dataset 1 [file srep16625-s2.zip › dataset1/0446.tif]

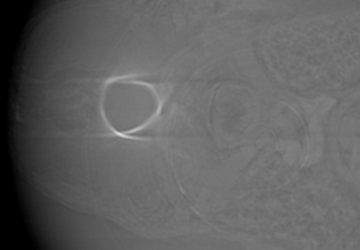

Supplement: Supplementary Dataset 1 [file srep16625-s2.zip › dataset1/0447.tif]

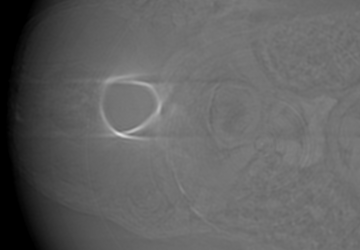

Supplement: Supplementary Dataset 1 [file srep16625-s2.zip › dataset1/0448.tif]

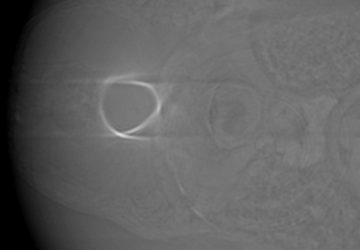

Supplement: Supplementary Dataset 1 [file srep16625-s2.zip › dataset1/0449.tif]
