# Supplementary figures and images for: X-ray phase-contrast tomography for high-spatial-resolution zebrafish muscle imaging (part 4 of 8)
Source: Sci Rep. 2015 Nov 13;5:16625. doi: 10.1038/srep16625 (PMC4643221; doi:10.1038/srep16625)

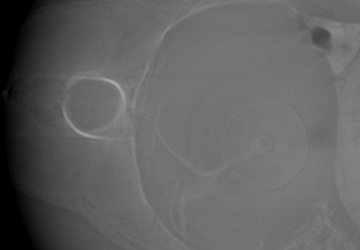

Supplement: Supplementary Dataset 2 [file srep16625-s3.zip › dataset2/0662.tif]

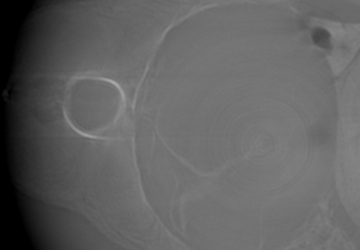

Supplement: Supplementary Dataset 2 [file srep16625-s3.zip › dataset2/0663.tif]

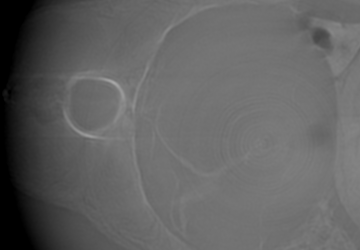

Supplement: Supplementary Dataset 2 [file srep16625-s3.zip › dataset2/0664.tif]

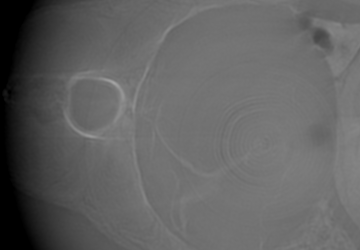

Supplement: Supplementary Dataset 2 [file srep16625-s3.zip › dataset2/0665.tif]

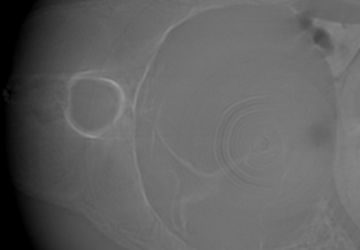

Supplement: Supplementary Dataset 2 [file srep16625-s3.zip › dataset2/0666.tif]

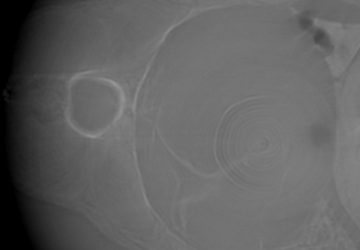

Supplement: Supplementary Dataset 2 [file srep16625-s3.zip › dataset2/0667.tif]

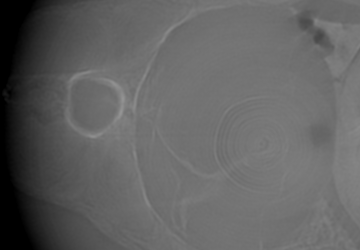

Supplement: Supplementary Dataset 2 [file srep16625-s3.zip › dataset2/0668.tif]

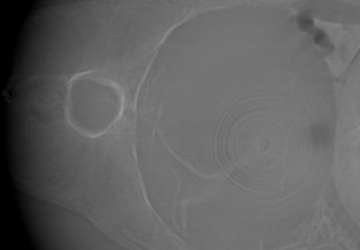

Supplement: Supplementary Dataset 2 [file srep16625-s3.zip › dataset2/0669.tif]

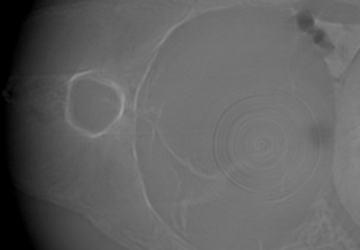

Supplement: Supplementary Dataset 2 [file srep16625-s3.zip › dataset2/0670.tif]

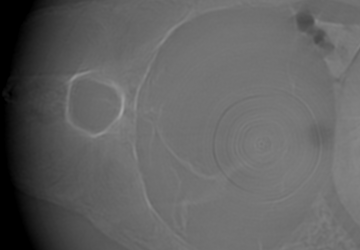

Supplement: Supplementary Dataset 2 [file srep16625-s3.zip › dataset2/0671.tif]

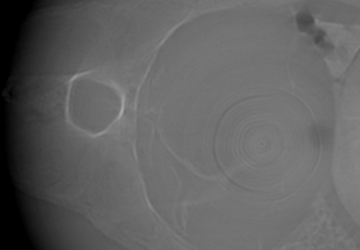

Supplement: Supplementary Dataset 2 [file srep16625-s3.zip › dataset2/0672.tif]

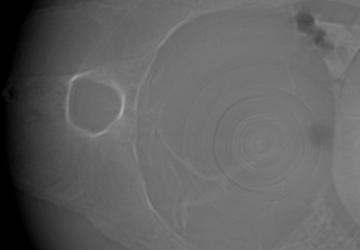

Supplement: Supplementary Dataset 2 [file srep16625-s3.zip › dataset2/0673.tif]

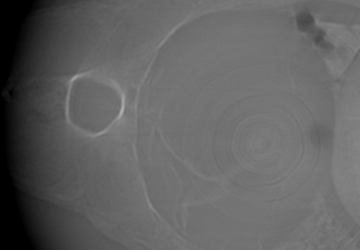

Supplement: Supplementary Dataset 2 [file srep16625-s3.zip › dataset2/0674.tif]

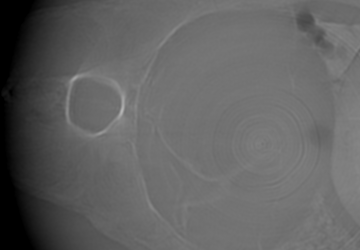

Supplement: Supplementary Dataset 2 [file srep16625-s3.zip › dataset2/0675.tif]

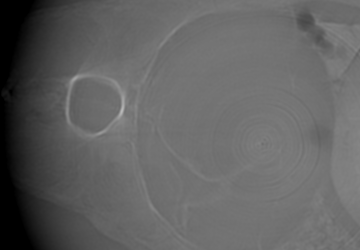

Supplement: Supplementary Dataset 2 [file srep16625-s3.zip › dataset2/0676.tif]

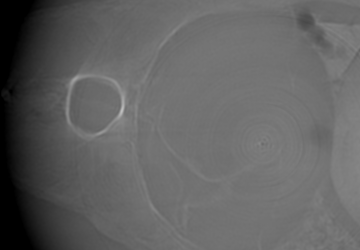

Supplement: Supplementary Dataset 2 [file srep16625-s3.zip › dataset2/0677.tif]

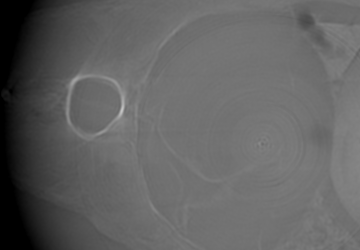

Supplement: Supplementary Dataset 2 [file srep16625-s3.zip › dataset2/0678.tif]

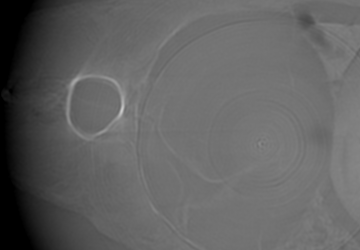

Supplement: Supplementary Dataset 2 [file srep16625-s3.zip › dataset2/0679.tif]

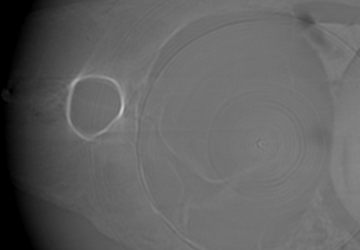

Supplement: Supplementary Dataset 2 [file srep16625-s3.zip › dataset2/0680.tif]

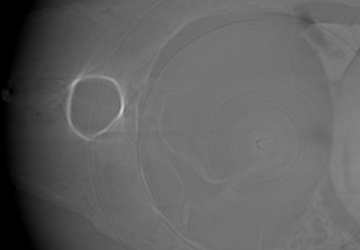

Supplement: Supplementary Dataset 2 [file srep16625-s3.zip › dataset2/0681.tif]

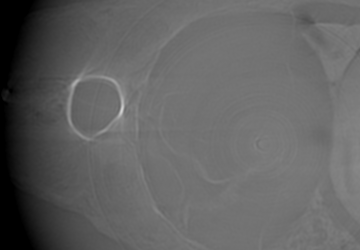

Supplement: Supplementary Dataset 2 [file srep16625-s3.zip › dataset2/0682.tif]

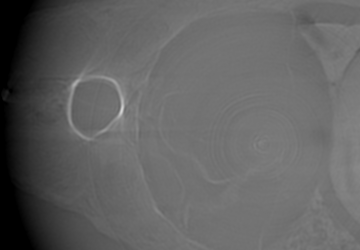

Supplement: Supplementary Dataset 2 [file srep16625-s3.zip › dataset2/0683.tif]

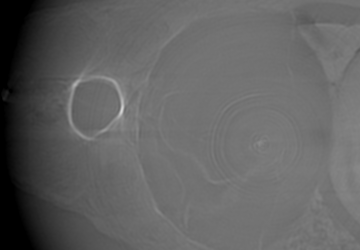

Supplement: Supplementary Dataset 2 [file srep16625-s3.zip › dataset2/0684.tif]

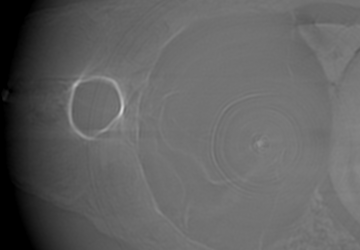

Supplement: Supplementary Dataset 2 [file srep16625-s3.zip › dataset2/0685.tif]

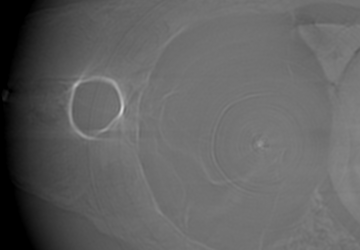

Supplement: Supplementary Dataset 2 [file srep16625-s3.zip › dataset2/0686.tif]

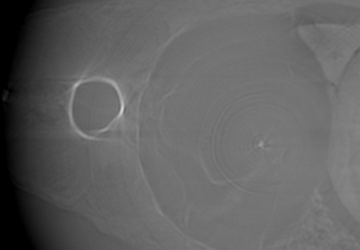

Supplement: Supplementary Dataset 2 [file srep16625-s3.zip › dataset2/0687.tif]

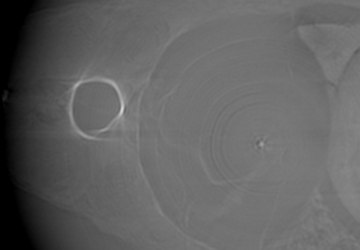

Supplement: Supplementary Dataset 2 [file srep16625-s3.zip › dataset2/0688.tif]

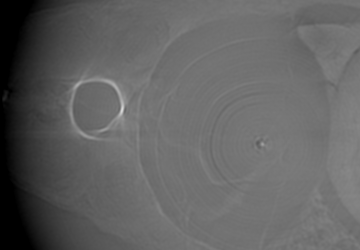

Supplement: Supplementary Dataset 2 [file srep16625-s3.zip › dataset2/0689.tif]

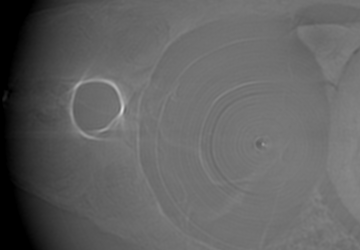

Supplement: Supplementary Dataset 2 [file srep16625-s3.zip › dataset2/0690.tif]

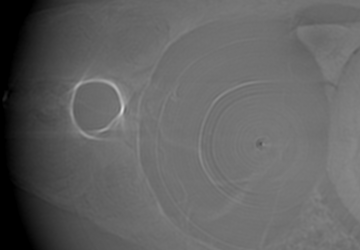

Supplement: Supplementary Dataset 2 [file srep16625-s3.zip › dataset2/0691.tif]

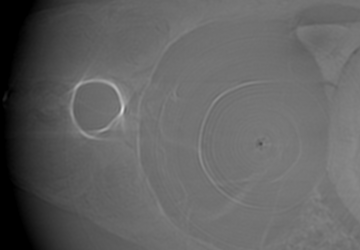

Supplement: Supplementary Dataset 2 [file srep16625-s3.zip › dataset2/0692.tif]

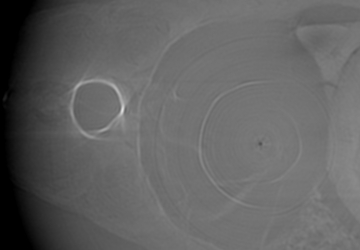

Supplement: Supplementary Dataset 2 [file srep16625-s3.zip › dataset2/0693.tif]

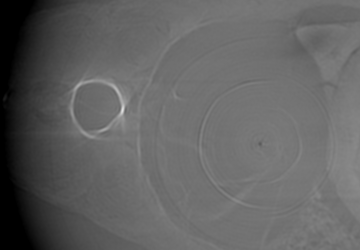

Supplement: Supplementary Dataset 2 [file srep16625-s3.zip › dataset2/0694.tif]

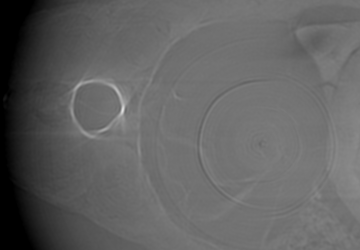

Supplement: Supplementary Dataset 2 [file srep16625-s3.zip › dataset2/0695.tif]

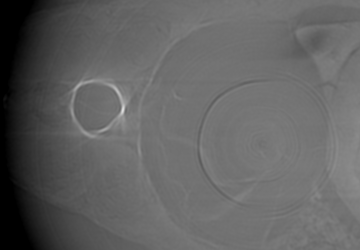

Supplement: Supplementary Dataset 2 [file srep16625-s3.zip › dataset2/0696.tif]

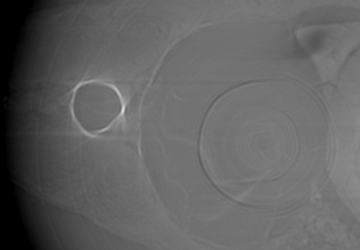

Supplement: Supplementary Dataset 2 [file srep16625-s3.zip › dataset2/0697.tif]

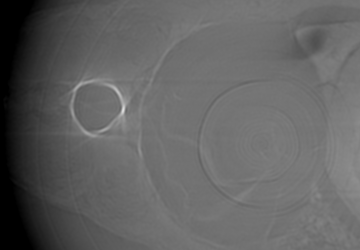

Supplement: Supplementary Dataset 2 [file srep16625-s3.zip › dataset2/0698.tif]

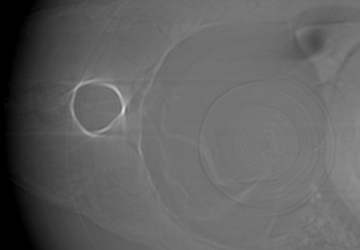

Supplement: Supplementary Dataset 2 [file srep16625-s3.zip › dataset2/0699.tif]

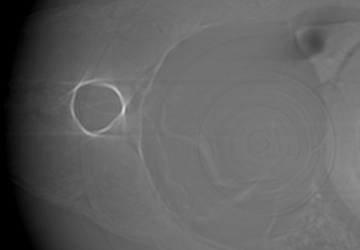

Supplement: Supplementary Dataset 2 [file srep16625-s3.zip › dataset2/0700.tif]

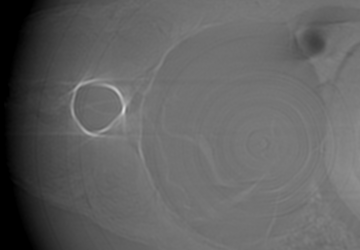

Supplement: Supplementary Dataset 2 [file srep16625-s3.zip › dataset2/0701.tif]

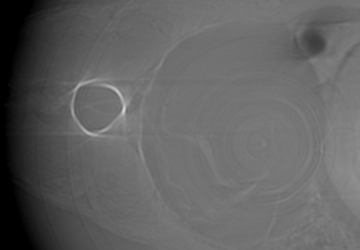

Supplement: Supplementary Dataset 2 [file srep16625-s3.zip › dataset2/0702.tif]

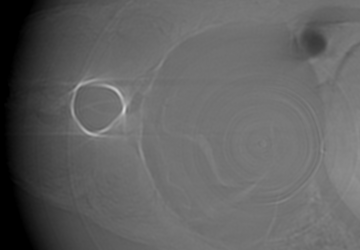

Supplement: Supplementary Dataset 2 [file srep16625-s3.zip › dataset2/0703.tif]

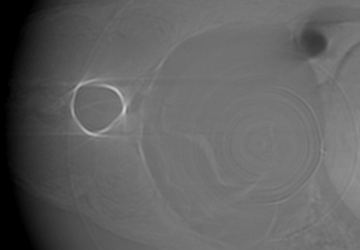

Supplement: Supplementary Dataset 2 [file srep16625-s3.zip › dataset2/0704.tif]

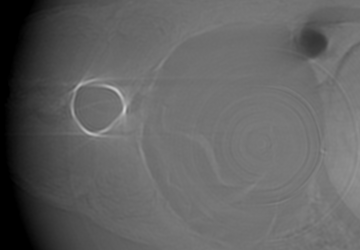

Supplement: Supplementary Dataset 2 [file srep16625-s3.zip › dataset2/0705.tif]

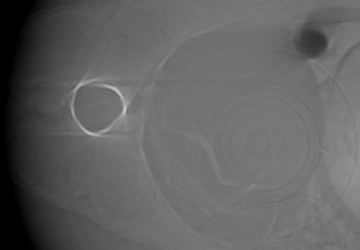

Supplement: Supplementary Dataset 2 [file srep16625-s3.zip › dataset2/0706.tif]

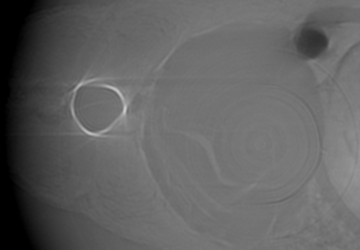

Supplement: Supplementary Dataset 2 [file srep16625-s3.zip › dataset2/0707.tif]

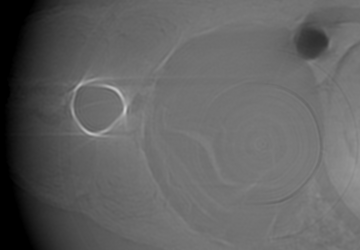

Supplement: Supplementary Dataset 2 [file srep16625-s3.zip › dataset2/0708.tif]

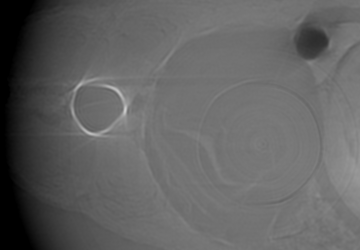

Supplement: Supplementary Dataset 2 [file srep16625-s3.zip › dataset2/0709.tif]

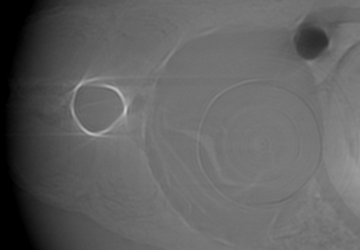

Supplement: Supplementary Dataset 2 [file srep16625-s3.zip › dataset2/0710.tif]

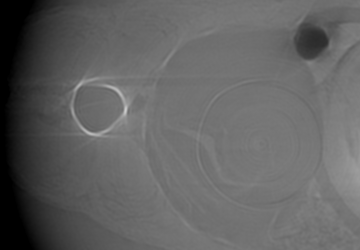

Supplement: Supplementary Dataset 2 [file srep16625-s3.zip › dataset2/0711.tif]

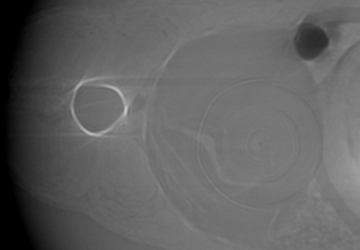

Supplement: Supplementary Dataset 2 [file srep16625-s3.zip › dataset2/0712.tif]

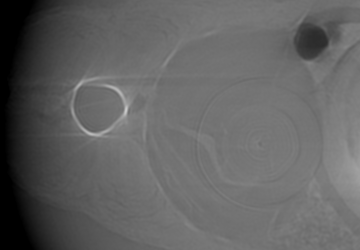

Supplement: Supplementary Dataset 2 [file srep16625-s3.zip › dataset2/0713.tif]

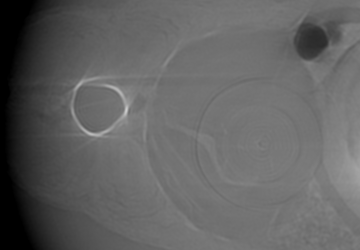

Supplement: Supplementary Dataset 2 [file srep16625-s3.zip › dataset2/0714.tif]

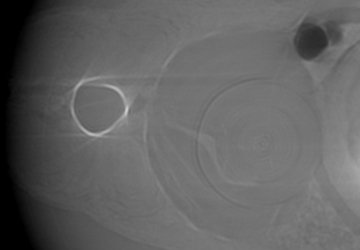

Supplement: Supplementary Dataset 2 [file srep16625-s3.zip › dataset2/0715.tif]

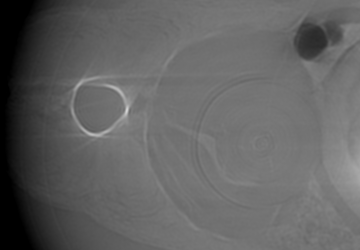

Supplement: Supplementary Dataset 2 [file srep16625-s3.zip › dataset2/0716.tif]

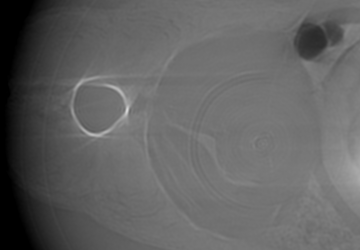

Supplement: Supplementary Dataset 2 [file srep16625-s3.zip › dataset2/0717.tif]

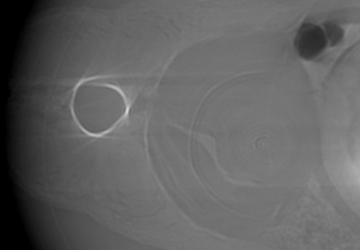

Supplement: Supplementary Dataset 2 [file srep16625-s3.zip › dataset2/0718.tif]

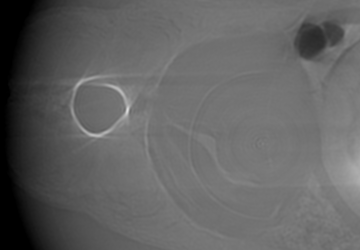

Supplement: Supplementary Dataset 2 [file srep16625-s3.zip › dataset2/0719.tif]

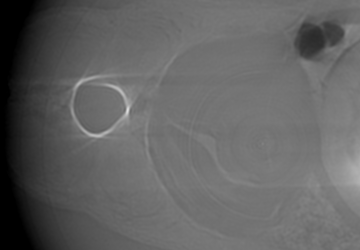

Supplement: Supplementary Dataset 2 [file srep16625-s3.zip › dataset2/0720.tif]

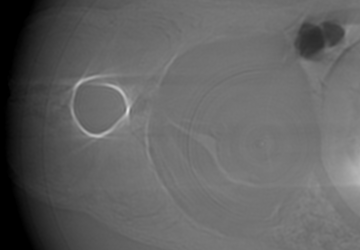

Supplement: Supplementary Dataset 2 [file srep16625-s3.zip › dataset2/0721.tif]

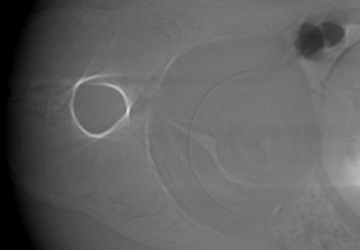

Supplement: Supplementary Dataset 2 [file srep16625-s3.zip › dataset2/0722.tif]

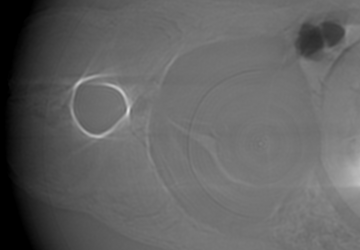

Supplement: Supplementary Dataset 2 [file srep16625-s3.zip › dataset2/0723.tif]

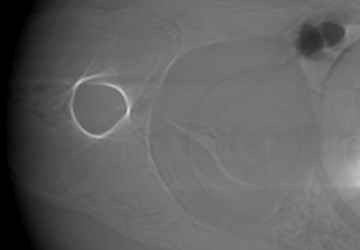

Supplement: Supplementary Dataset 2 [file srep16625-s3.zip › dataset2/0724.tif]

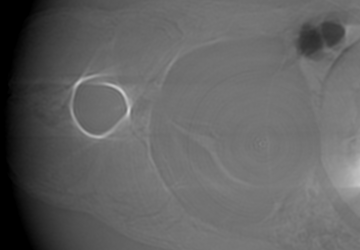

Supplement: Supplementary Dataset 2 [file srep16625-s3.zip › dataset2/0725.tif]

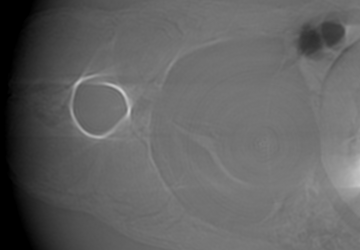

Supplement: Supplementary Dataset 2 [file srep16625-s3.zip › dataset2/0726.tif]

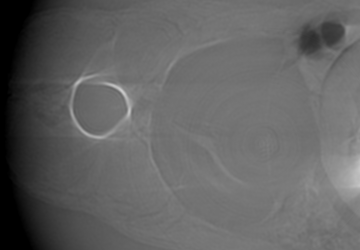

Supplement: Supplementary Dataset 2 [file srep16625-s3.zip › dataset2/0727.tif]

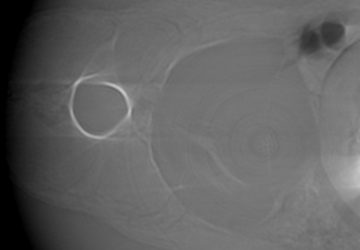

Supplement: Supplementary Dataset 2 [file srep16625-s3.zip › dataset2/0728.tif]

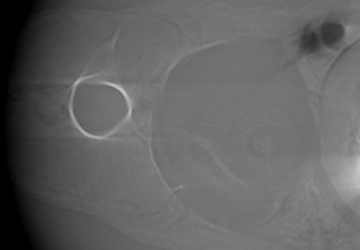

Supplement: Supplementary Dataset 2 [file srep16625-s3.zip › dataset2/0729.tif]

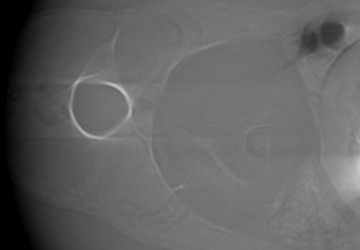

Supplement: Supplementary Dataset 2 [file srep16625-s3.zip › dataset2/0730.tif]

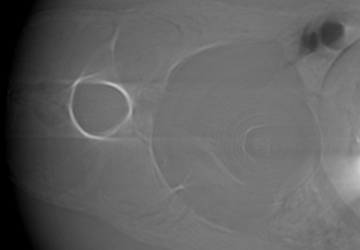

Supplement: Supplementary Dataset 2 [file srep16625-s3.zip › dataset2/0731.tif]

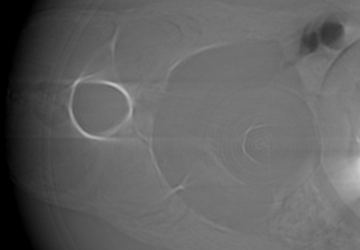

Supplement: Supplementary Dataset 2 [file srep16625-s3.zip › dataset2/0732.tif]

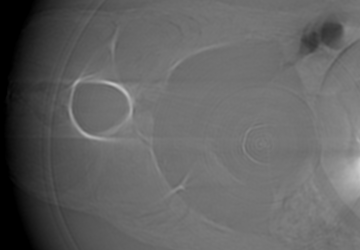

Supplement: Supplementary Dataset 2 [file srep16625-s3.zip › dataset2/0733.tif]

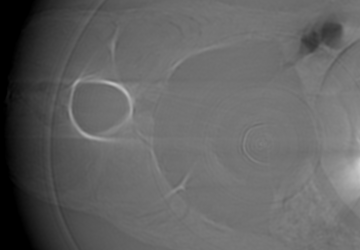

Supplement: Supplementary Dataset 2 [file srep16625-s3.zip › dataset2/0734.tif]

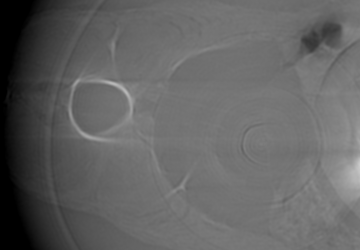

Supplement: Supplementary Dataset 2 [file srep16625-s3.zip › dataset2/0735.tif]

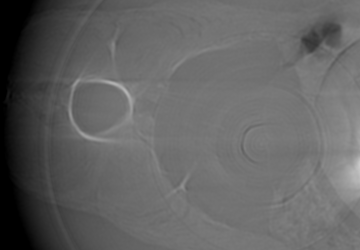

Supplement: Supplementary Dataset 2 [file srep16625-s3.zip › dataset2/0736.tif]

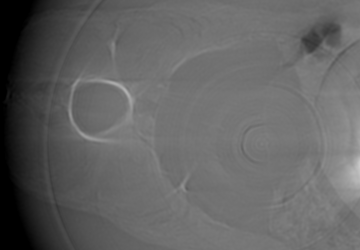

Supplement: Supplementary Dataset 2 [file srep16625-s3.zip › dataset2/0737.tif]

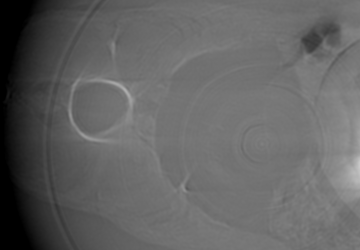

Supplement: Supplementary Dataset 2 [file srep16625-s3.zip › dataset2/0738.tif]

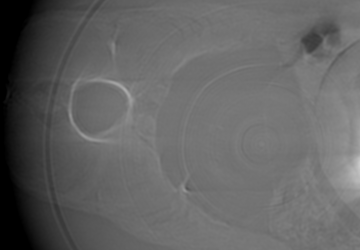

Supplement: Supplementary Dataset 2 [file srep16625-s3.zip › dataset2/0739.tif]

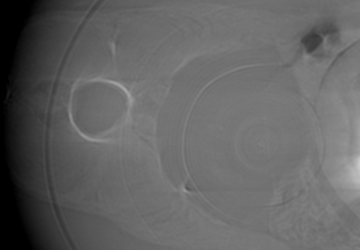

Supplement: Supplementary Dataset 2 [file srep16625-s3.zip › dataset2/0740.tif]

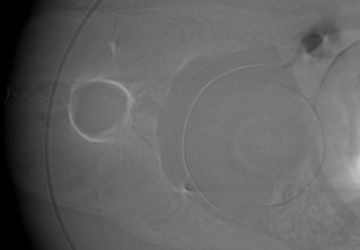

Supplement: Supplementary Dataset 2 [file srep16625-s3.zip › dataset2/0741.tif]

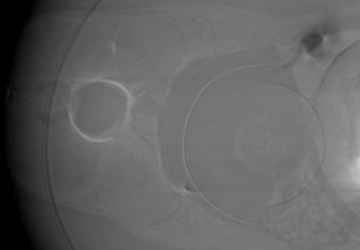

Supplement: Supplementary Dataset 2 [file srep16625-s3.zip › dataset2/0742.tif]

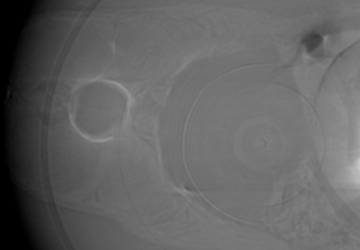

Supplement: Supplementary Dataset 2 [file srep16625-s3.zip › dataset2/0743.tif]

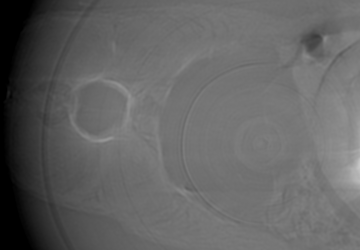

Supplement: Supplementary Dataset 2 [file srep16625-s3.zip › dataset2/0744.tif]

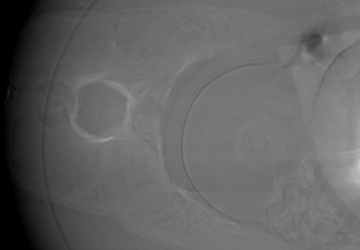

Supplement: Supplementary Dataset 2 [file srep16625-s3.zip › dataset2/0745.tif]

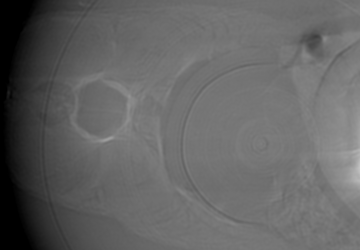

Supplement: Supplementary Dataset 2 [file srep16625-s3.zip › dataset2/0746.tif]

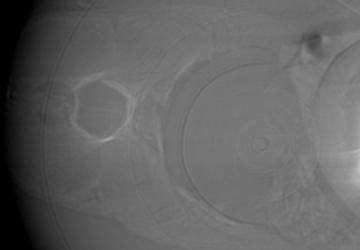

Supplement: Supplementary Dataset 2 [file srep16625-s3.zip › dataset2/0747.tif]

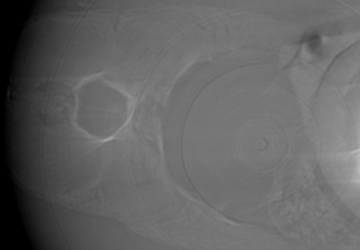

Supplement: Supplementary Dataset 2 [file srep16625-s3.zip › dataset2/0748.tif]

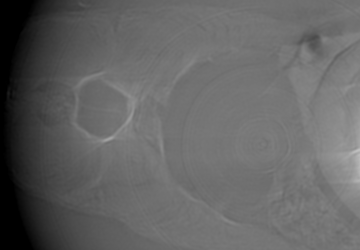

Supplement: Supplementary Dataset 2 [file srep16625-s3.zip › dataset2/0749.tif]

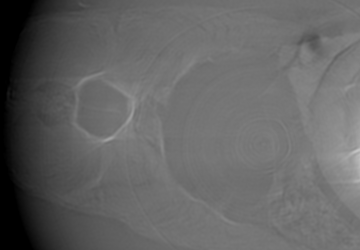

Supplement: Supplementary Dataset 2 [file srep16625-s3.zip › dataset2/0750.tif]

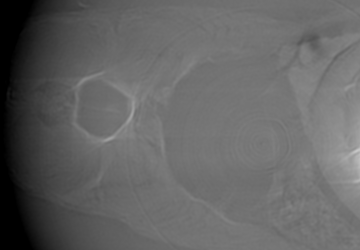

Supplement: Supplementary Dataset 2 [file srep16625-s3.zip › dataset2/0751.tif]

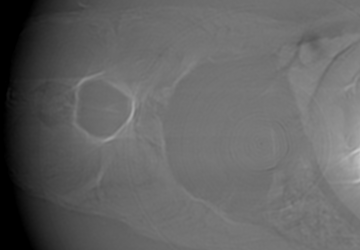

Supplement: Supplementary Dataset 2 [file srep16625-s3.zip › dataset2/0752.tif]

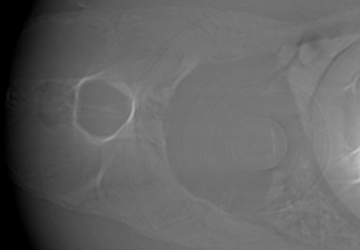

Supplement: Supplementary Dataset 2 [file srep16625-s3.zip › dataset2/0753.tif]

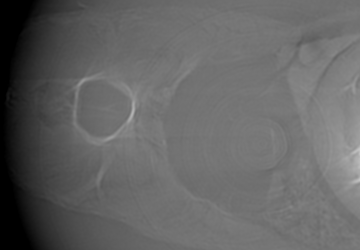

Supplement: Supplementary Dataset 2 [file srep16625-s3.zip › dataset2/0754.tif]

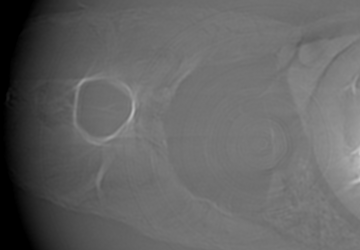

Supplement: Supplementary Dataset 2 [file srep16625-s3.zip › dataset2/0755.tif]

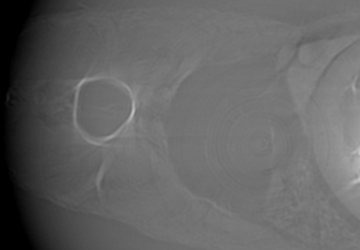

Supplement: Supplementary Dataset 2 [file srep16625-s3.zip › dataset2/0756.tif]

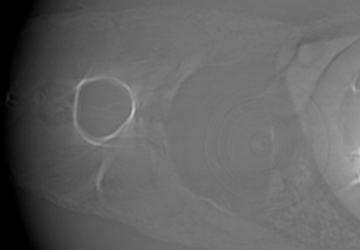

Supplement: Supplementary Dataset 2 [file srep16625-s3.zip › dataset2/0757.tif]

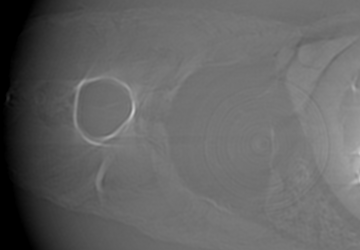

Supplement: Supplementary Dataset 2 [file srep16625-s3.zip › dataset2/0758.tif]

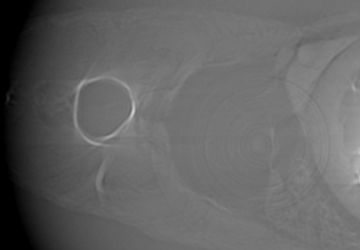

Supplement: Supplementary Dataset 2 [file srep16625-s3.zip › dataset2/0759.tif]

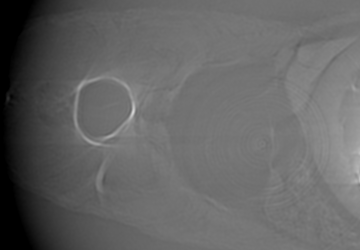

Supplement: Supplementary Dataset 2 [file srep16625-s3.zip › dataset2/0760.tif]

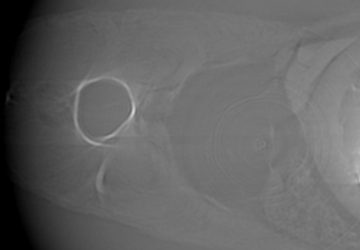

Supplement: Supplementary Dataset 2 [file srep16625-s3.zip › dataset2/0761.tif]
