# Supplementary figures and images for: X-ray phase-contrast tomography for high-spatial-resolution zebrafish muscle imaging (part 3 of 8)
Source: Sci Rep. 2015 Nov 13;5:16625. doi: 10.1038/srep16625 (PMC4643221; doi:10.1038/srep16625)

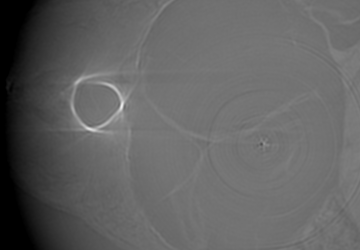

Supplement: Supplementary Dataset 2 [file srep16625-s3.zip › dataset2/0562.tif]

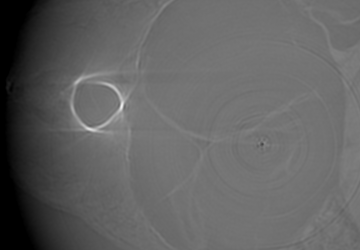

Supplement: Supplementary Dataset 2 [file srep16625-s3.zip › dataset2/0563.tif]

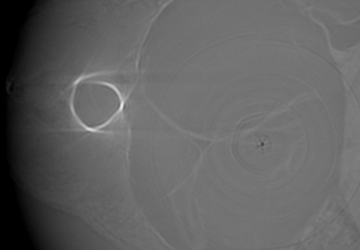

Supplement: Supplementary Dataset 2 [file srep16625-s3.zip › dataset2/0564.tif]

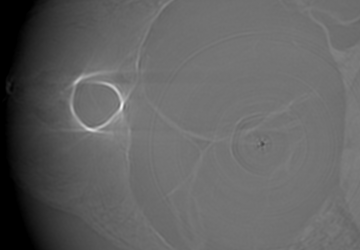

Supplement: Supplementary Dataset 2 [file srep16625-s3.zip › dataset2/0565.tif]

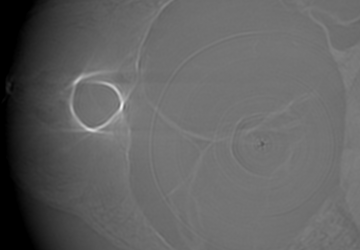

Supplement: Supplementary Dataset 2 [file srep16625-s3.zip › dataset2/0566.tif]

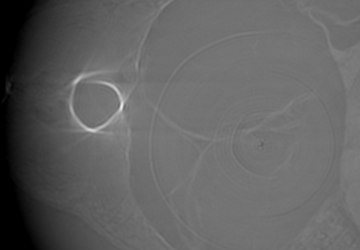

Supplement: Supplementary Dataset 2 [file srep16625-s3.zip › dataset2/0567.tif]

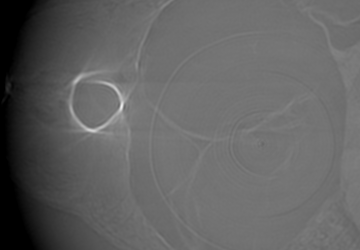

Supplement: Supplementary Dataset 2 [file srep16625-s3.zip › dataset2/0568.tif]

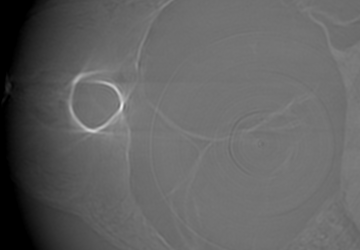

Supplement: Supplementary Dataset 2 [file srep16625-s3.zip › dataset2/0569.tif]

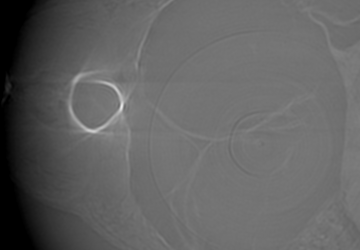

Supplement: Supplementary Dataset 2 [file srep16625-s3.zip › dataset2/0570.tif]

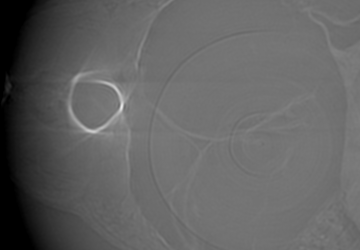

Supplement: Supplementary Dataset 2 [file srep16625-s3.zip › dataset2/0571.tif]

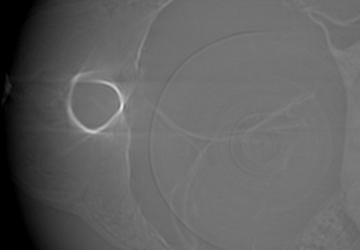

Supplement: Supplementary Dataset 2 [file srep16625-s3.zip › dataset2/0572.tif]

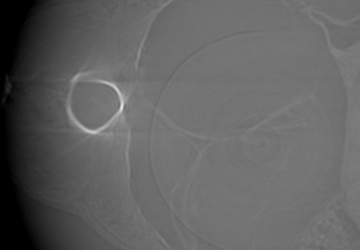

Supplement: Supplementary Dataset 2 [file srep16625-s3.zip › dataset2/0573.tif]

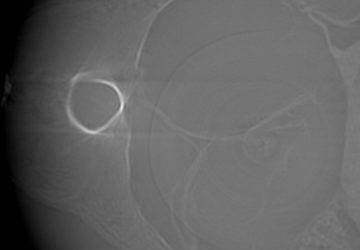

Supplement: Supplementary Dataset 2 [file srep16625-s3.zip › dataset2/0574.tif]

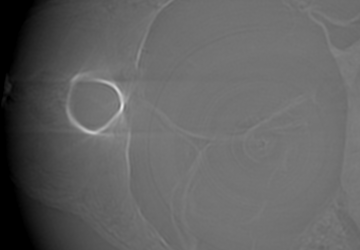

Supplement: Supplementary Dataset 2 [file srep16625-s3.zip › dataset2/0575.tif]

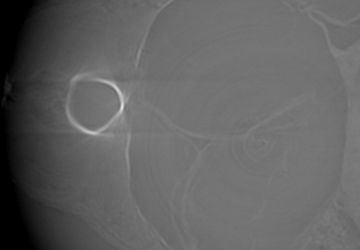

Supplement: Supplementary Dataset 2 [file srep16625-s3.zip › dataset2/0576.tif]

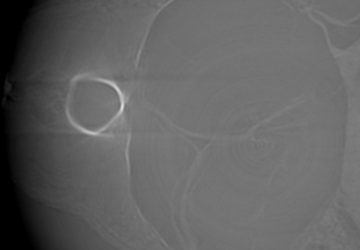

Supplement: Supplementary Dataset 2 [file srep16625-s3.zip › dataset2/0577.tif]

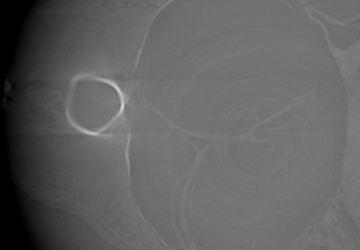

Supplement: Supplementary Dataset 2 [file srep16625-s3.zip › dataset2/0578.tif]

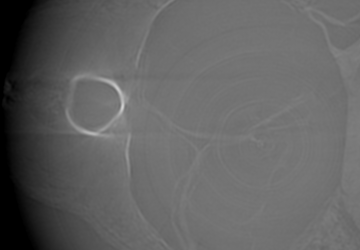

Supplement: Supplementary Dataset 2 [file srep16625-s3.zip › dataset2/0579.tif]

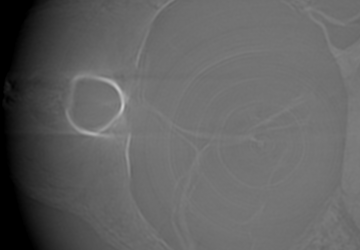

Supplement: Supplementary Dataset 2 [file srep16625-s3.zip › dataset2/0580.tif]

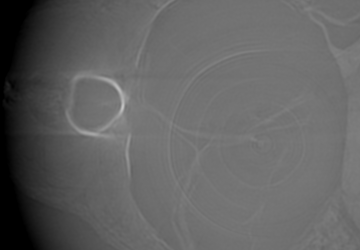

Supplement: Supplementary Dataset 2 [file srep16625-s3.zip › dataset2/0581.tif]

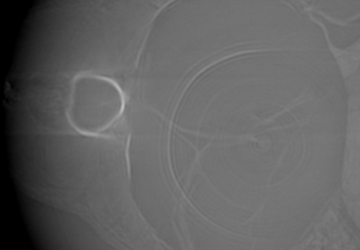

Supplement: Supplementary Dataset 2 [file srep16625-s3.zip › dataset2/0582.tif]

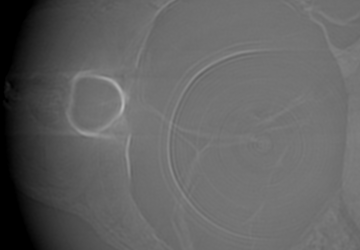

Supplement: Supplementary Dataset 2 [file srep16625-s3.zip › dataset2/0583.tif]

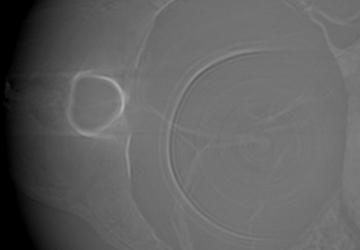

Supplement: Supplementary Dataset 2 [file srep16625-s3.zip › dataset2/0584.tif]

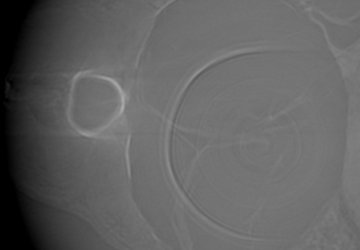

Supplement: Supplementary Dataset 2 [file srep16625-s3.zip › dataset2/0585.tif]

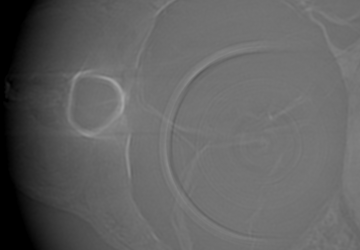

Supplement: Supplementary Dataset 2 [file srep16625-s3.zip › dataset2/0586.tif]

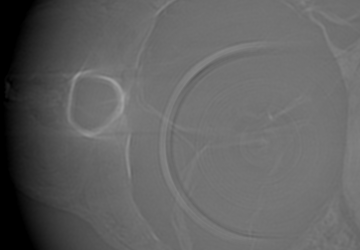

Supplement: Supplementary Dataset 2 [file srep16625-s3.zip › dataset2/0587.tif]

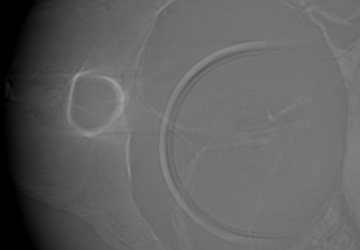

Supplement: Supplementary Dataset 2 [file srep16625-s3.zip › dataset2/0588.tif]

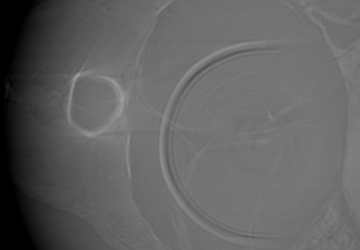

Supplement: Supplementary Dataset 2 [file srep16625-s3.zip › dataset2/0589.tif]

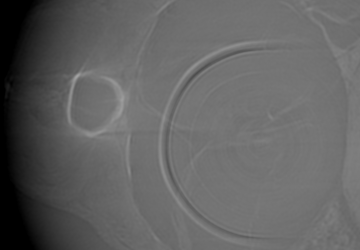

Supplement: Supplementary Dataset 2 [file srep16625-s3.zip › dataset2/0590.tif]

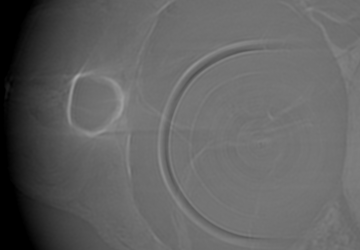

Supplement: Supplementary Dataset 2 [file srep16625-s3.zip › dataset2/0591.tif]

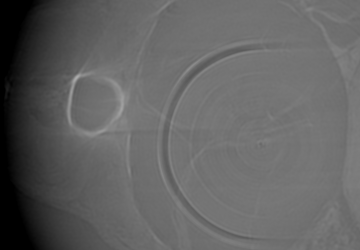

Supplement: Supplementary Dataset 2 [file srep16625-s3.zip › dataset2/0592.tif]

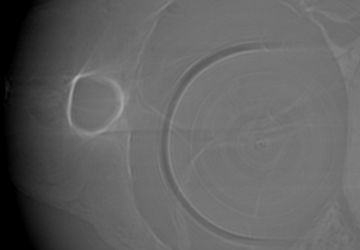

Supplement: Supplementary Dataset 2 [file srep16625-s3.zip › dataset2/0593.tif]

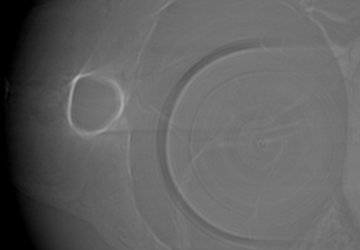

Supplement: Supplementary Dataset 2 [file srep16625-s3.zip › dataset2/0594.tif]

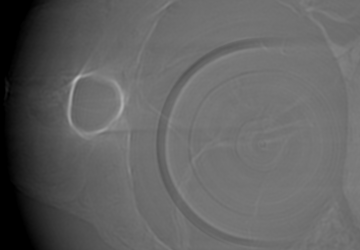

Supplement: Supplementary Dataset 2 [file srep16625-s3.zip › dataset2/0595.tif]

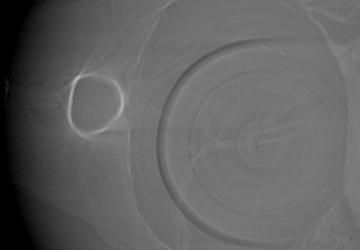

Supplement: Supplementary Dataset 2 [file srep16625-s3.zip › dataset2/0596.tif]

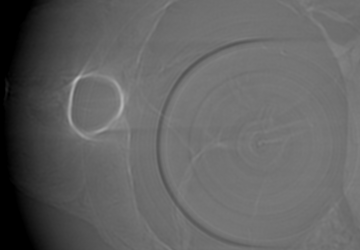

Supplement: Supplementary Dataset 2 [file srep16625-s3.zip › dataset2/0597.tif]

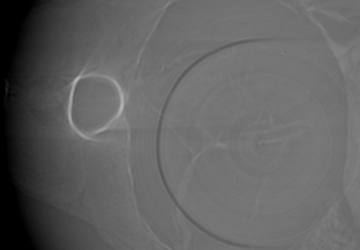

Supplement: Supplementary Dataset 2 [file srep16625-s3.zip › dataset2/0598.tif]

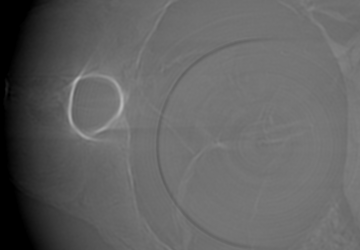

Supplement: Supplementary Dataset 2 [file srep16625-s3.zip › dataset2/0599.tif]

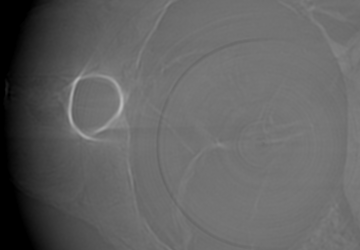

Supplement: Supplementary Dataset 2 [file srep16625-s3.zip › dataset2/0600.tif]

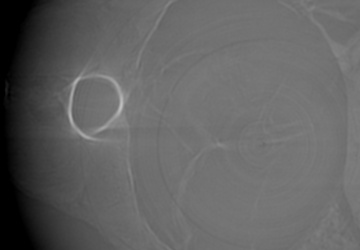

Supplement: Supplementary Dataset 2 [file srep16625-s3.zip › dataset2/0601.tif]

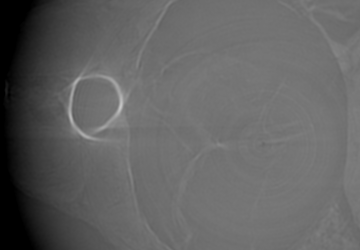

Supplement: Supplementary Dataset 2 [file srep16625-s3.zip › dataset2/0602.tif]

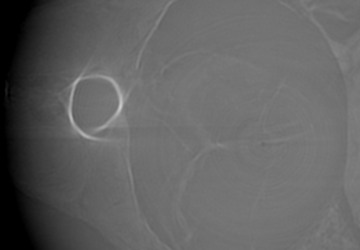

Supplement: Supplementary Dataset 2 [file srep16625-s3.zip › dataset2/0603.tif]

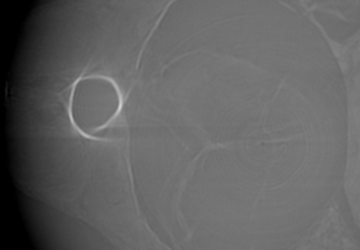

Supplement: Supplementary Dataset 2 [file srep16625-s3.zip › dataset2/0604.tif]

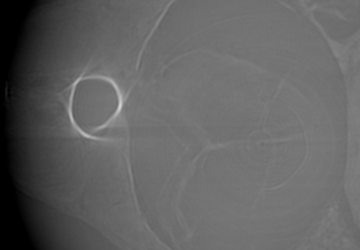

Supplement: Supplementary Dataset 2 [file srep16625-s3.zip › dataset2/0605.tif]

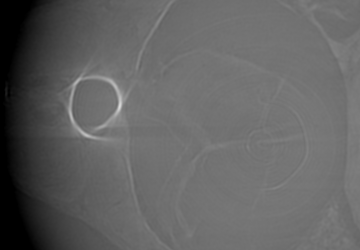

Supplement: Supplementary Dataset 2 [file srep16625-s3.zip › dataset2/0606.tif]

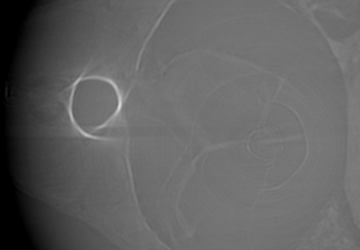

Supplement: Supplementary Dataset 2 [file srep16625-s3.zip › dataset2/0607.tif]

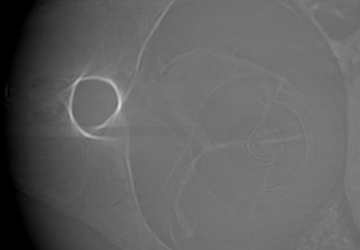

Supplement: Supplementary Dataset 2 [file srep16625-s3.zip › dataset2/0608.tif]

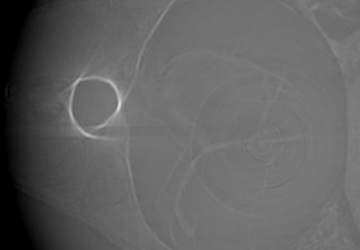

Supplement: Supplementary Dataset 2 [file srep16625-s3.zip › dataset2/0609.tif]

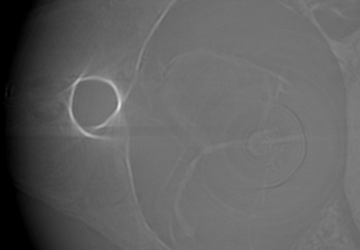

Supplement: Supplementary Dataset 2 [file srep16625-s3.zip › dataset2/0610.tif]

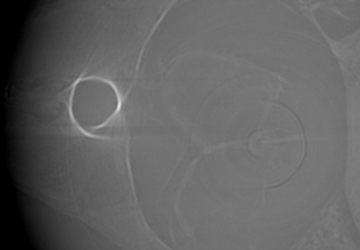

Supplement: Supplementary Dataset 2 [file srep16625-s3.zip › dataset2/0611.tif]

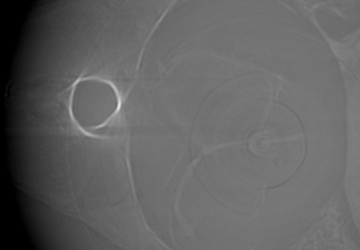

Supplement: Supplementary Dataset 2 [file srep16625-s3.zip › dataset2/0612.tif]

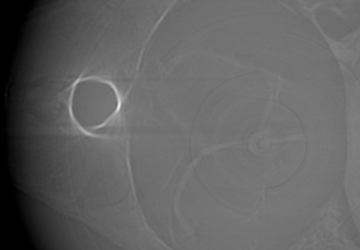

Supplement: Supplementary Dataset 2 [file srep16625-s3.zip › dataset2/0613.tif]

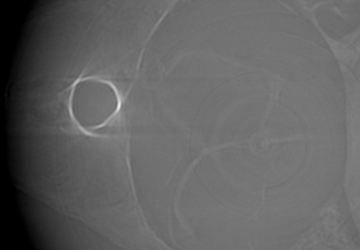

Supplement: Supplementary Dataset 2 [file srep16625-s3.zip › dataset2/0614.tif]

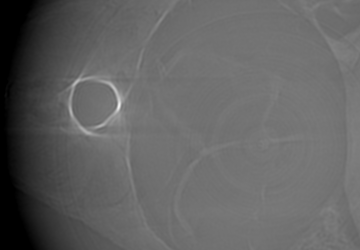

Supplement: Supplementary Dataset 2 [file srep16625-s3.zip › dataset2/0615.tif]

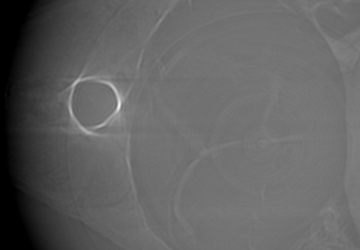

Supplement: Supplementary Dataset 2 [file srep16625-s3.zip › dataset2/0616.tif]

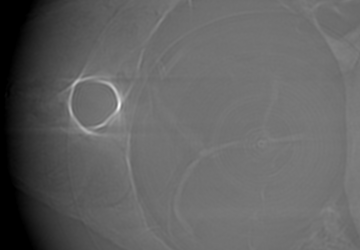

Supplement: Supplementary Dataset 2 [file srep16625-s3.zip › dataset2/0617.tif]

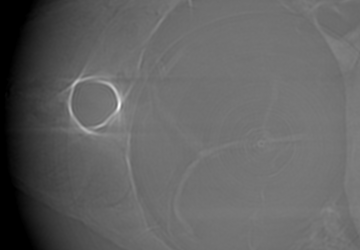

Supplement: Supplementary Dataset 2 [file srep16625-s3.zip › dataset2/0618.tif]

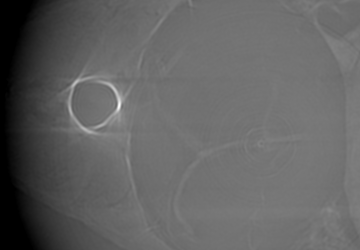

Supplement: Supplementary Dataset 2 [file srep16625-s3.zip › dataset2/0619.tif]

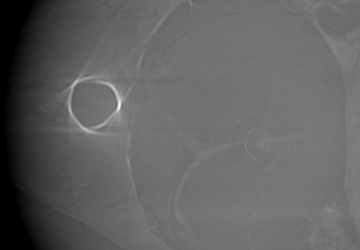

Supplement: Supplementary Dataset 2 [file srep16625-s3.zip › dataset2/0620.tif]

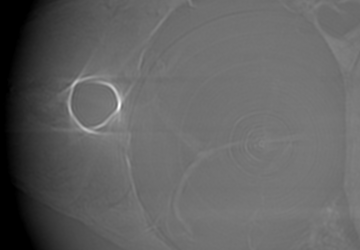

Supplement: Supplementary Dataset 2 [file srep16625-s3.zip › dataset2/0621.tif]

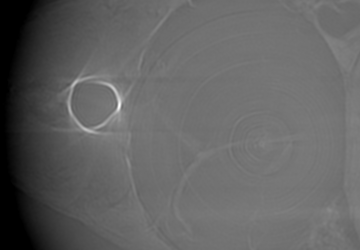

Supplement: Supplementary Dataset 2 [file srep16625-s3.zip › dataset2/0622.tif]

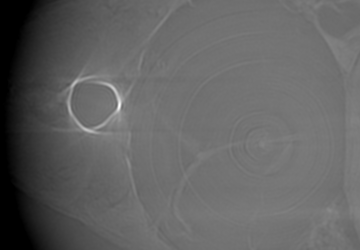

Supplement: Supplementary Dataset 2 [file srep16625-s3.zip › dataset2/0623.tif]

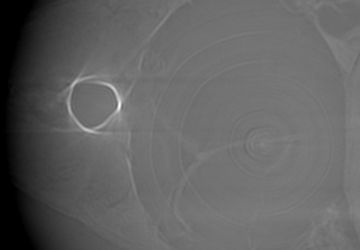

Supplement: Supplementary Dataset 2 [file srep16625-s3.zip › dataset2/0624.tif]

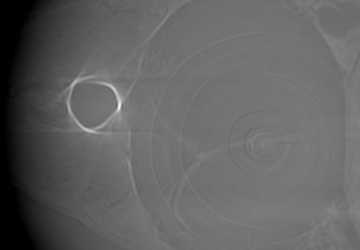

Supplement: Supplementary Dataset 2 [file srep16625-s3.zip › dataset2/0625.tif]

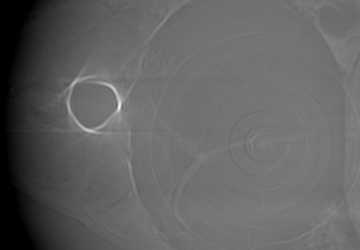

Supplement: Supplementary Dataset 2 [file srep16625-s3.zip › dataset2/0626.tif]

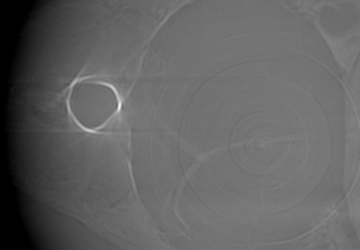

Supplement: Supplementary Dataset 2 [file srep16625-s3.zip › dataset2/0627.tif]

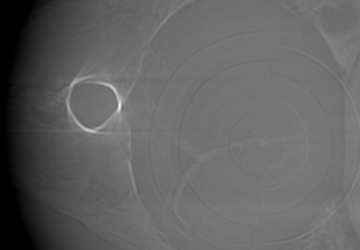

Supplement: Supplementary Dataset 2 [file srep16625-s3.zip › dataset2/0628.tif]

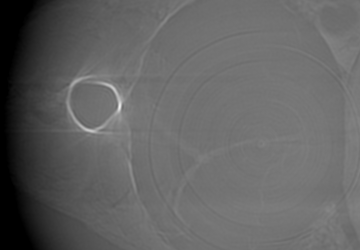

Supplement: Supplementary Dataset 2 [file srep16625-s3.zip › dataset2/0629.tif]

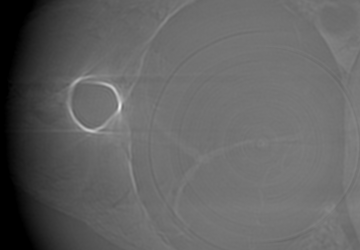

Supplement: Supplementary Dataset 2 [file srep16625-s3.zip › dataset2/0630.tif]

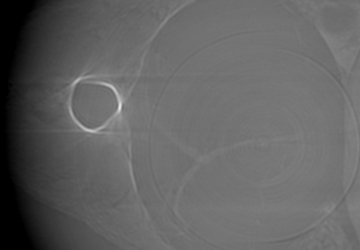

Supplement: Supplementary Dataset 2 [file srep16625-s3.zip › dataset2/0631.tif]

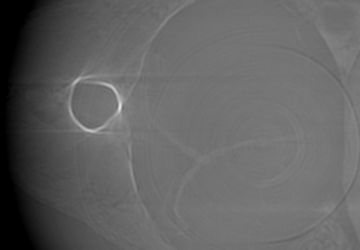

Supplement: Supplementary Dataset 2 [file srep16625-s3.zip › dataset2/0632.tif]

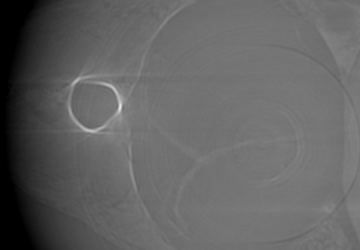

Supplement: Supplementary Dataset 2 [file srep16625-s3.zip › dataset2/0633.tif]

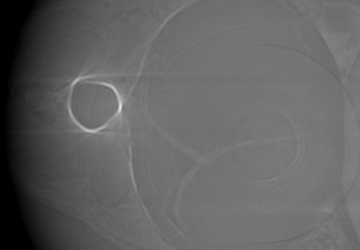

Supplement: Supplementary Dataset 2 [file srep16625-s3.zip › dataset2/0634.tif]

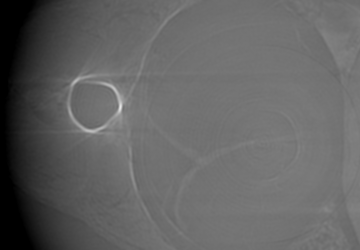

Supplement: Supplementary Dataset 2 [file srep16625-s3.zip › dataset2/0635.tif]

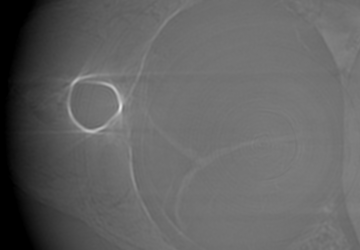

Supplement: Supplementary Dataset 2 [file srep16625-s3.zip › dataset2/0636.tif]

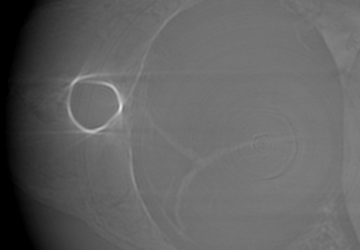

Supplement: Supplementary Dataset 2 [file srep16625-s3.zip › dataset2/0637.tif]

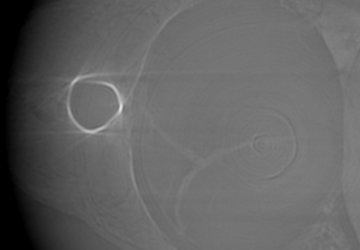

Supplement: Supplementary Dataset 2 [file srep16625-s3.zip › dataset2/0638.tif]

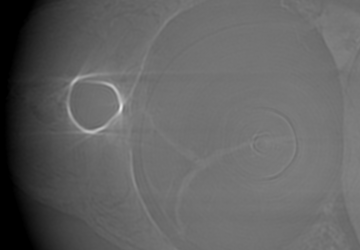

Supplement: Supplementary Dataset 2 [file srep16625-s3.zip › dataset2/0639.tif]

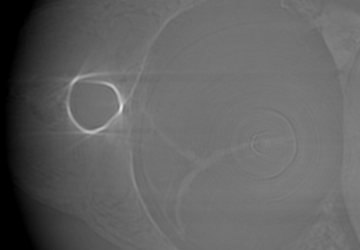

Supplement: Supplementary Dataset 2 [file srep16625-s3.zip › dataset2/0640.tif]

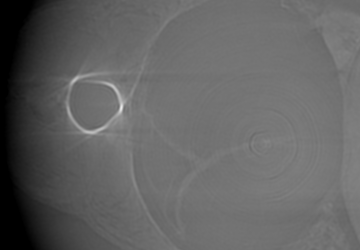

Supplement: Supplementary Dataset 2 [file srep16625-s3.zip › dataset2/0641.tif]

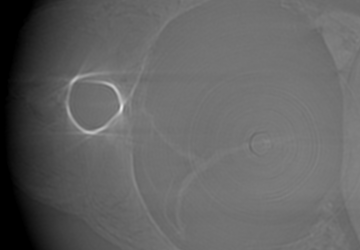

Supplement: Supplementary Dataset 2 [file srep16625-s3.zip › dataset2/0642.tif]

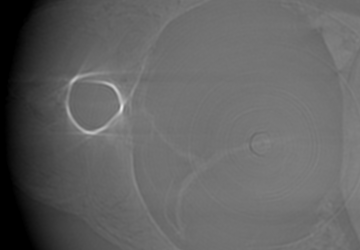

Supplement: Supplementary Dataset 2 [file srep16625-s3.zip › dataset2/0643.tif]

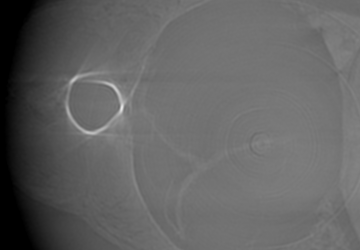

Supplement: Supplementary Dataset 2 [file srep16625-s3.zip › dataset2/0644.tif]

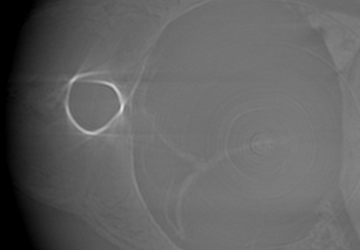

Supplement: Supplementary Dataset 2 [file srep16625-s3.zip › dataset2/0645.tif]

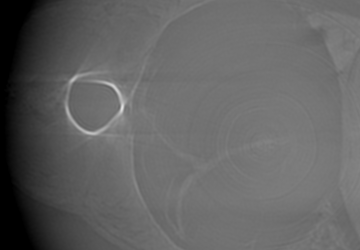

Supplement: Supplementary Dataset 2 [file srep16625-s3.zip › dataset2/0646.tif]

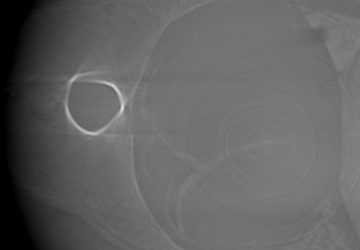

Supplement: Supplementary Dataset 2 [file srep16625-s3.zip › dataset2/0647.tif]

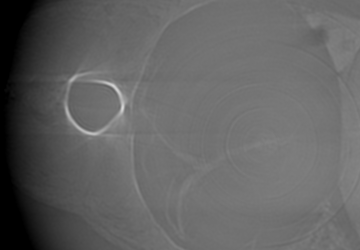

Supplement: Supplementary Dataset 2 [file srep16625-s3.zip › dataset2/0648.tif]

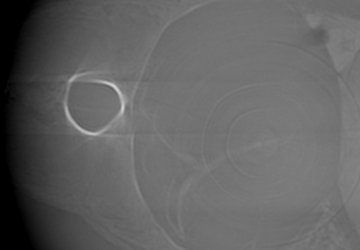

Supplement: Supplementary Dataset 2 [file srep16625-s3.zip › dataset2/0649.tif]

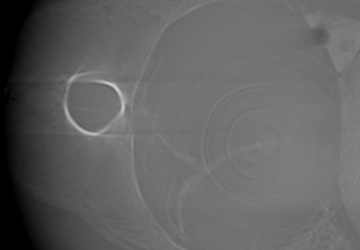

Supplement: Supplementary Dataset 2 [file srep16625-s3.zip › dataset2/0650.tif]

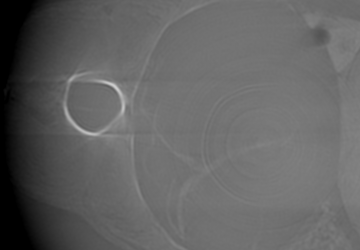

Supplement: Supplementary Dataset 2 [file srep16625-s3.zip › dataset2/0651.tif]

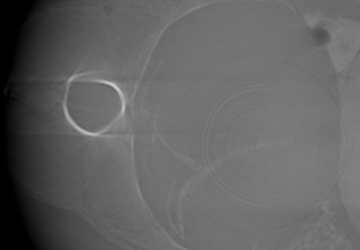

Supplement: Supplementary Dataset 2 [file srep16625-s3.zip › dataset2/0652.tif]

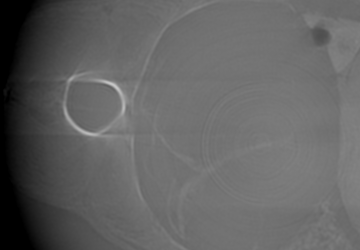

Supplement: Supplementary Dataset 2 [file srep16625-s3.zip › dataset2/0653.tif]

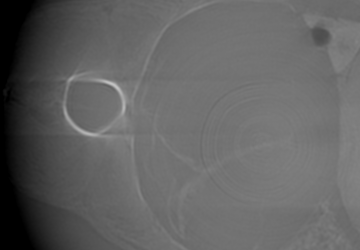

Supplement: Supplementary Dataset 2 [file srep16625-s3.zip › dataset2/0654.tif]

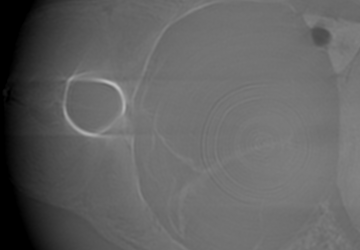

Supplement: Supplementary Dataset 2 [file srep16625-s3.zip › dataset2/0655.tif]

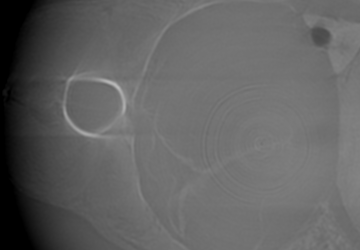

Supplement: Supplementary Dataset 2 [file srep16625-s3.zip › dataset2/0656.tif]

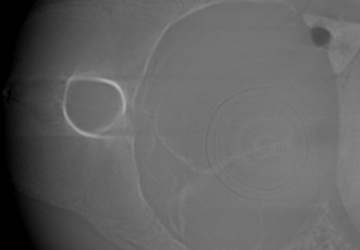

Supplement: Supplementary Dataset 2 [file srep16625-s3.zip › dataset2/0657.tif]

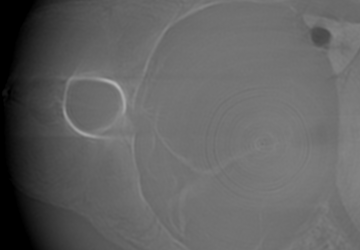

Supplement: Supplementary Dataset 2 [file srep16625-s3.zip › dataset2/0658.tif]

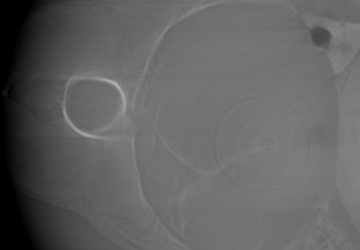

Supplement: Supplementary Dataset 2 [file srep16625-s3.zip › dataset2/0659.tif]

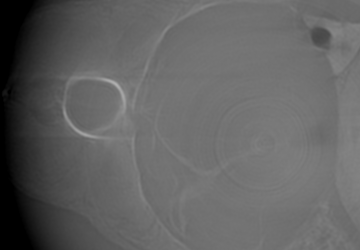

Supplement: Supplementary Dataset 2 [file srep16625-s3.zip › dataset2/0660.tif]

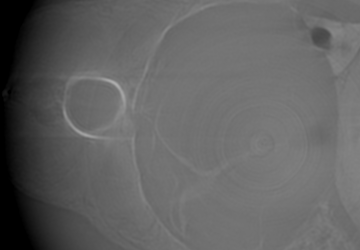

Supplement: Supplementary Dataset 2 [file srep16625-s3.zip › dataset2/0661.tif]
