# Supplementary figures and images for: X-ray phase-contrast tomography for high-spatial-resolution zebrafish muscle imaging (part 6 of 8)
Source: Sci Rep. 2015 Nov 13;5:16625. doi: 10.1038/srep16625 (PMC4643221; doi:10.1038/srep16625)

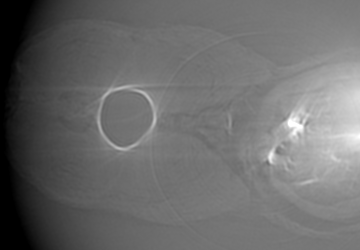

Supplement: Supplementary Dataset 3 [file srep16625-s4.zip › dataset3/0874.tif]

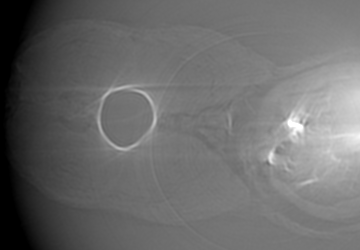

Supplement: Supplementary Dataset 3 [file srep16625-s4.zip › dataset3/0875.tif]

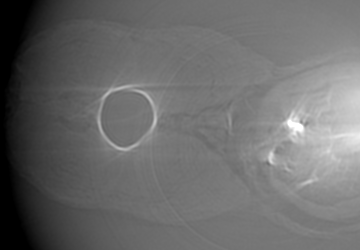

Supplement: Supplementary Dataset 3 [file srep16625-s4.zip › dataset3/0876.tif]

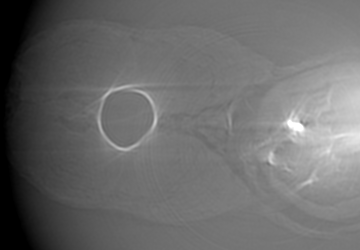

Supplement: Supplementary Dataset 3 [file srep16625-s4.zip › dataset3/0877.tif]

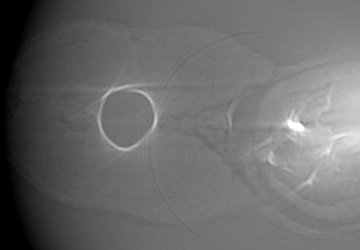

Supplement: Supplementary Dataset 3 [file srep16625-s4.zip › dataset3/0878.tif]

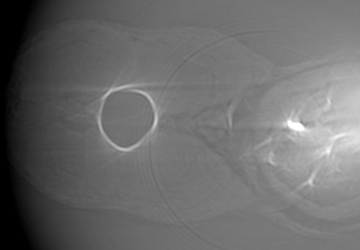

Supplement: Supplementary Dataset 3 [file srep16625-s4.zip › dataset3/0879.tif]

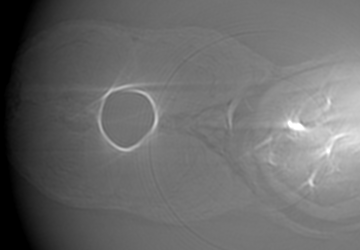

Supplement: Supplementary Dataset 3 [file srep16625-s4.zip › dataset3/0880.tif]

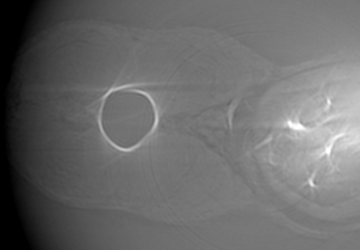

Supplement: Supplementary Dataset 3 [file srep16625-s4.zip › dataset3/0881.tif]

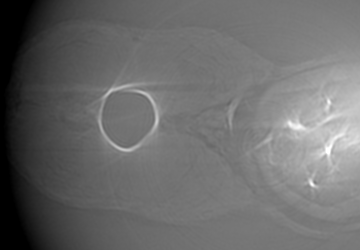

Supplement: Supplementary Dataset 3 [file srep16625-s4.zip › dataset3/0882.tif]

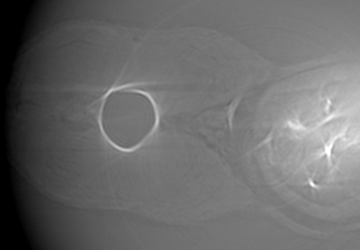

Supplement: Supplementary Dataset 3 [file srep16625-s4.zip › dataset3/0883.tif]

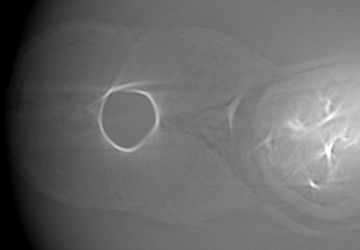

Supplement: Supplementary Dataset 3 [file srep16625-s4.zip › dataset3/0884.tif]

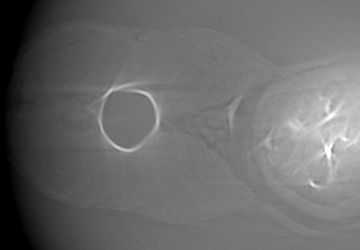

Supplement: Supplementary Dataset 3 [file srep16625-s4.zip › dataset3/0885.tif]

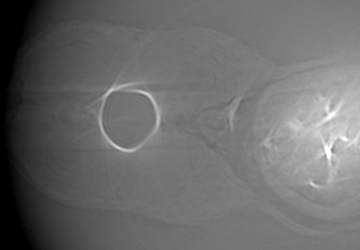

Supplement: Supplementary Dataset 3 [file srep16625-s4.zip › dataset3/0886.tif]

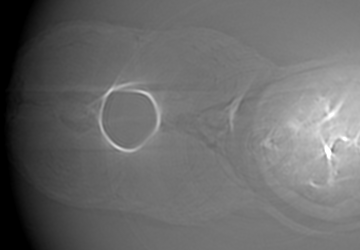

Supplement: Supplementary Dataset 3 [file srep16625-s4.zip › dataset3/0887.tif]

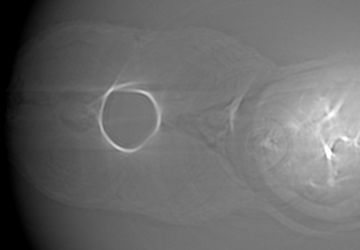

Supplement: Supplementary Dataset 3 [file srep16625-s4.zip › dataset3/0888.tif]

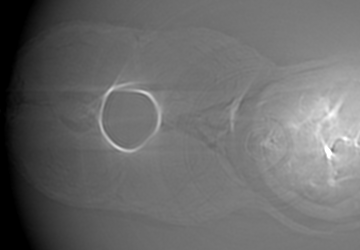

Supplement: Supplementary Dataset 3 [file srep16625-s4.zip › dataset3/0889.tif]

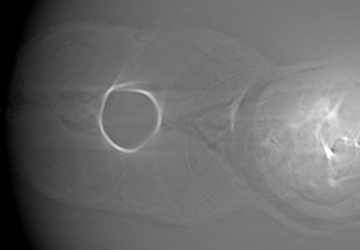

Supplement: Supplementary Dataset 3 [file srep16625-s4.zip › dataset3/0890.tif]

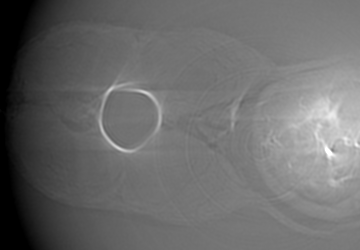

Supplement: Supplementary Dataset 3 [file srep16625-s4.zip › dataset3/0891.tif]

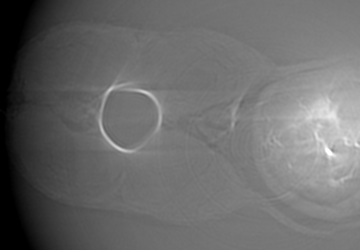

Supplement: Supplementary Dataset 3 [file srep16625-s4.zip › dataset3/0892.tif]

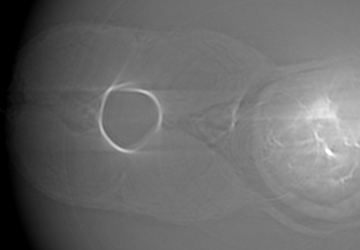

Supplement: Supplementary Dataset 3 [file srep16625-s4.zip › dataset3/0893.tif]

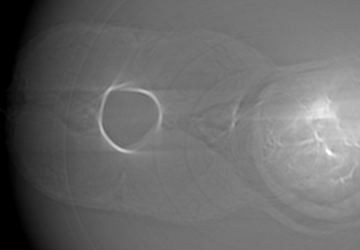

Supplement: Supplementary Dataset 3 [file srep16625-s4.zip › dataset3/0894.tif]

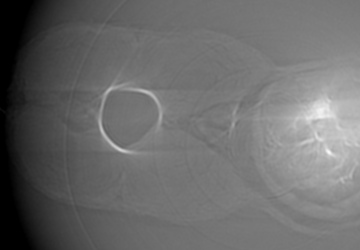

Supplement: Supplementary Dataset 3 [file srep16625-s4.zip › dataset3/0895.tif]

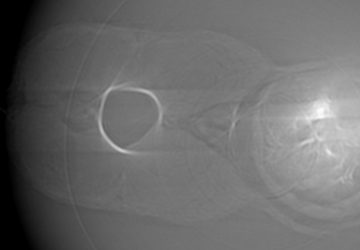

Supplement: Supplementary Dataset 3 [file srep16625-s4.zip › dataset3/0896.tif]

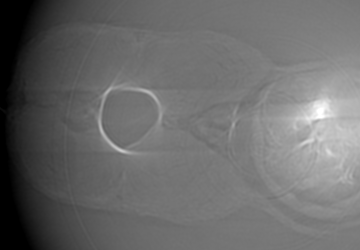

Supplement: Supplementary Dataset 3 [file srep16625-s4.zip › dataset3/0897.tif]

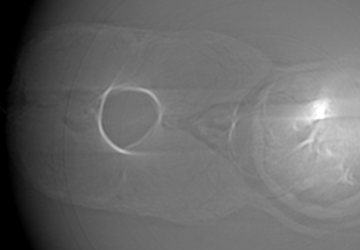

Supplement: Supplementary Dataset 3 [file srep16625-s4.zip › dataset3/0898.tif]

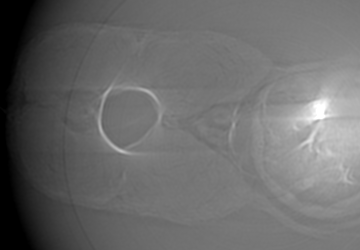

Supplement: Supplementary Dataset 3 [file srep16625-s4.zip › dataset3/0899.tif]

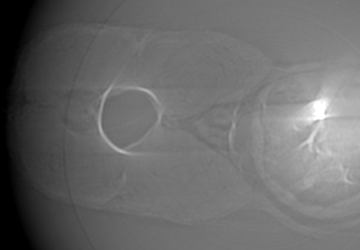

Supplement: Supplementary Dataset 3 [file srep16625-s4.zip › dataset3/0900.tif]

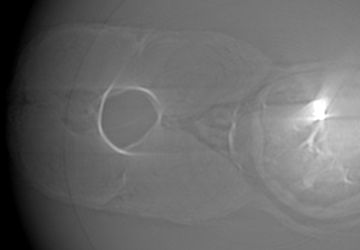

Supplement: Supplementary Dataset 3 [file srep16625-s4.zip › dataset3/0901.tif]

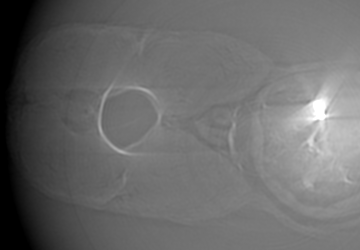

Supplement: Supplementary Dataset 3 [file srep16625-s4.zip › dataset3/0902.tif]

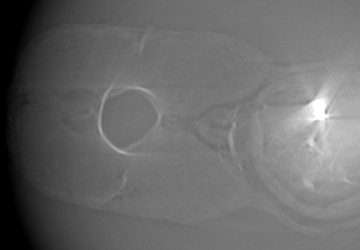

Supplement: Supplementary Dataset 3 [file srep16625-s4.zip › dataset3/0903.tif]

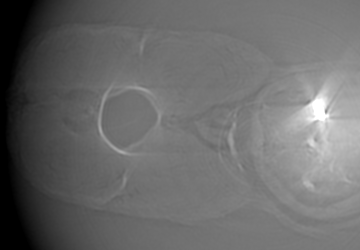

Supplement: Supplementary Dataset 3 [file srep16625-s4.zip › dataset3/0904.tif]

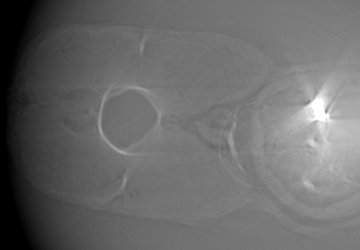

Supplement: Supplementary Dataset 3 [file srep16625-s4.zip › dataset3/0905.tif]

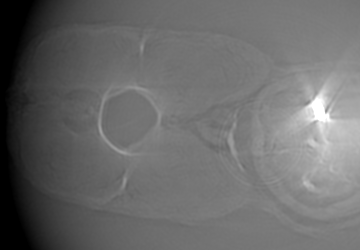

Supplement: Supplementary Dataset 3 [file srep16625-s4.zip › dataset3/0906.tif]

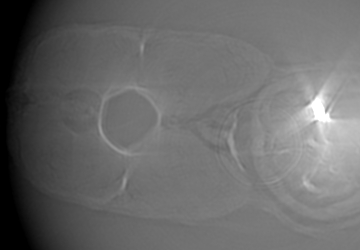

Supplement: Supplementary Dataset 3 [file srep16625-s4.zip › dataset3/0907.tif]

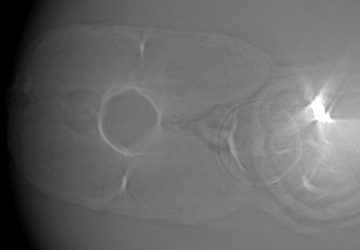

Supplement: Supplementary Dataset 3 [file srep16625-s4.zip › dataset3/0908.tif]

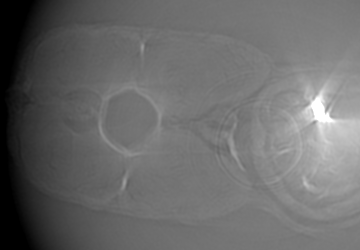

Supplement: Supplementary Dataset 3 [file srep16625-s4.zip › dataset3/0909.tif]

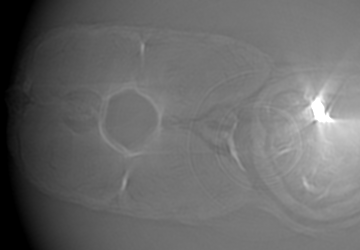

Supplement: Supplementary Dataset 3 [file srep16625-s4.zip › dataset3/0910.tif]

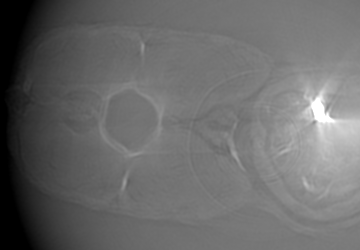

Supplement: Supplementary Dataset 3 [file srep16625-s4.zip › dataset3/0911.tif]

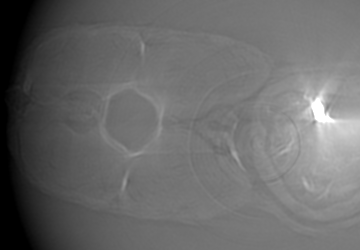

Supplement: Supplementary Dataset 3 [file srep16625-s4.zip › dataset3/0912.tif]

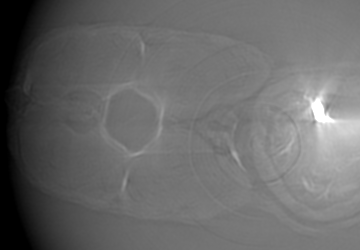

Supplement: Supplementary Dataset 3 [file srep16625-s4.zip › dataset3/0913.tif]

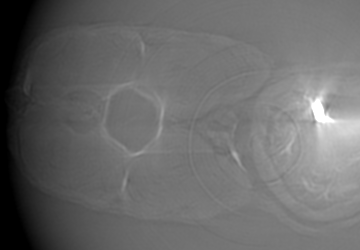

Supplement: Supplementary Dataset 3 [file srep16625-s4.zip › dataset3/0914.tif]

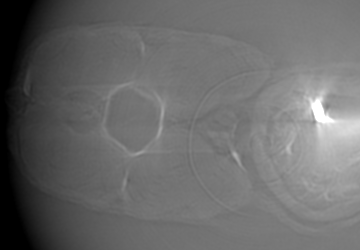

Supplement: Supplementary Dataset 3 [file srep16625-s4.zip › dataset3/0915.tif]

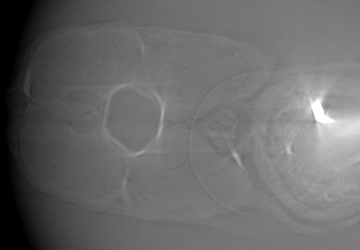

Supplement: Supplementary Dataset 3 [file srep16625-s4.zip › dataset3/0916.tif]

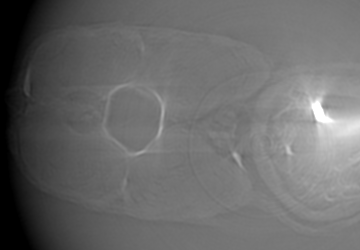

Supplement: Supplementary Dataset 3 [file srep16625-s4.zip › dataset3/0917.tif]

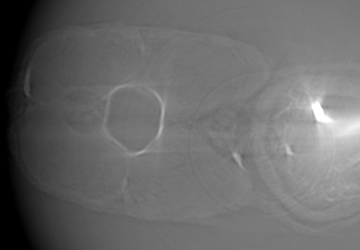

Supplement: Supplementary Dataset 3 [file srep16625-s4.zip › dataset3/0918.tif]

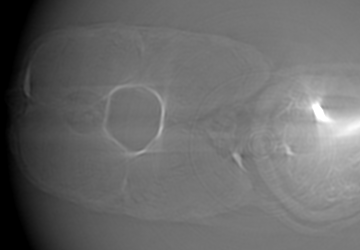

Supplement: Supplementary Dataset 3 [file srep16625-s4.zip › dataset3/0919.tif]

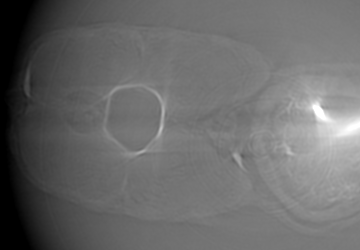

Supplement: Supplementary Dataset 3 [file srep16625-s4.zip › dataset3/0920.tif]

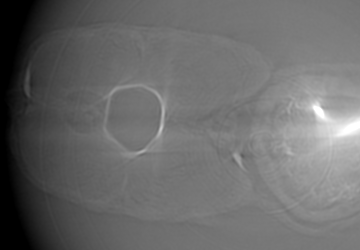

Supplement: Supplementary Dataset 3 [file srep16625-s4.zip › dataset3/0921.tif]

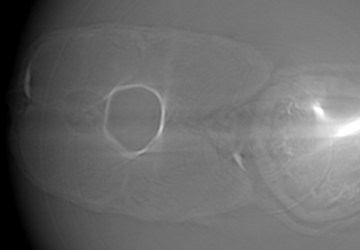

Supplement: Supplementary Dataset 3 [file srep16625-s4.zip › dataset3/0922.tif]

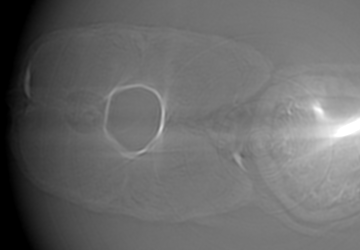

Supplement: Supplementary Dataset 3 [file srep16625-s4.zip › dataset3/0923.tif]

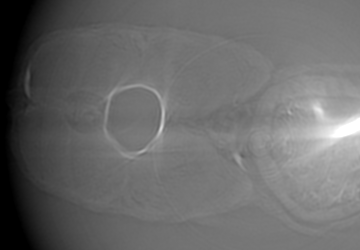

Supplement: Supplementary Dataset 3 [file srep16625-s4.zip › dataset3/0924.tif]

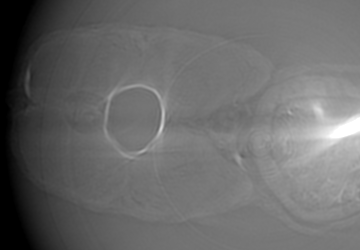

Supplement: Supplementary Dataset 3 [file srep16625-s4.zip › dataset3/0925.tif]

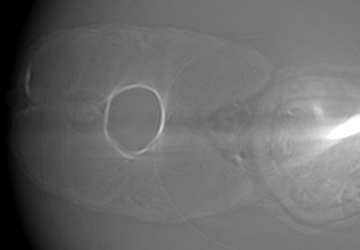

Supplement: Supplementary Dataset 3 [file srep16625-s4.zip › dataset3/0926.tif]

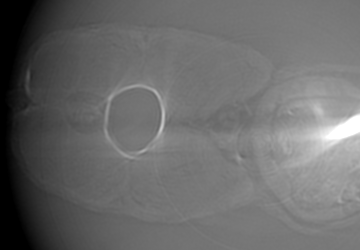

Supplement: Supplementary Dataset 3 [file srep16625-s4.zip › dataset3/0927.tif]

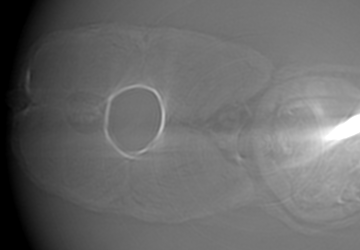

Supplement: Supplementary Dataset 3 [file srep16625-s4.zip › dataset3/0928.tif]

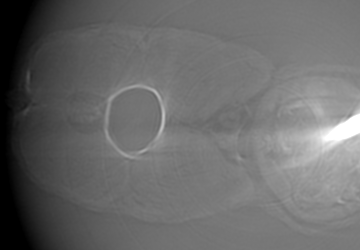

Supplement: Supplementary Dataset 3 [file srep16625-s4.zip › dataset3/0929.tif]

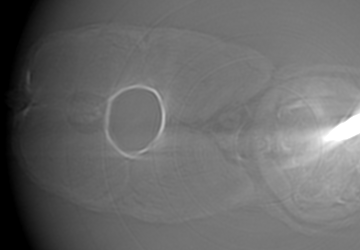

Supplement: Supplementary Dataset 3 [file srep16625-s4.zip › dataset3/0930.tif]

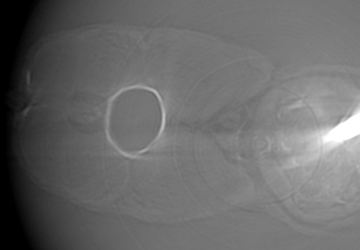

Supplement: Supplementary Dataset 3 [file srep16625-s4.zip › dataset3/0931.tif]

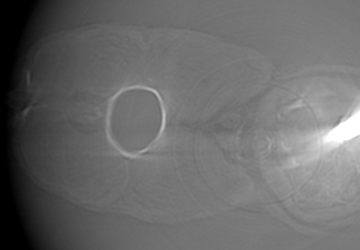

Supplement: Supplementary Dataset 3 [file srep16625-s4.zip › dataset3/0932.tif]

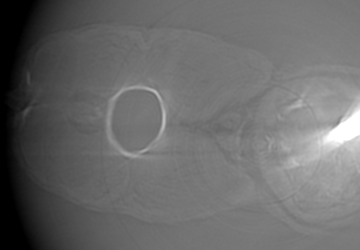

Supplement: Supplementary Dataset 3 [file srep16625-s4.zip › dataset3/0933.tif]

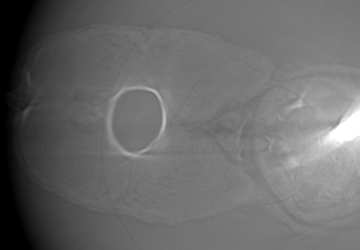

Supplement: Supplementary Dataset 3 [file srep16625-s4.zip › dataset3/0934.tif]

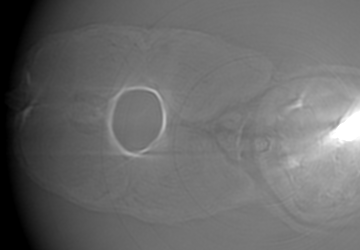

Supplement: Supplementary Dataset 3 [file srep16625-s4.zip › dataset3/0935.tif]

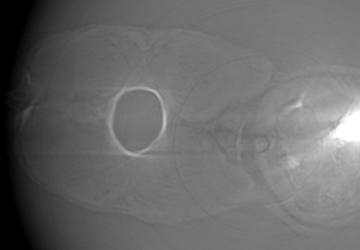

Supplement: Supplementary Dataset 3 [file srep16625-s4.zip › dataset3/0936.tif]

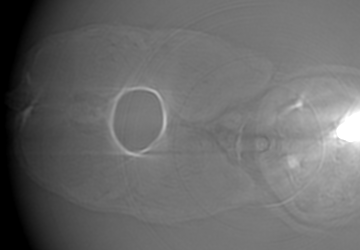

Supplement: Supplementary Dataset 3 [file srep16625-s4.zip › dataset3/0937.tif]

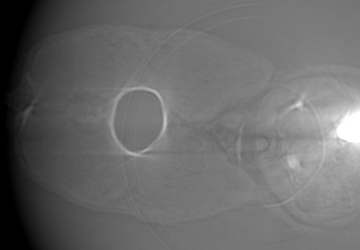

Supplement: Supplementary Dataset 3 [file srep16625-s4.zip › dataset3/0938.tif]

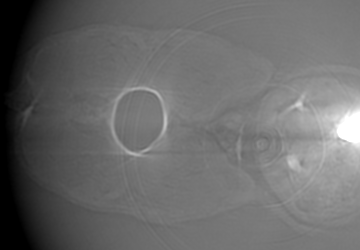

Supplement: Supplementary Dataset 3 [file srep16625-s4.zip › dataset3/0939.tif]

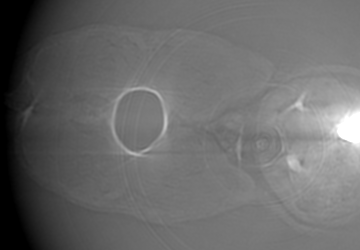

Supplement: Supplementary Dataset 3 [file srep16625-s4.zip › dataset3/0940.tif]

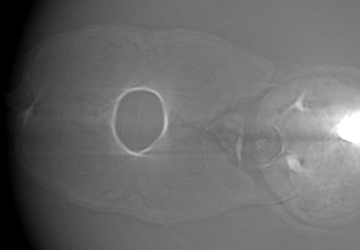

Supplement: Supplementary Dataset 3 [file srep16625-s4.zip › dataset3/0941.tif]

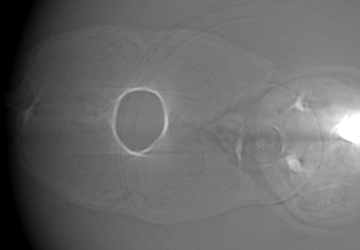

Supplement: Supplementary Dataset 3 [file srep16625-s4.zip › dataset3/0942.tif]

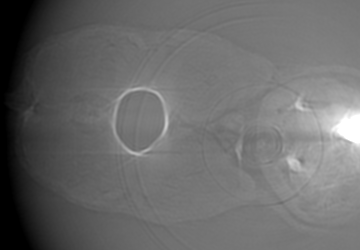

Supplement: Supplementary Dataset 3 [file srep16625-s4.zip › dataset3/0943.tif]

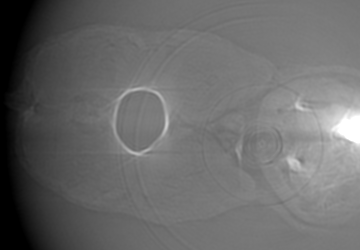

Supplement: Supplementary Dataset 3 [file srep16625-s4.zip › dataset3/0944.tif]

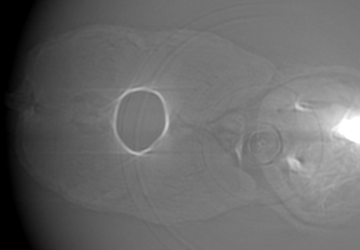

Supplement: Supplementary Dataset 3 [file srep16625-s4.zip › dataset3/0945.tif]

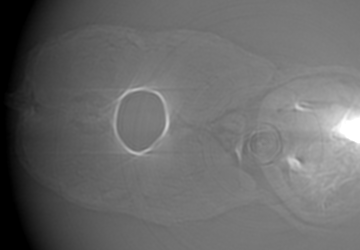

Supplement: Supplementary Dataset 3 [file srep16625-s4.zip › dataset3/0946.tif]

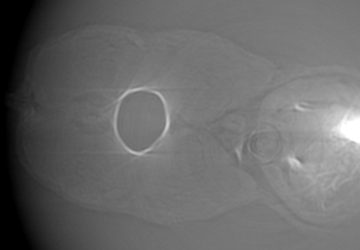

Supplement: Supplementary Dataset 3 [file srep16625-s4.zip › dataset3/0947.tif]

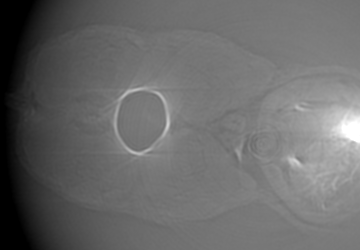

Supplement: Supplementary Dataset 3 [file srep16625-s4.zip › dataset3/0948.tif]

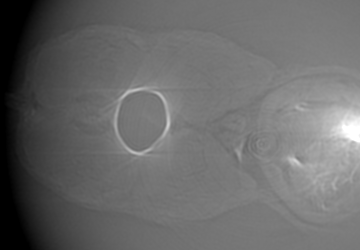

Supplement: Supplementary Dataset 3 [file srep16625-s4.zip › dataset3/0949.tif]

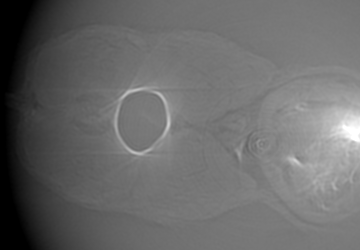

Supplement: Supplementary Dataset 3 [file srep16625-s4.zip › dataset3/0950.tif]

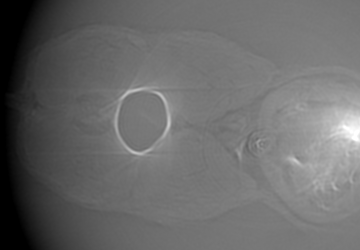

Supplement: Supplementary Dataset 3 [file srep16625-s4.zip › dataset3/0951.tif]

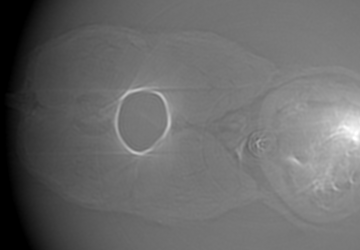

Supplement: Supplementary Dataset 3 [file srep16625-s4.zip › dataset3/0952.tif]

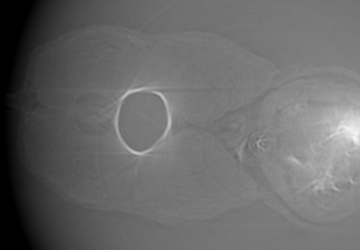

Supplement: Supplementary Dataset 3 [file srep16625-s4.zip › dataset3/0953.tif]

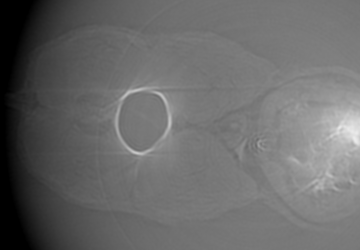

Supplement: Supplementary Dataset 3 [file srep16625-s4.zip › dataset3/0954.tif]

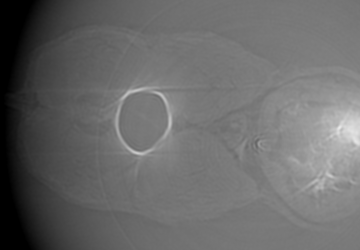

Supplement: Supplementary Dataset 3 [file srep16625-s4.zip › dataset3/0955.tif]

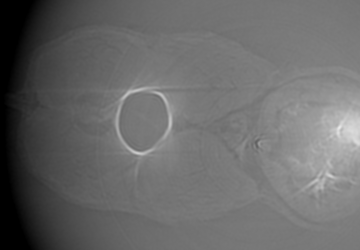

Supplement: Supplementary Dataset 3 [file srep16625-s4.zip › dataset3/0956.tif]

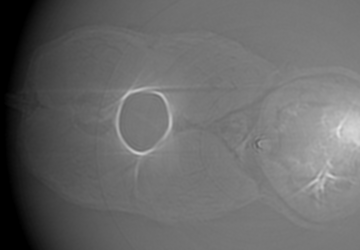

Supplement: Supplementary Dataset 3 [file srep16625-s4.zip › dataset3/0957.tif]

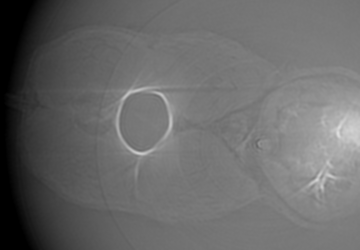

Supplement: Supplementary Dataset 3 [file srep16625-s4.zip › dataset3/0958.tif]

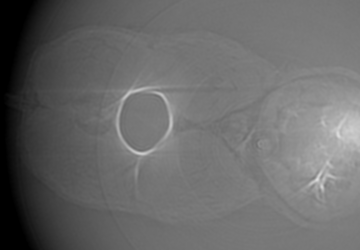

Supplement: Supplementary Dataset 3 [file srep16625-s4.zip › dataset3/0959.tif]

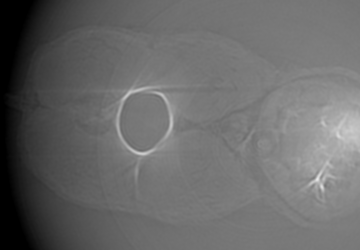

Supplement: Supplementary Dataset 3 [file srep16625-s4.zip › dataset3/0960.tif]

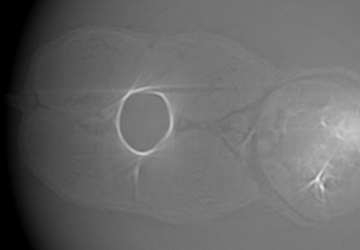

Supplement: Supplementary Dataset 3 [file srep16625-s4.zip › dataset3/0961.tif]

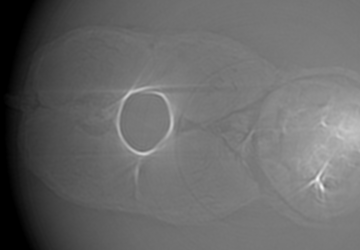

Supplement: Supplementary Dataset 3 [file srep16625-s4.zip › dataset3/0962.tif]

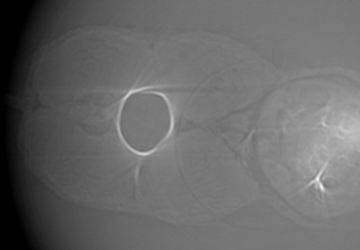

Supplement: Supplementary Dataset 3 [file srep16625-s4.zip › dataset3/0963.tif]

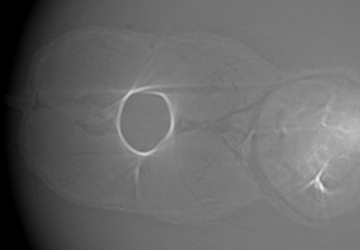

Supplement: Supplementary Dataset 3 [file srep16625-s4.zip › dataset3/0964.tif]

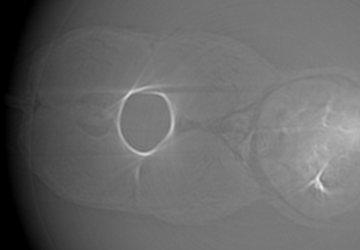

Supplement: Supplementary Dataset 3 [file srep16625-s4.zip › dataset3/0965.tif]

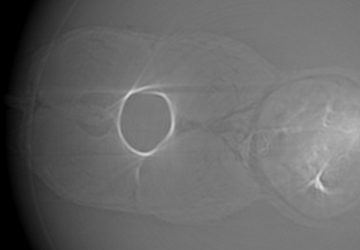

Supplement: Supplementary Dataset 3 [file srep16625-s4.zip › dataset3/0966.tif]

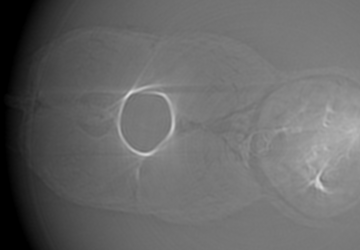

Supplement: Supplementary Dataset 3 [file srep16625-s4.zip › dataset3/0967.tif]

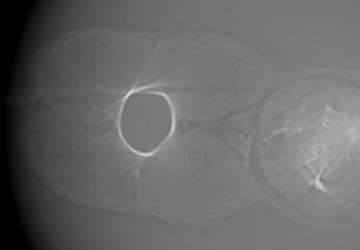

Supplement: Supplementary Dataset 3 [file srep16625-s4.zip › dataset3/0968.tif]

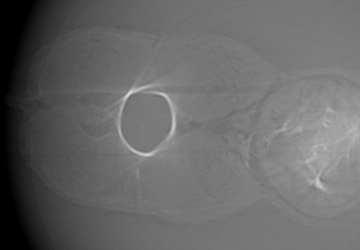

Supplement: Supplementary Dataset 3 [file srep16625-s4.zip › dataset3/0969.tif]

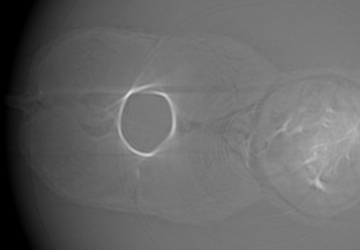

Supplement: Supplementary Dataset 3 [file srep16625-s4.zip › dataset3/0970.tif]

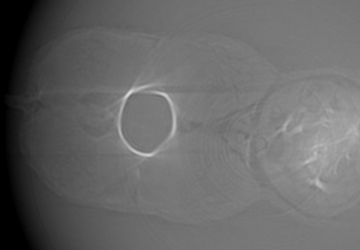

Supplement: Supplementary Dataset 3 [file srep16625-s4.zip › dataset3/0971.tif]

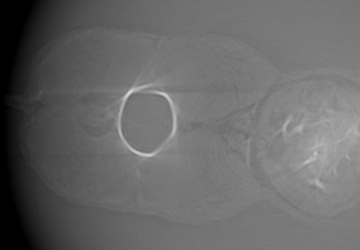

Supplement: Supplementary Dataset 3 [file srep16625-s4.zip › dataset3/0972.tif]

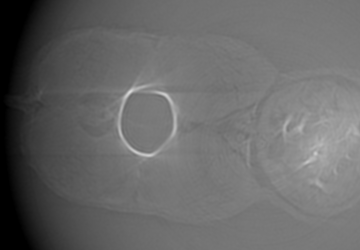

Supplement: Supplementary Dataset 3 [file srep16625-s4.zip › dataset3/0973.tif]
