# Supplementary figures and images for: X-ray phase-contrast tomography for high-spatial-resolution zebrafish muscle imaging (part 5 of 8)
Source: Sci Rep. 2015 Nov 13;5:16625. doi: 10.1038/srep16625 (PMC4643221; doi:10.1038/srep16625)

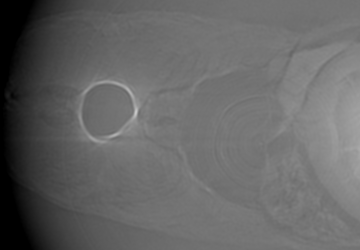

Supplement: Supplementary Dataset 3 [file srep16625-s4.zip › dataset3/0774.tif]

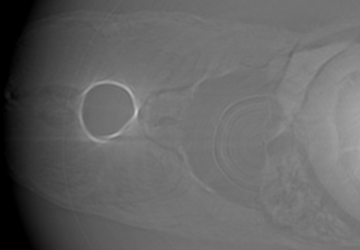

Supplement: Supplementary Dataset 3 [file srep16625-s4.zip › dataset3/0775.tif]

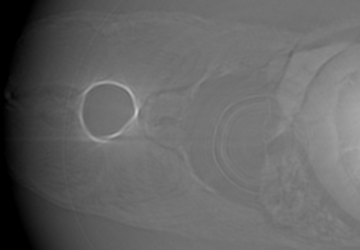

Supplement: Supplementary Dataset 3 [file srep16625-s4.zip › dataset3/0776.tif]

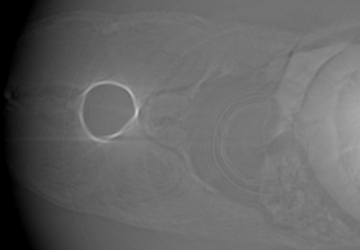

Supplement: Supplementary Dataset 3 [file srep16625-s4.zip › dataset3/0777.tif]

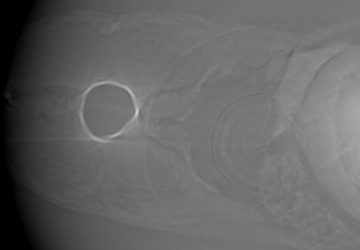

Supplement: Supplementary Dataset 3 [file srep16625-s4.zip › dataset3/0778.tif]

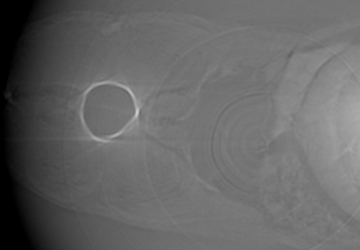

Supplement: Supplementary Dataset 3 [file srep16625-s4.zip › dataset3/0779.tif]

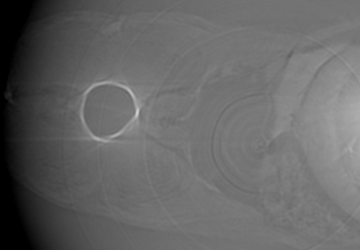

Supplement: Supplementary Dataset 3 [file srep16625-s4.zip › dataset3/0780.tif]

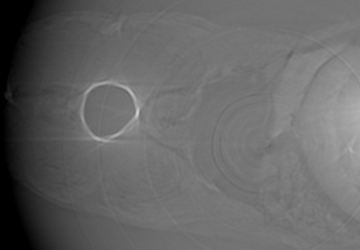

Supplement: Supplementary Dataset 3 [file srep16625-s4.zip › dataset3/0781.tif]

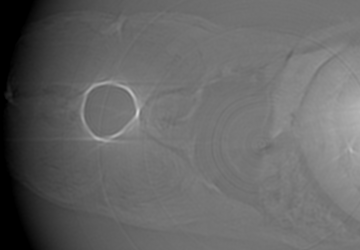

Supplement: Supplementary Dataset 3 [file srep16625-s4.zip › dataset3/0782.tif]

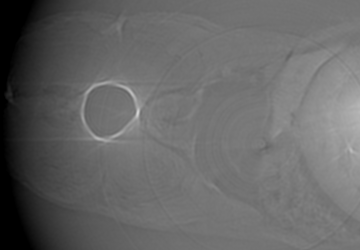

Supplement: Supplementary Dataset 3 [file srep16625-s4.zip › dataset3/0783.tif]

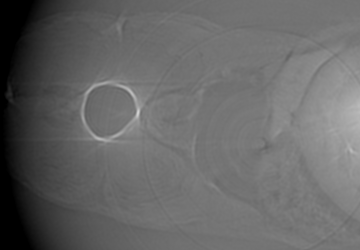

Supplement: Supplementary Dataset 3 [file srep16625-s4.zip › dataset3/0784.tif]

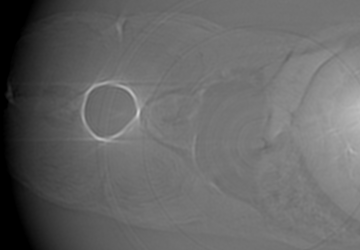

Supplement: Supplementary Dataset 3 [file srep16625-s4.zip › dataset3/0785.tif]

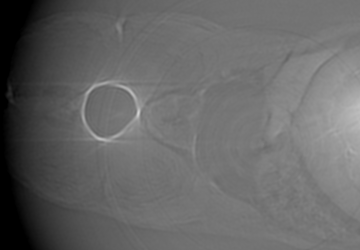

Supplement: Supplementary Dataset 3 [file srep16625-s4.zip › dataset3/0786.tif]

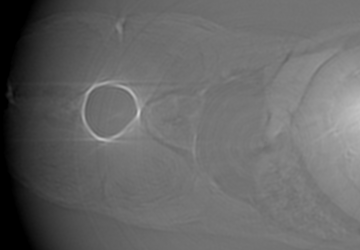

Supplement: Supplementary Dataset 3 [file srep16625-s4.zip › dataset3/0787.tif]

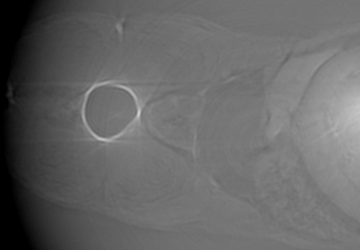

Supplement: Supplementary Dataset 3 [file srep16625-s4.zip › dataset3/0788.tif]

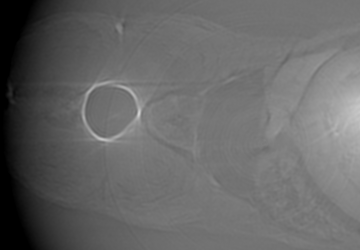

Supplement: Supplementary Dataset 3 [file srep16625-s4.zip › dataset3/0789.tif]

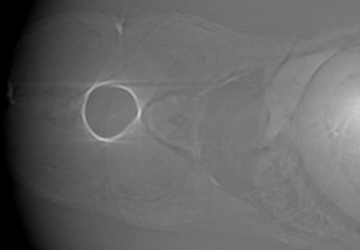

Supplement: Supplementary Dataset 3 [file srep16625-s4.zip › dataset3/0790.tif]

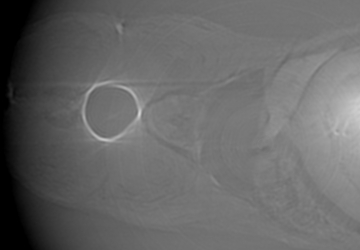

Supplement: Supplementary Dataset 3 [file srep16625-s4.zip › dataset3/0791.tif]

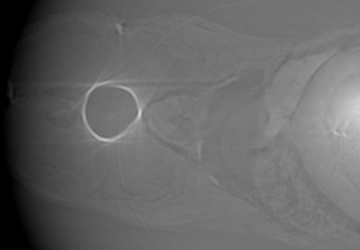

Supplement: Supplementary Dataset 3 [file srep16625-s4.zip › dataset3/0792.tif]

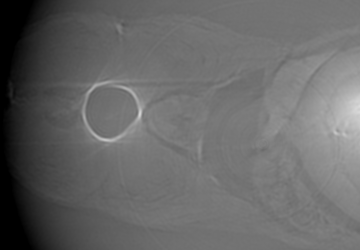

Supplement: Supplementary Dataset 3 [file srep16625-s4.zip › dataset3/0793.tif]

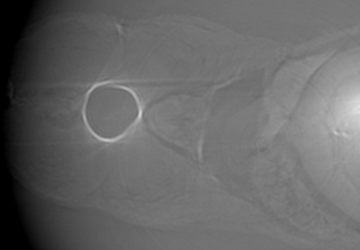

Supplement: Supplementary Dataset 3 [file srep16625-s4.zip › dataset3/0794.tif]

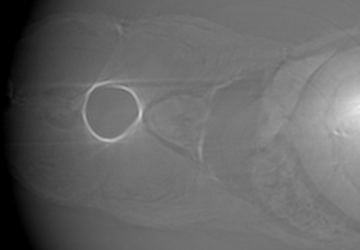

Supplement: Supplementary Dataset 3 [file srep16625-s4.zip › dataset3/0795.tif]

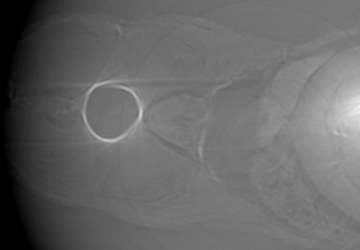

Supplement: Supplementary Dataset 3 [file srep16625-s4.zip › dataset3/0796.tif]

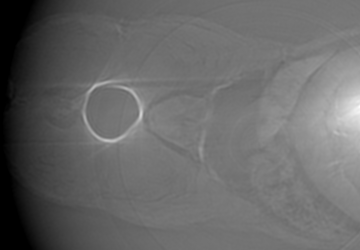

Supplement: Supplementary Dataset 3 [file srep16625-s4.zip › dataset3/0797.tif]

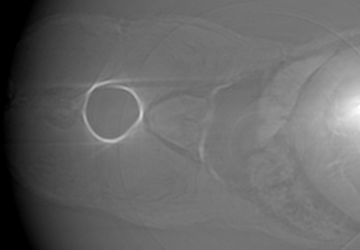

Supplement: Supplementary Dataset 3 [file srep16625-s4.zip › dataset3/0798.tif]

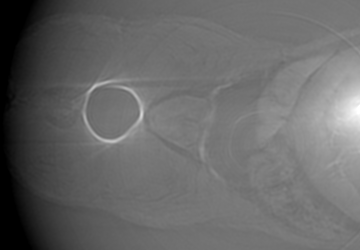

Supplement: Supplementary Dataset 3 [file srep16625-s4.zip › dataset3/0799.tif]

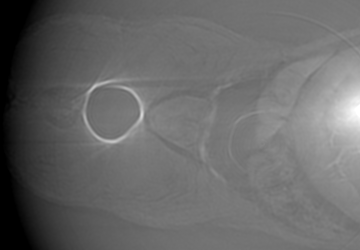

Supplement: Supplementary Dataset 3 [file srep16625-s4.zip › dataset3/0800.tif]

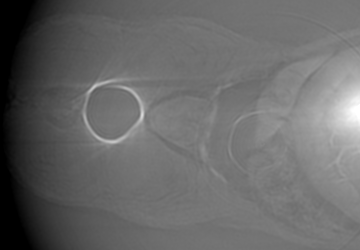

Supplement: Supplementary Dataset 3 [file srep16625-s4.zip › dataset3/0801.tif]

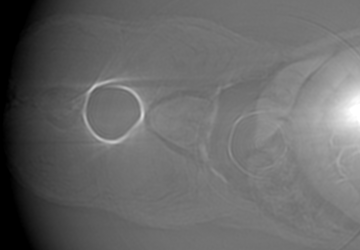

Supplement: Supplementary Dataset 3 [file srep16625-s4.zip › dataset3/0802.tif]

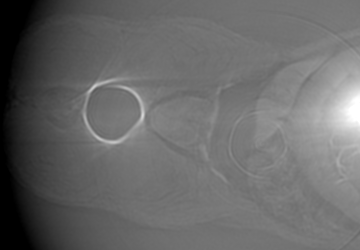

Supplement: Supplementary Dataset 3 [file srep16625-s4.zip › dataset3/0803.tif]

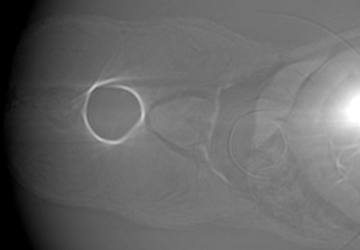

Supplement: Supplementary Dataset 3 [file srep16625-s4.zip › dataset3/0804.tif]

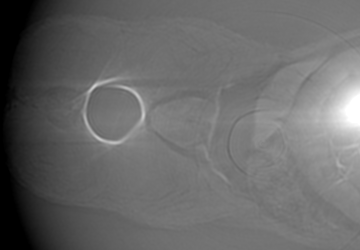

Supplement: Supplementary Dataset 3 [file srep16625-s4.zip › dataset3/0805.tif]

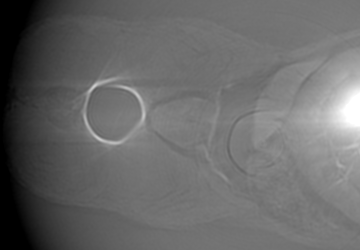

Supplement: Supplementary Dataset 3 [file srep16625-s4.zip › dataset3/0806.tif]

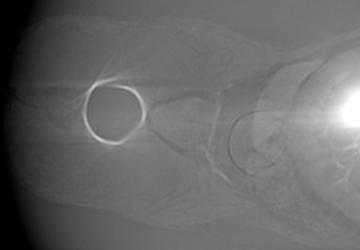

Supplement: Supplementary Dataset 3 [file srep16625-s4.zip › dataset3/0807.tif]

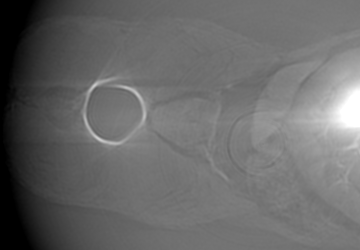

Supplement: Supplementary Dataset 3 [file srep16625-s4.zip › dataset3/0808.tif]

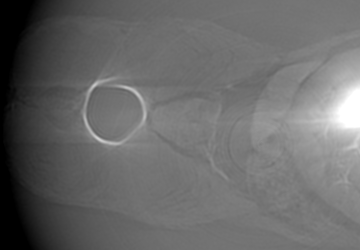

Supplement: Supplementary Dataset 3 [file srep16625-s4.zip › dataset3/0809.tif]

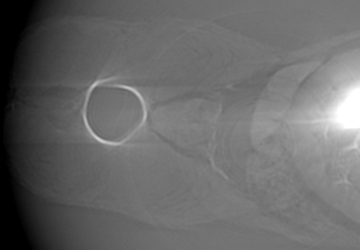

Supplement: Supplementary Dataset 3 [file srep16625-s4.zip › dataset3/0810.tif]

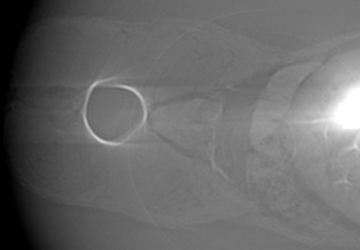

Supplement: Supplementary Dataset 3 [file srep16625-s4.zip › dataset3/0811.tif]

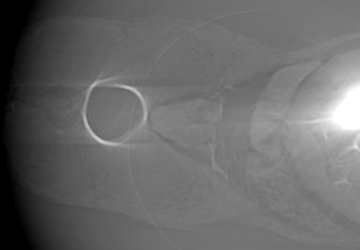

Supplement: Supplementary Dataset 3 [file srep16625-s4.zip › dataset3/0812.tif]

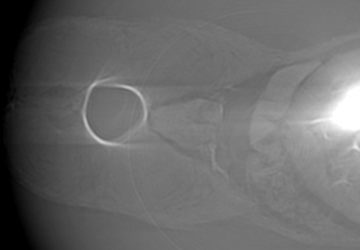

Supplement: Supplementary Dataset 3 [file srep16625-s4.zip › dataset3/0813.tif]

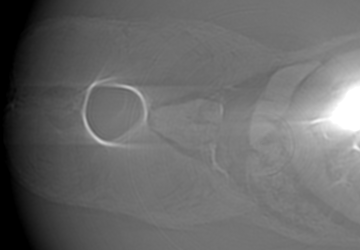

Supplement: Supplementary Dataset 3 [file srep16625-s4.zip › dataset3/0814.tif]

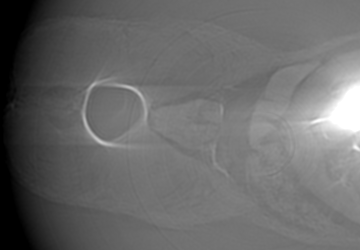

Supplement: Supplementary Dataset 3 [file srep16625-s4.zip › dataset3/0815.tif]

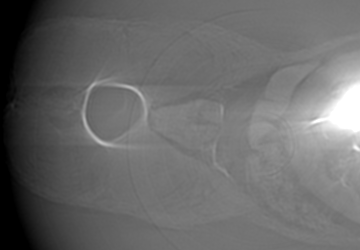

Supplement: Supplementary Dataset 3 [file srep16625-s4.zip › dataset3/0816.tif]

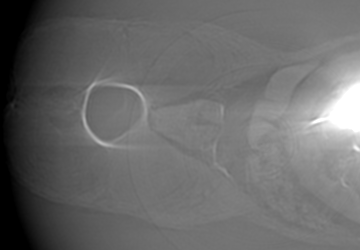

Supplement: Supplementary Dataset 3 [file srep16625-s4.zip › dataset3/0817.tif]

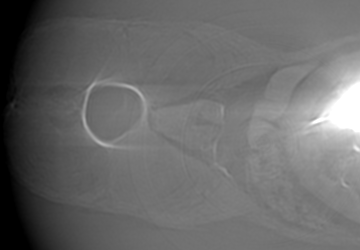

Supplement: Supplementary Dataset 3 [file srep16625-s4.zip › dataset3/0818.tif]

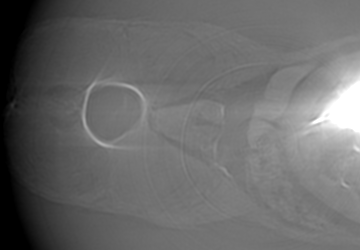

Supplement: Supplementary Dataset 3 [file srep16625-s4.zip › dataset3/0819.tif]

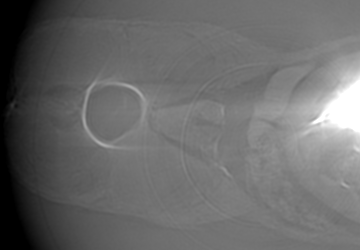

Supplement: Supplementary Dataset 3 [file srep16625-s4.zip › dataset3/0820.tif]

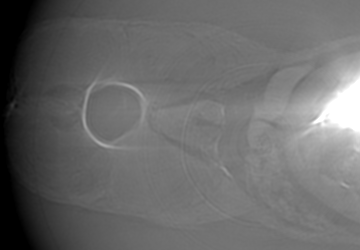

Supplement: Supplementary Dataset 3 [file srep16625-s4.zip › dataset3/0821.tif]

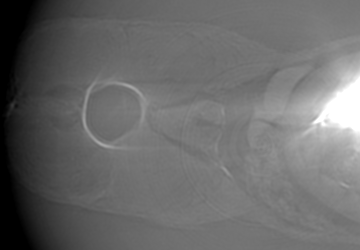

Supplement: Supplementary Dataset 3 [file srep16625-s4.zip › dataset3/0822.tif]

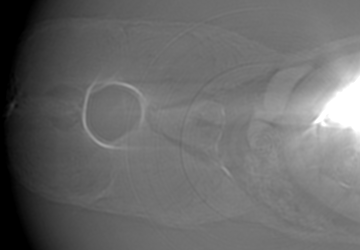

Supplement: Supplementary Dataset 3 [file srep16625-s4.zip › dataset3/0823.tif]

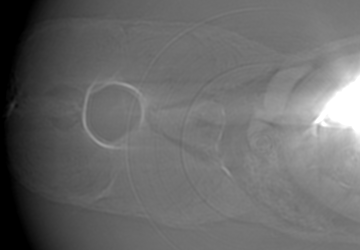

Supplement: Supplementary Dataset 3 [file srep16625-s4.zip › dataset3/0824.tif]

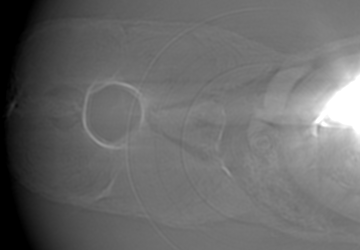

Supplement: Supplementary Dataset 3 [file srep16625-s4.zip › dataset3/0825.tif]

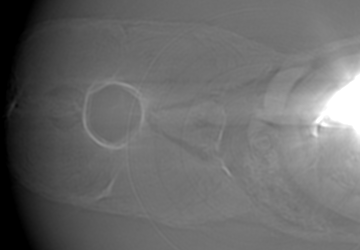

Supplement: Supplementary Dataset 3 [file srep16625-s4.zip › dataset3/0826.tif]

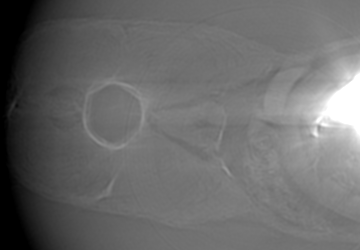

Supplement: Supplementary Dataset 3 [file srep16625-s4.zip › dataset3/0827.tif]

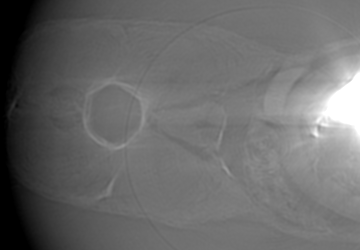

Supplement: Supplementary Dataset 3 [file srep16625-s4.zip › dataset3/0828.tif]

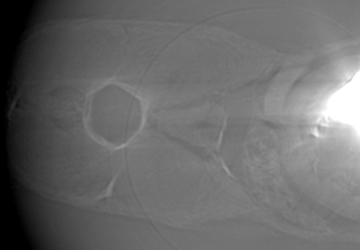

Supplement: Supplementary Dataset 3 [file srep16625-s4.zip › dataset3/0829.tif]

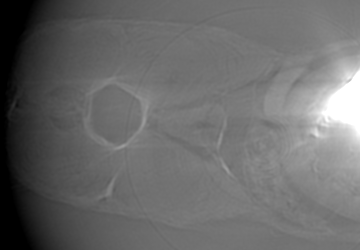

Supplement: Supplementary Dataset 3 [file srep16625-s4.zip › dataset3/0830.tif]

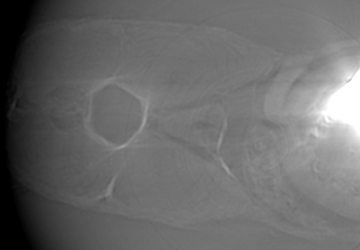

Supplement: Supplementary Dataset 3 [file srep16625-s4.zip › dataset3/0831.tif]

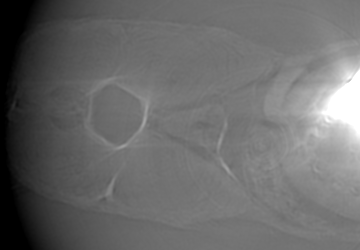

Supplement: Supplementary Dataset 3 [file srep16625-s4.zip › dataset3/0832.tif]

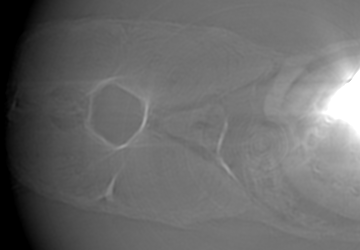

Supplement: Supplementary Dataset 3 [file srep16625-s4.zip › dataset3/0833.tif]

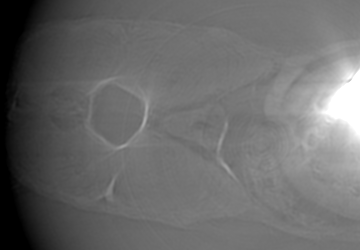

Supplement: Supplementary Dataset 3 [file srep16625-s4.zip › dataset3/0834.tif]

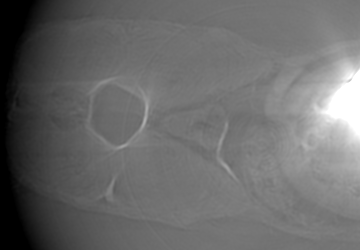

Supplement: Supplementary Dataset 3 [file srep16625-s4.zip › dataset3/0835.tif]

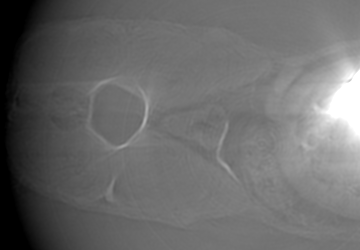

Supplement: Supplementary Dataset 3 [file srep16625-s4.zip › dataset3/0836.tif]

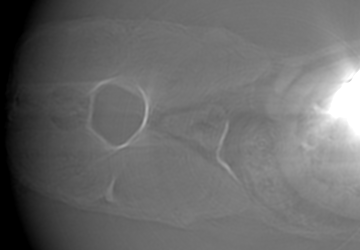

Supplement: Supplementary Dataset 3 [file srep16625-s4.zip › dataset3/0837.tif]

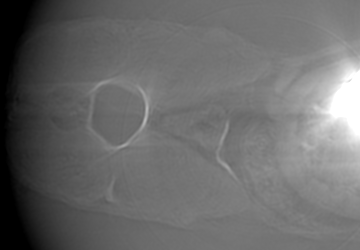

Supplement: Supplementary Dataset 3 [file srep16625-s4.zip › dataset3/0838.tif]

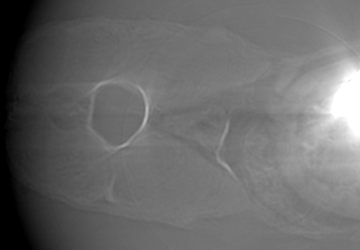

Supplement: Supplementary Dataset 3 [file srep16625-s4.zip › dataset3/0839.tif]

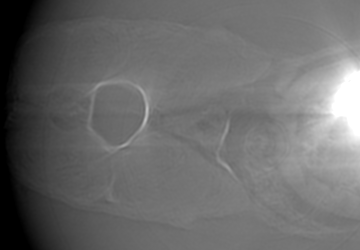

Supplement: Supplementary Dataset 3 [file srep16625-s4.zip › dataset3/0840.tif]

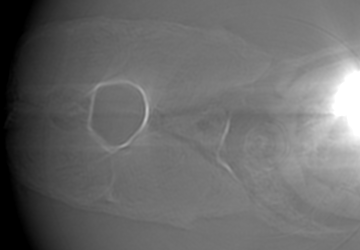

Supplement: Supplementary Dataset 3 [file srep16625-s4.zip › dataset3/0841.tif]

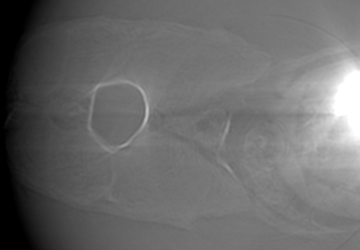

Supplement: Supplementary Dataset 3 [file srep16625-s4.zip › dataset3/0842.tif]

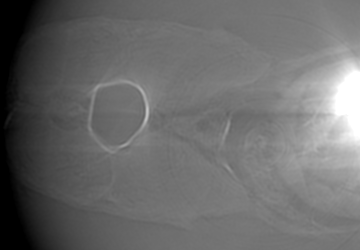

Supplement: Supplementary Dataset 3 [file srep16625-s4.zip › dataset3/0843.tif]

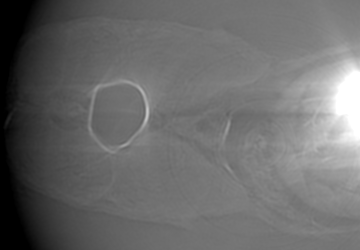

Supplement: Supplementary Dataset 3 [file srep16625-s4.zip › dataset3/0844.tif]

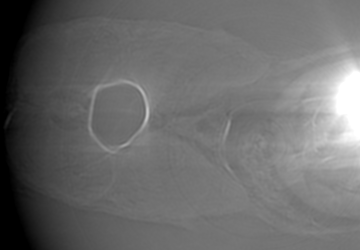

Supplement: Supplementary Dataset 3 [file srep16625-s4.zip › dataset3/0845.tif]

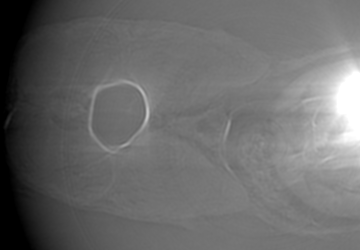

Supplement: Supplementary Dataset 3 [file srep16625-s4.zip › dataset3/0846.tif]

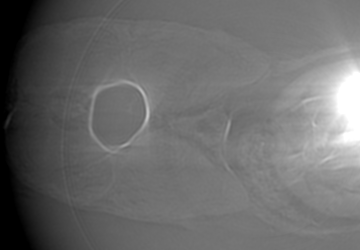

Supplement: Supplementary Dataset 3 [file srep16625-s4.zip › dataset3/0847.tif]

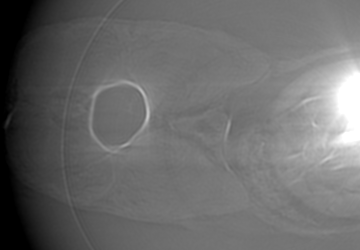

Supplement: Supplementary Dataset 3 [file srep16625-s4.zip › dataset3/0848.tif]

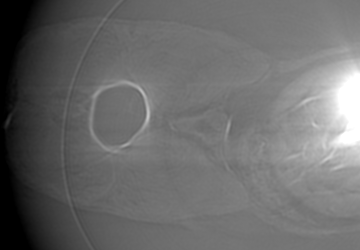

Supplement: Supplementary Dataset 3 [file srep16625-s4.zip › dataset3/0849.tif]

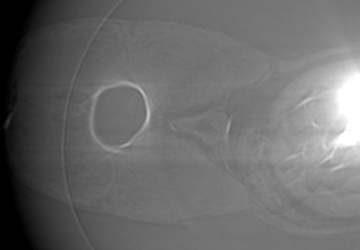

Supplement: Supplementary Dataset 3 [file srep16625-s4.zip › dataset3/0850.tif]

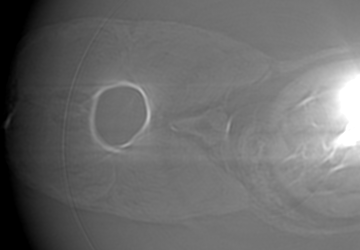

Supplement: Supplementary Dataset 3 [file srep16625-s4.zip › dataset3/0851.tif]

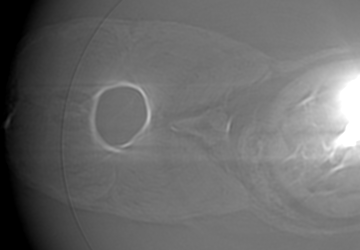

Supplement: Supplementary Dataset 3 [file srep16625-s4.zip › dataset3/0852.tif]

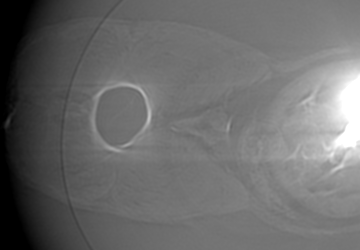

Supplement: Supplementary Dataset 3 [file srep16625-s4.zip › dataset3/0853.tif]

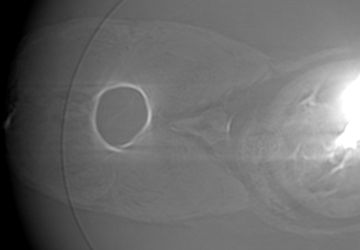

Supplement: Supplementary Dataset 3 [file srep16625-s4.zip › dataset3/0854.tif]

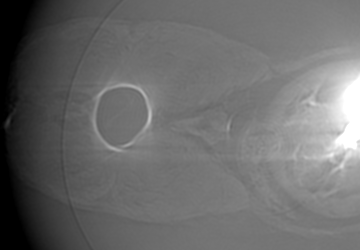

Supplement: Supplementary Dataset 3 [file srep16625-s4.zip › dataset3/0855.tif]

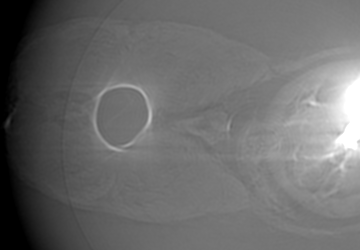

Supplement: Supplementary Dataset 3 [file srep16625-s4.zip › dataset3/0856.tif]

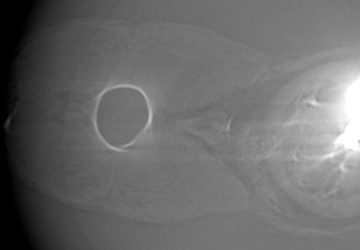

Supplement: Supplementary Dataset 3 [file srep16625-s4.zip › dataset3/0857.tif]

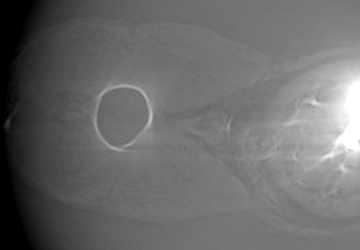

Supplement: Supplementary Dataset 3 [file srep16625-s4.zip › dataset3/0858.tif]

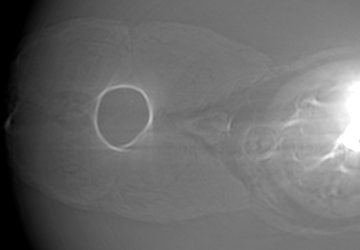

Supplement: Supplementary Dataset 3 [file srep16625-s4.zip › dataset3/0859.tif]

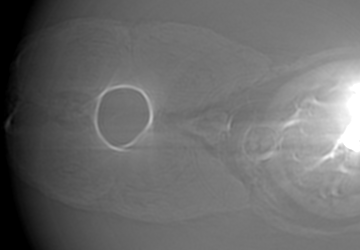

Supplement: Supplementary Dataset 3 [file srep16625-s4.zip › dataset3/0860.tif]

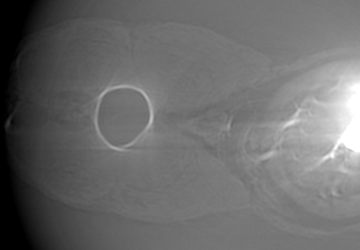

Supplement: Supplementary Dataset 3 [file srep16625-s4.zip › dataset3/0861.tif]

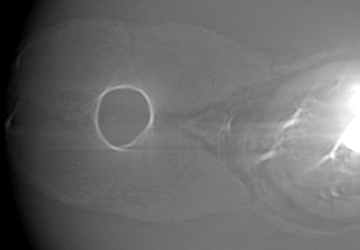

Supplement: Supplementary Dataset 3 [file srep16625-s4.zip › dataset3/0862.tif]

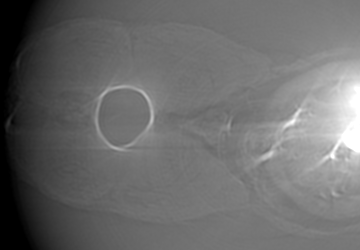

Supplement: Supplementary Dataset 3 [file srep16625-s4.zip › dataset3/0863.tif]

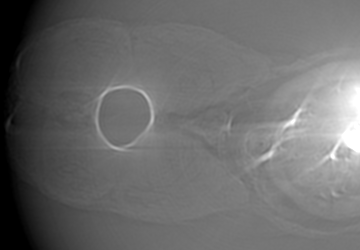

Supplement: Supplementary Dataset 3 [file srep16625-s4.zip › dataset3/0864.tif]

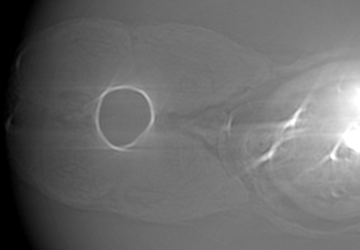

Supplement: Supplementary Dataset 3 [file srep16625-s4.zip › dataset3/0865.tif]

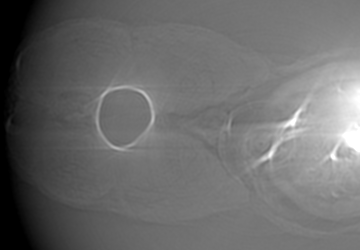

Supplement: Supplementary Dataset 3 [file srep16625-s4.zip › dataset3/0866.tif]

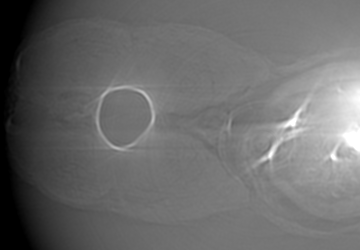

Supplement: Supplementary Dataset 3 [file srep16625-s4.zip › dataset3/0867.tif]

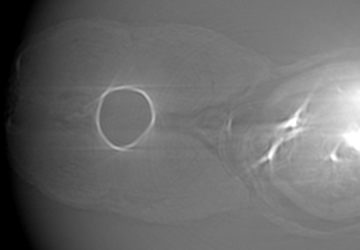

Supplement: Supplementary Dataset 3 [file srep16625-s4.zip › dataset3/0868.tif]

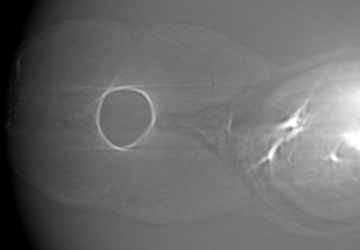

Supplement: Supplementary Dataset 3 [file srep16625-s4.zip › dataset3/0869.tif]

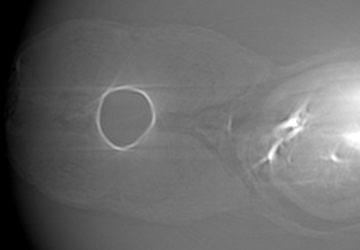

Supplement: Supplementary Dataset 3 [file srep16625-s4.zip › dataset3/0870.tif]

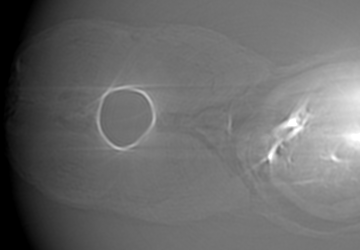

Supplement: Supplementary Dataset 3 [file srep16625-s4.zip › dataset3/0871.tif]

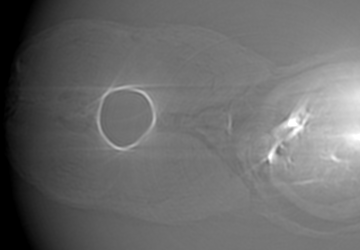

Supplement: Supplementary Dataset 3 [file srep16625-s4.zip › dataset3/0872.tif]

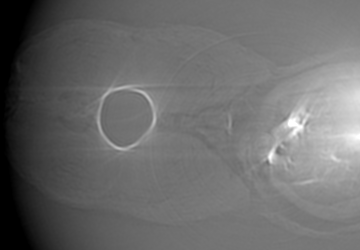

Supplement: Supplementary Dataset 3 [file srep16625-s4.zip › dataset3/0873.tif]
