# Supplementary figures and images for: X-ray phase-contrast tomography for high-spatial-resolution zebrafish muscle imaging (part 8 of 8)
Source: Sci Rep. 2015 Nov 13;5:16625. doi: 10.1038/srep16625 (PMC4643221; doi:10.1038/srep16625)

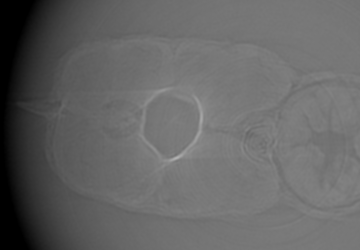

Supplement: Supplementary Dataset 4 [file srep16625-s5.zip › dataset4/1086.tif]

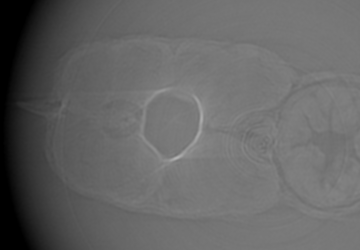

Supplement: Supplementary Dataset 4 [file srep16625-s5.zip › dataset4/1087.tif]

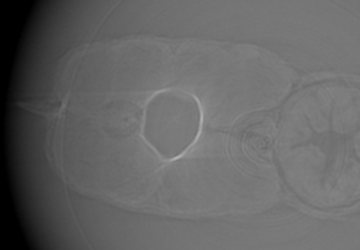

Supplement: Supplementary Dataset 4 [file srep16625-s5.zip › dataset4/1088.tif]

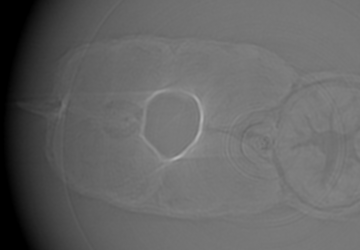

Supplement: Supplementary Dataset 4 [file srep16625-s5.zip › dataset4/1089.tif]

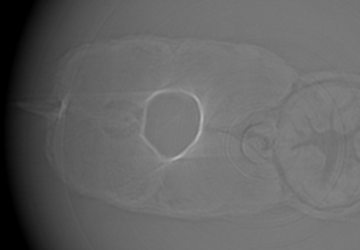

Supplement: Supplementary Dataset 4 [file srep16625-s5.zip › dataset4/1090.tif]

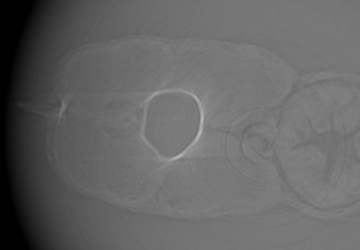

Supplement: Supplementary Dataset 4 [file srep16625-s5.zip › dataset4/1091.tif]

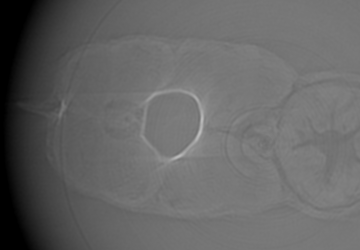

Supplement: Supplementary Dataset 4 [file srep16625-s5.zip › dataset4/1092.tif]

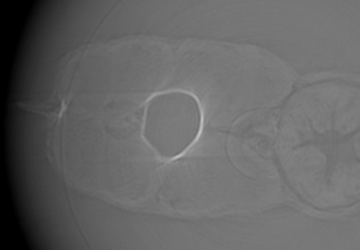

Supplement: Supplementary Dataset 4 [file srep16625-s5.zip › dataset4/1093.tif]

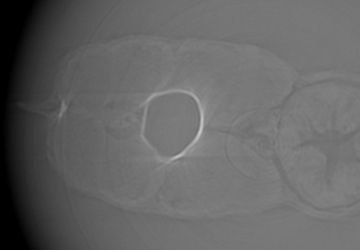

Supplement: Supplementary Dataset 4 [file srep16625-s5.zip › dataset4/1094.tif]

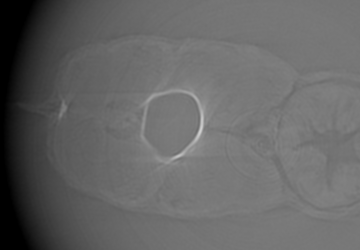

Supplement: Supplementary Dataset 4 [file srep16625-s5.zip › dataset4/1095.tif]

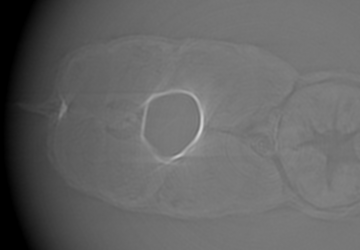

Supplement: Supplementary Dataset 4 [file srep16625-s5.zip › dataset4/1096.tif]

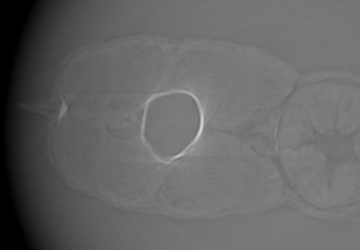

Supplement: Supplementary Dataset 4 [file srep16625-s5.zip › dataset4/1097.tif]

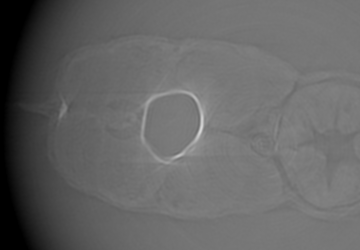

Supplement: Supplementary Dataset 4 [file srep16625-s5.zip › dataset4/1098.tif]

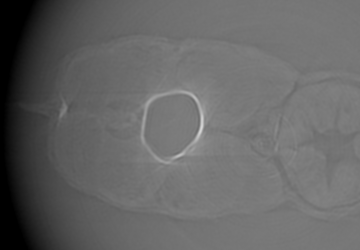

Supplement: Supplementary Dataset 4 [file srep16625-s5.zip › dataset4/1099.tif]

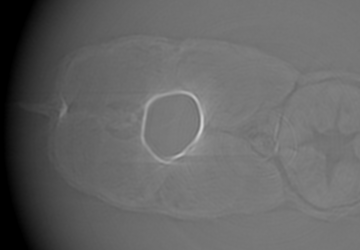

Supplement: Supplementary Dataset 4 [file srep16625-s5.zip › dataset4/1100.tif]

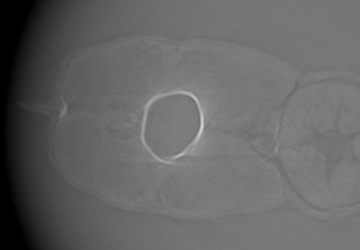

Supplement: Supplementary Dataset 4 [file srep16625-s5.zip › dataset4/1101.tif]

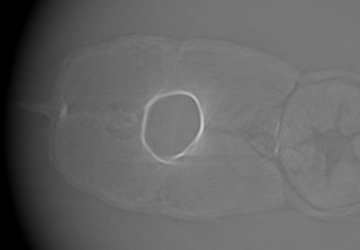

Supplement: Supplementary Dataset 4 [file srep16625-s5.zip › dataset4/1102.tif]

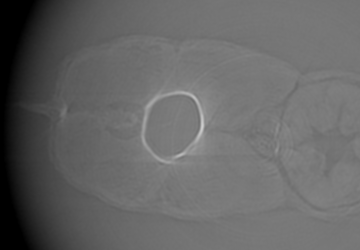

Supplement: Supplementary Dataset 4 [file srep16625-s5.zip › dataset4/1103.tif]

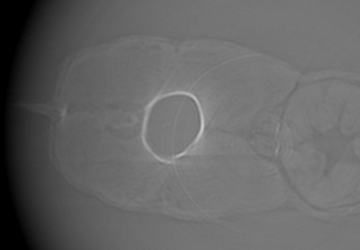

Supplement: Supplementary Dataset 4 [file srep16625-s5.zip › dataset4/1104.tif]

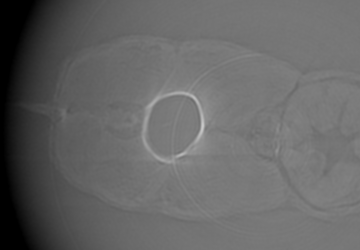

Supplement: Supplementary Dataset 4 [file srep16625-s5.zip › dataset4/1105.tif]

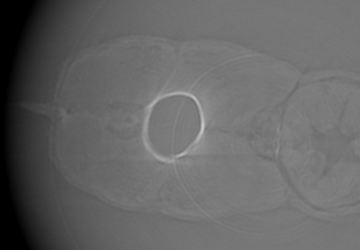

Supplement: Supplementary Dataset 4 [file srep16625-s5.zip › dataset4/1106.tif]

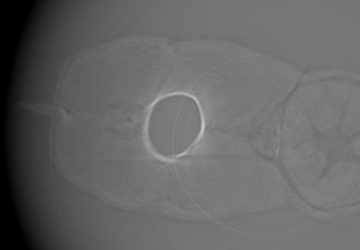

Supplement: Supplementary Dataset 4 [file srep16625-s5.zip › dataset4/1107.tif]

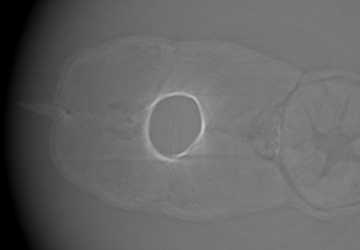

Supplement: Supplementary Dataset 4 [file srep16625-s5.zip › dataset4/1108.tif]

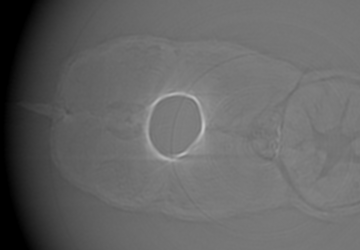

Supplement: Supplementary Dataset 4 [file srep16625-s5.zip › dataset4/1109.tif]

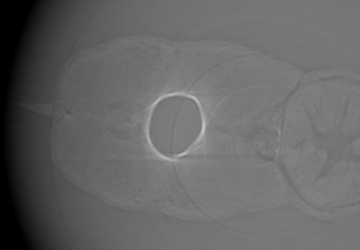

Supplement: Supplementary Dataset 4 [file srep16625-s5.zip › dataset4/1110.tif]

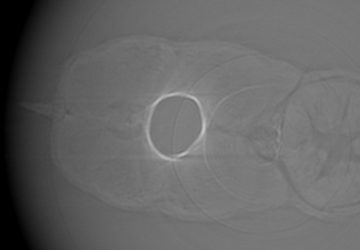

Supplement: Supplementary Dataset 4 [file srep16625-s5.zip › dataset4/1111.tif]

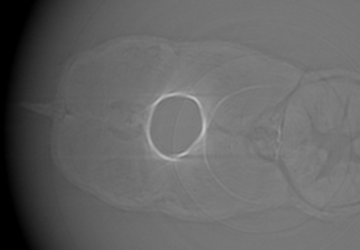

Supplement: Supplementary Dataset 4 [file srep16625-s5.zip › dataset4/1112.tif]

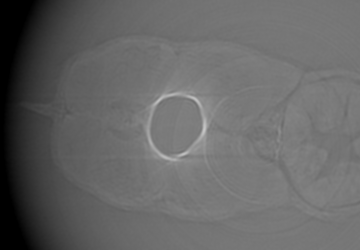

Supplement: Supplementary Dataset 4 [file srep16625-s5.zip › dataset4/1113.tif]

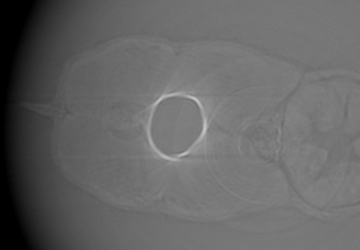

Supplement: Supplementary Dataset 4 [file srep16625-s5.zip › dataset4/1114.tif]

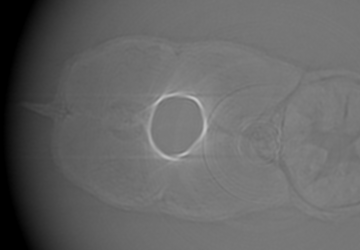

Supplement: Supplementary Dataset 4 [file srep16625-s5.zip › dataset4/1115.tif]

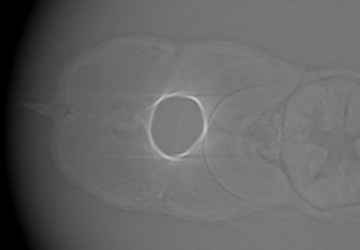

Supplement: Supplementary Dataset 4 [file srep16625-s5.zip › dataset4/1116.tif]

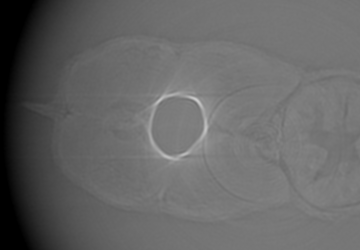

Supplement: Supplementary Dataset 4 [file srep16625-s5.zip › dataset4/1117.tif]

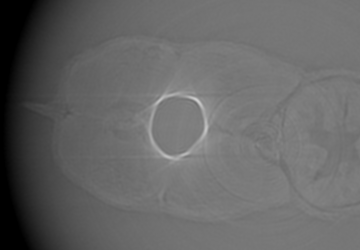

Supplement: Supplementary Dataset 4 [file srep16625-s5.zip › dataset4/1118.tif]

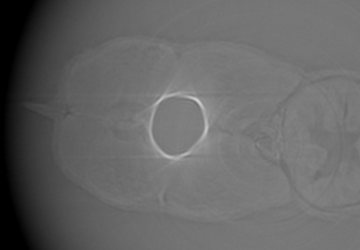

Supplement: Supplementary Dataset 4 [file srep16625-s5.zip › dataset4/1119.tif]

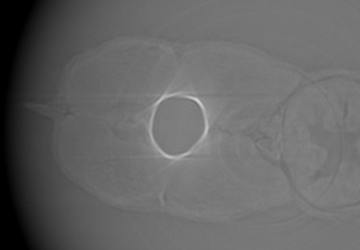

Supplement: Supplementary Dataset 4 [file srep16625-s5.zip › dataset4/1120.tif]

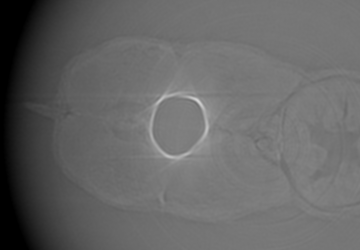

Supplement: Supplementary Dataset 4 [file srep16625-s5.zip › dataset4/1121.tif]

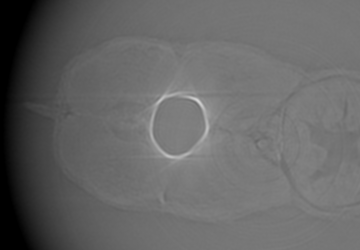

Supplement: Supplementary Dataset 4 [file srep16625-s5.zip › dataset4/1122.tif]

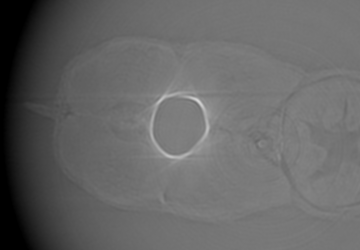

Supplement: Supplementary Dataset 4 [file srep16625-s5.zip › dataset4/1123.tif]

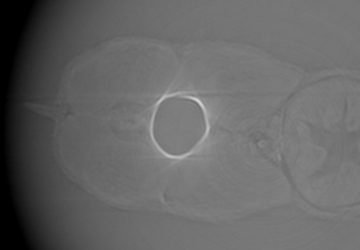

Supplement: Supplementary Dataset 4 [file srep16625-s5.zip › dataset4/1124.tif]

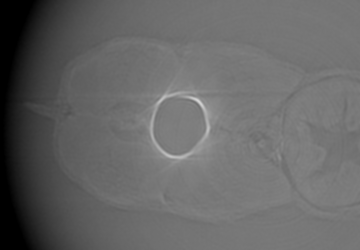

Supplement: Supplementary Dataset 4 [file srep16625-s5.zip › dataset4/1125.tif]

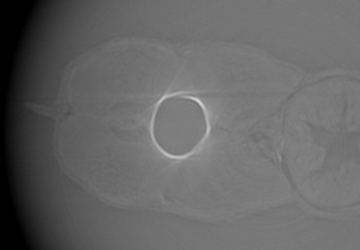

Supplement: Supplementary Dataset 4 [file srep16625-s5.zip › dataset4/1126.tif]

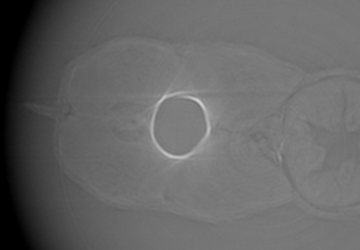

Supplement: Supplementary Dataset 4 [file srep16625-s5.zip › dataset4/1127.tif]

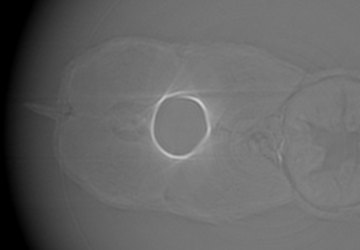

Supplement: Supplementary Dataset 4 [file srep16625-s5.zip › dataset4/1128.tif]

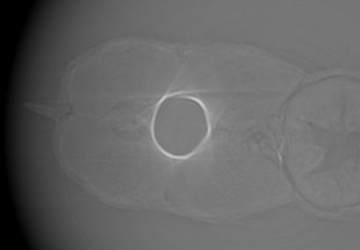

Supplement: Supplementary Dataset 4 [file srep16625-s5.zip › dataset4/1129.tif]

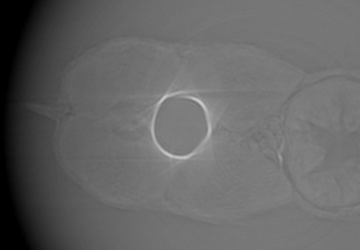

Supplement: Supplementary Dataset 4 [file srep16625-s5.zip › dataset4/1130.tif]

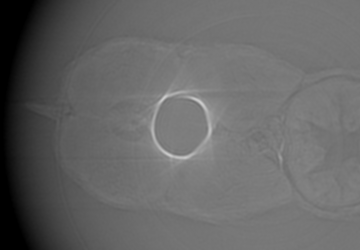

Supplement: Supplementary Dataset 4 [file srep16625-s5.zip › dataset4/1131.tif]

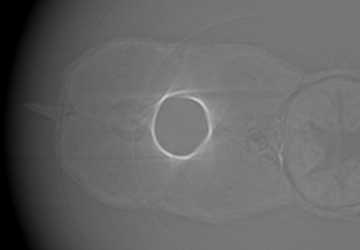

Supplement: Supplementary Dataset 4 [file srep16625-s5.zip › dataset4/1132.tif]

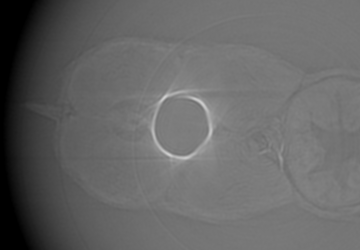

Supplement: Supplementary Dataset 4 [file srep16625-s5.zip › dataset4/1133.tif]

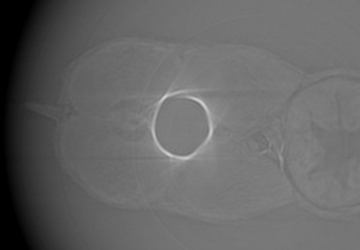

Supplement: Supplementary Dataset 4 [file srep16625-s5.zip › dataset4/1134.tif]

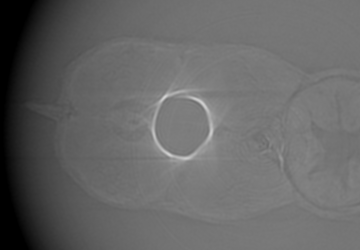

Supplement: Supplementary Dataset 4 [file srep16625-s5.zip › dataset4/1135.tif]

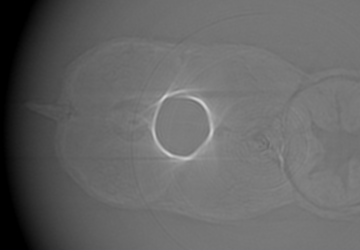

Supplement: Supplementary Dataset 4 [file srep16625-s5.zip › dataset4/1136.tif]

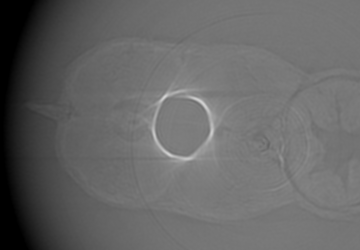

Supplement: Supplementary Dataset 4 [file srep16625-s5.zip › dataset4/1137.tif]

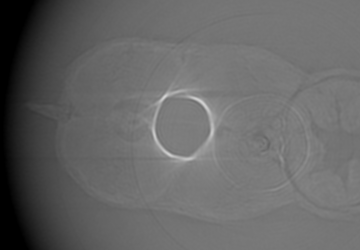

Supplement: Supplementary Dataset 4 [file srep16625-s5.zip › dataset4/1138.tif]

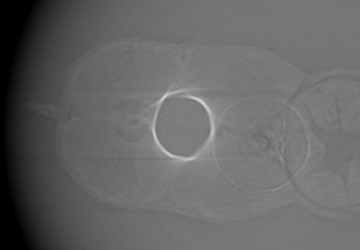

Supplement: Supplementary Dataset 4 [file srep16625-s5.zip › dataset4/1139.tif]

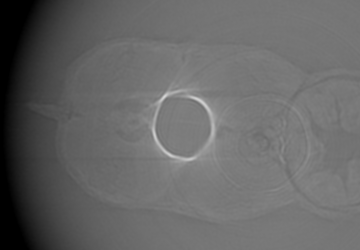

Supplement: Supplementary Dataset 4 [file srep16625-s5.zip › dataset4/1140.tif]

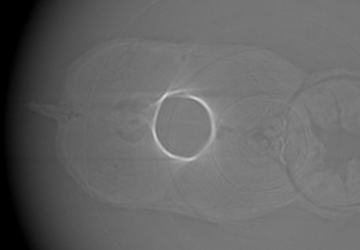

Supplement: Supplementary Dataset 4 [file srep16625-s5.zip › dataset4/1141.tif]

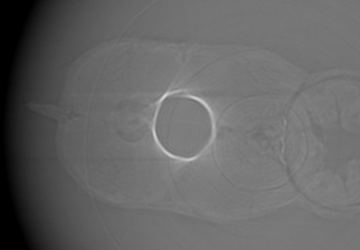

Supplement: Supplementary Dataset 4 [file srep16625-s5.zip › dataset4/1142.tif]

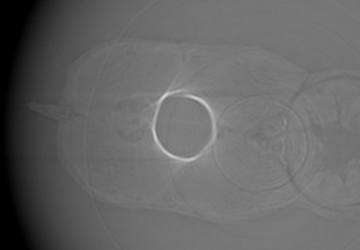

Supplement: Supplementary Dataset 4 [file srep16625-s5.zip › dataset4/1143.tif]

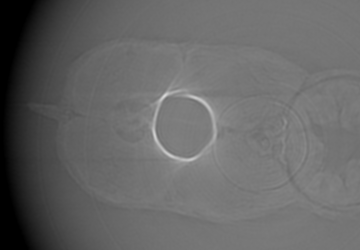

Supplement: Supplementary Dataset 4 [file srep16625-s5.zip › dataset4/1144.tif]

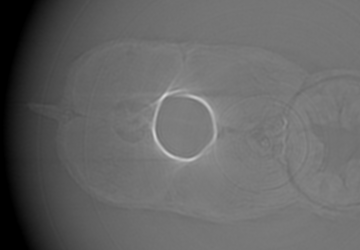

Supplement: Supplementary Dataset 4 [file srep16625-s5.zip › dataset4/1145.tif]

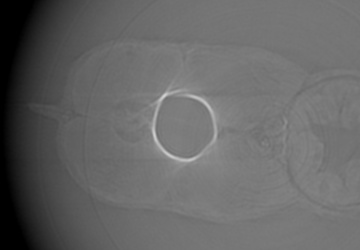

Supplement: Supplementary Dataset 4 [file srep16625-s5.zip › dataset4/1146.tif]

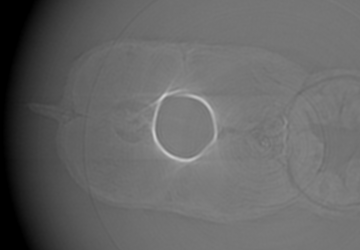

Supplement: Supplementary Dataset 4 [file srep16625-s5.zip › dataset4/1147.tif]

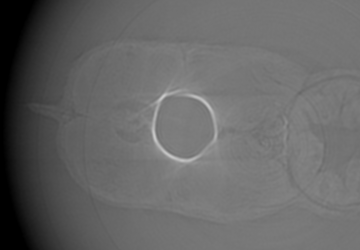

Supplement: Supplementary Dataset 4 [file srep16625-s5.zip › dataset4/1148.tif]

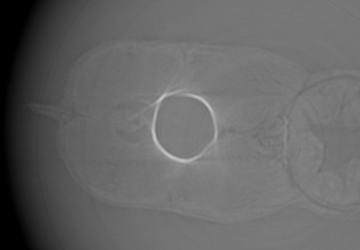

Supplement: Supplementary Dataset 4 [file srep16625-s5.zip › dataset4/1149.tif]

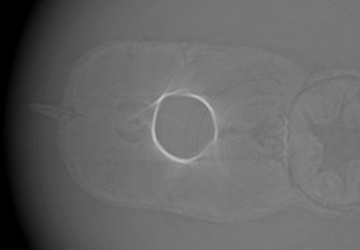

Supplement: Supplementary Dataset 4 [file srep16625-s5.zip › dataset4/1150.tif]

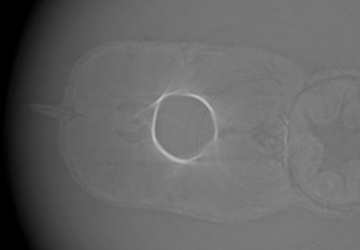

Supplement: Supplementary Dataset 4 [file srep16625-s5.zip › dataset4/1151.tif]

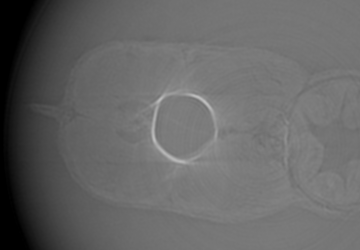

Supplement: Supplementary Dataset 4 [file srep16625-s5.zip › dataset4/1152.tif]

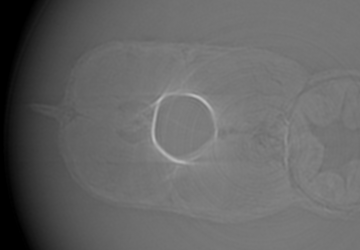

Supplement: Supplementary Dataset 4 [file srep16625-s5.zip › dataset4/1153.tif]

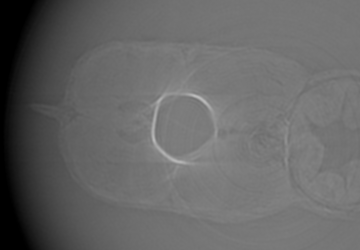

Supplement: Supplementary Dataset 4 [file srep16625-s5.zip › dataset4/1154.tif]

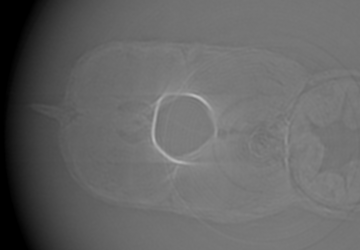

Supplement: Supplementary Dataset 4 [file srep16625-s5.zip › dataset4/1155.tif]

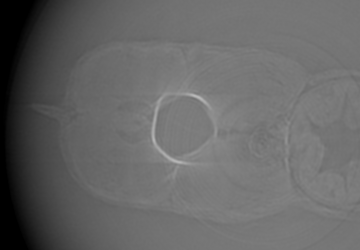

Supplement: Supplementary Dataset 4 [file srep16625-s5.zip › dataset4/1156.tif]

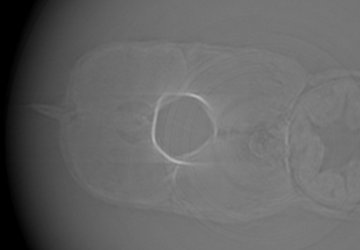

Supplement: Supplementary Dataset 4 [file srep16625-s5.zip › dataset4/1157.tif]

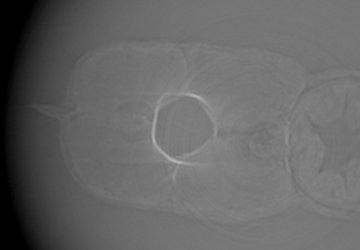

Supplement: Supplementary Dataset 4 [file srep16625-s5.zip › dataset4/1158.tif]

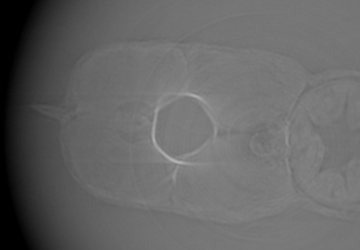

Supplement: Supplementary Dataset 4 [file srep16625-s5.zip › dataset4/1159.tif]

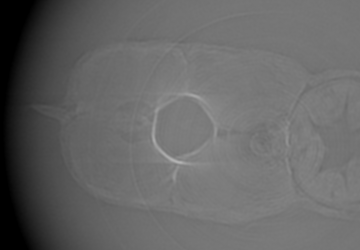

Supplement: Supplementary Dataset 4 [file srep16625-s5.zip › dataset4/1160.tif]

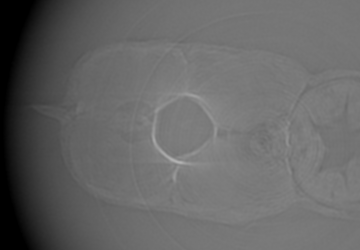

Supplement: Supplementary Dataset 4 [file srep16625-s5.zip › dataset4/1161.tif]

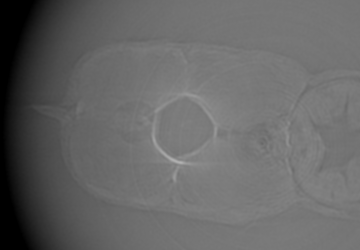

Supplement: Supplementary Dataset 4 [file srep16625-s5.zip › dataset4/1162.tif]

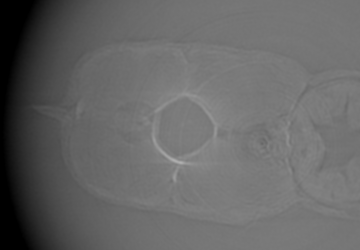

Supplement: Supplementary Dataset 4 [file srep16625-s5.zip › dataset4/1163.tif]

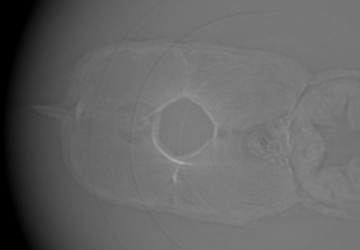

Supplement: Supplementary Dataset 4 [file srep16625-s5.zip › dataset4/1164.tif]

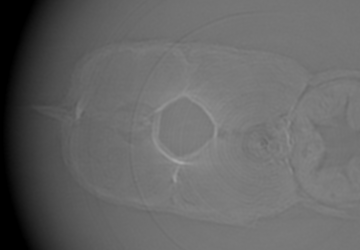

Supplement: Supplementary Dataset 4 [file srep16625-s5.zip › dataset4/1165.tif]

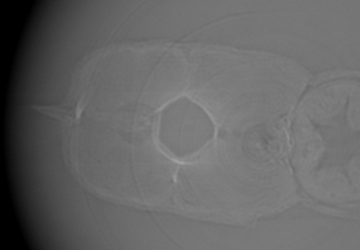

Supplement: Supplementary Dataset 4 [file srep16625-s5.zip › dataset4/1166.tif]

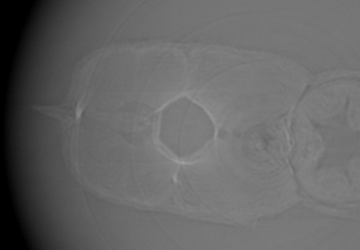

Supplement: Supplementary Dataset 4 [file srep16625-s5.zip › dataset4/1167.tif]

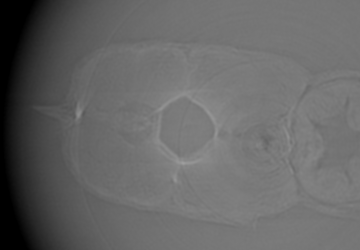

Supplement: Supplementary Dataset 4 [file srep16625-s5.zip › dataset4/1168.tif]

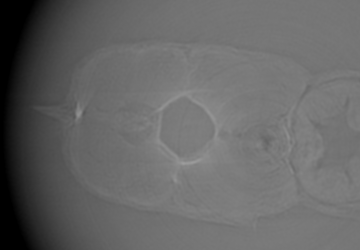

Supplement: Supplementary Dataset 4 [file srep16625-s5.zip › dataset4/1169.tif]

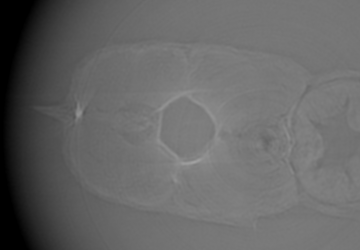

Supplement: Supplementary Dataset 4 [file srep16625-s5.zip › dataset4/1170.tif]

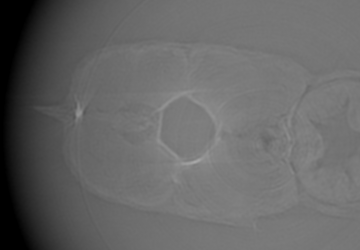

Supplement: Supplementary Dataset 4 [file srep16625-s5.zip › dataset4/1171.tif]

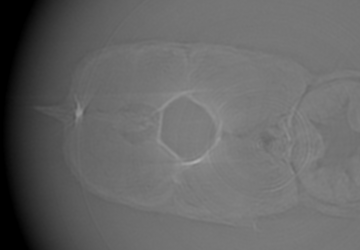

Supplement: Supplementary Dataset 4 [file srep16625-s5.zip › dataset4/1172.tif]

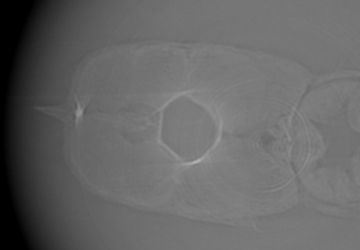

Supplement: Supplementary Dataset 4 [file srep16625-s5.zip › dataset4/1173.tif]

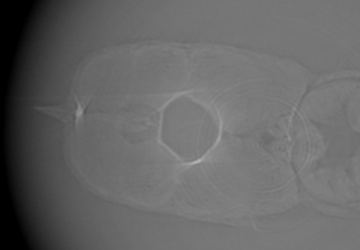

Supplement: Supplementary Dataset 4 [file srep16625-s5.zip › dataset4/1174.tif]

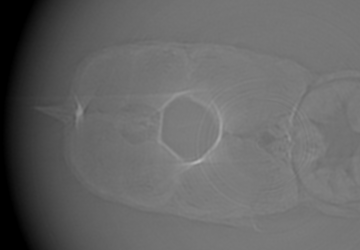

Supplement: Supplementary Dataset 4 [file srep16625-s5.zip › dataset4/1175.tif]

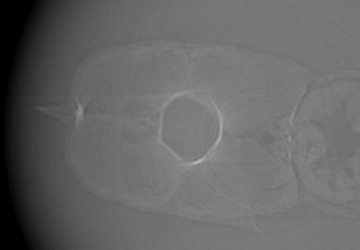

Supplement: Supplementary Dataset 4 [file srep16625-s5.zip › dataset4/1176.tif]

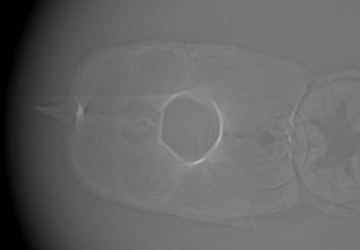

Supplement: Supplementary Dataset 4 [file srep16625-s5.zip › dataset4/1177.tif]

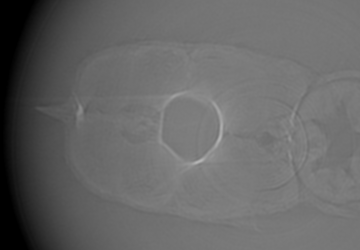

Supplement: Supplementary Dataset 4 [file srep16625-s5.zip › dataset4/1178.tif]

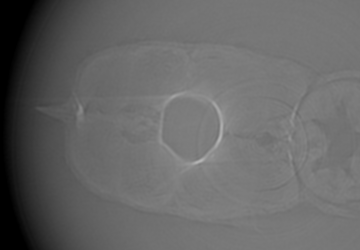

Supplement: Supplementary Dataset 4 [file srep16625-s5.zip › dataset4/1179.tif]

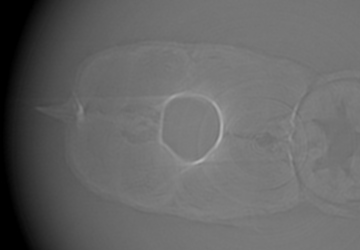

Supplement: Supplementary Dataset 4 [file srep16625-s5.zip › dataset4/1180.tif]

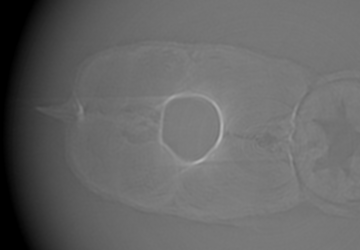

Supplement: Supplementary Dataset 4 [file srep16625-s5.zip › dataset4/1181.tif]

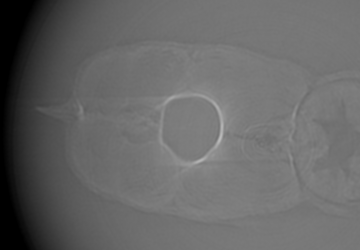

Supplement: Supplementary Dataset 4 [file srep16625-s5.zip › dataset4/1182.tif]

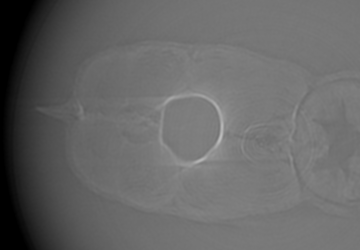

Supplement: Supplementary Dataset 4 [file srep16625-s5.zip › dataset4/1183.tif]

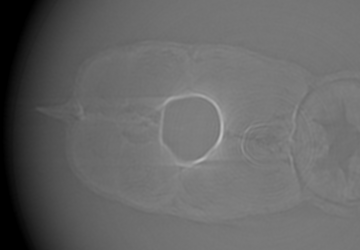

Supplement: Supplementary Dataset 4 [file srep16625-s5.zip › dataset4/1184.tif]

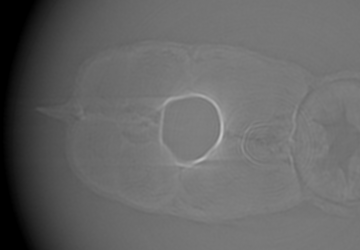

Supplement: Supplementary Dataset 4 [file srep16625-s5.zip › dataset4/1185.tif]
