# Supplementary figures and images for: X-ray phase-contrast tomography for high-spatial-resolution zebrafish muscle imaging (part 7 of 8)
Source: Sci Rep. 2015 Nov 13;5:16625. doi: 10.1038/srep16625 (PMC4643221; doi:10.1038/srep16625)

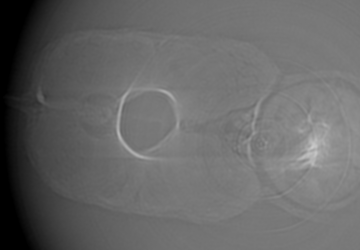

Supplement: Supplementary Dataset 4 [file srep16625-s5.zip › dataset4/0986.tif]

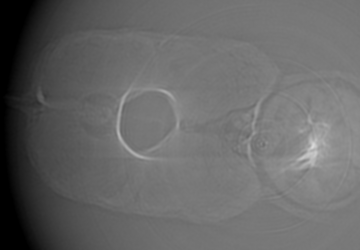

Supplement: Supplementary Dataset 4 [file srep16625-s5.zip › dataset4/0987.tif]

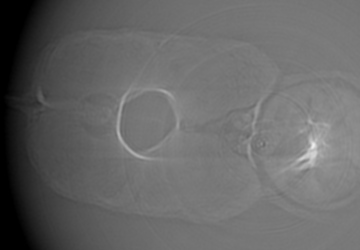

Supplement: Supplementary Dataset 4 [file srep16625-s5.zip › dataset4/0988.tif]

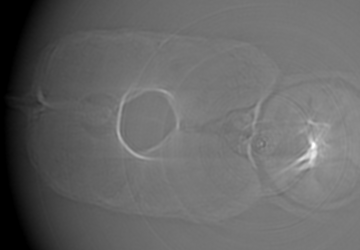

Supplement: Supplementary Dataset 4 [file srep16625-s5.zip › dataset4/0989.tif]

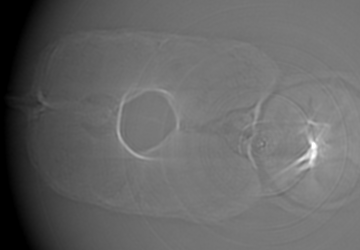

Supplement: Supplementary Dataset 4 [file srep16625-s5.zip › dataset4/0990.tif]

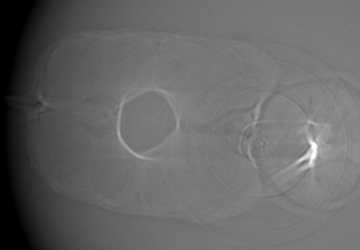

Supplement: Supplementary Dataset 4 [file srep16625-s5.zip › dataset4/0991.tif]

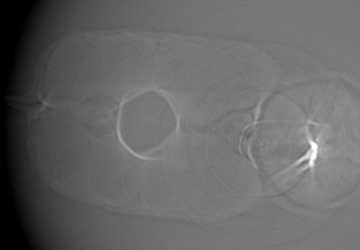

Supplement: Supplementary Dataset 4 [file srep16625-s5.zip › dataset4/0992.tif]

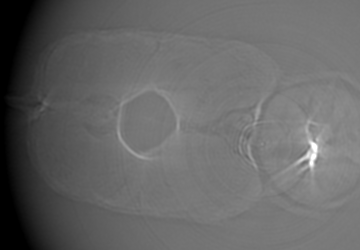

Supplement: Supplementary Dataset 4 [file srep16625-s5.zip › dataset4/0993.tif]

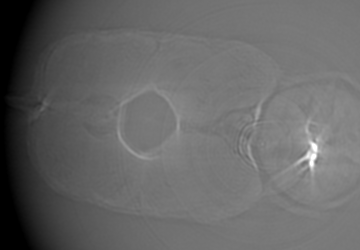

Supplement: Supplementary Dataset 4 [file srep16625-s5.zip › dataset4/0994.tif]

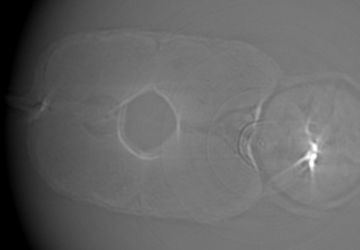

Supplement: Supplementary Dataset 4 [file srep16625-s5.zip › dataset4/0995.tif]

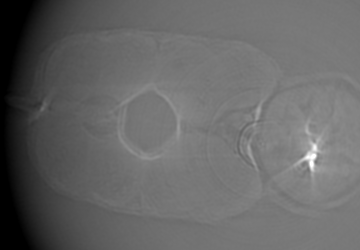

Supplement: Supplementary Dataset 4 [file srep16625-s5.zip › dataset4/0996.tif]

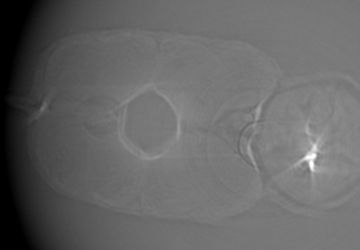

Supplement: Supplementary Dataset 4 [file srep16625-s5.zip › dataset4/0997.tif]

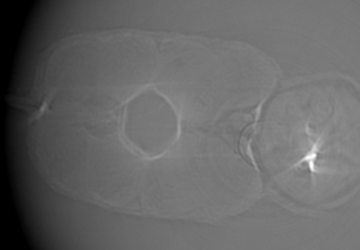

Supplement: Supplementary Dataset 4 [file srep16625-s5.zip › dataset4/0998.tif]

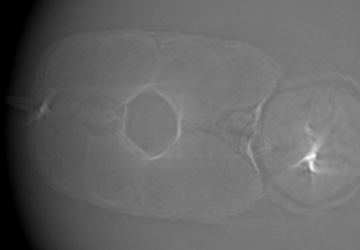

Supplement: Supplementary Dataset 4 [file srep16625-s5.zip › dataset4/0999.tif]

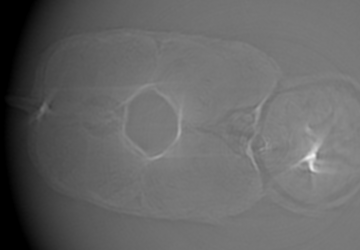

Supplement: Supplementary Dataset 4 [file srep16625-s5.zip › dataset4/1000.tif]

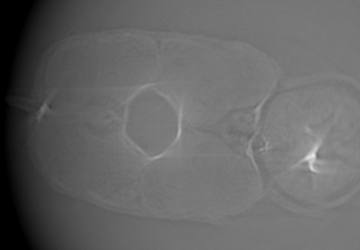

Supplement: Supplementary Dataset 4 [file srep16625-s5.zip › dataset4/1001.tif]

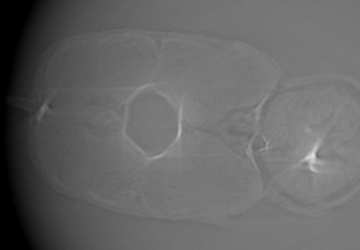

Supplement: Supplementary Dataset 4 [file srep16625-s5.zip › dataset4/1002.tif]

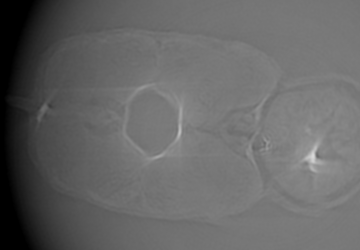

Supplement: Supplementary Dataset 4 [file srep16625-s5.zip › dataset4/1003.tif]

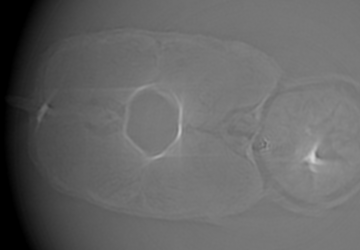

Supplement: Supplementary Dataset 4 [file srep16625-s5.zip › dataset4/1004.tif]

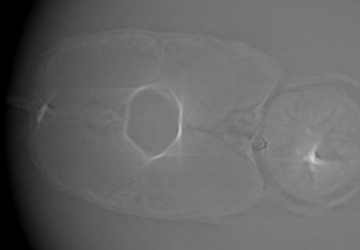

Supplement: Supplementary Dataset 4 [file srep16625-s5.zip › dataset4/1005.tif]

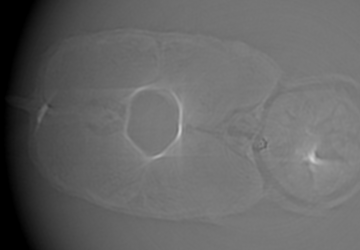

Supplement: Supplementary Dataset 4 [file srep16625-s5.zip › dataset4/1006.tif]

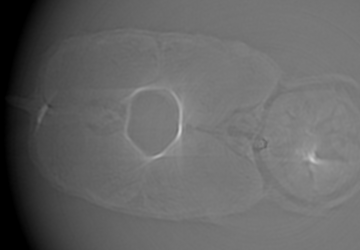

Supplement: Supplementary Dataset 4 [file srep16625-s5.zip › dataset4/1007.tif]

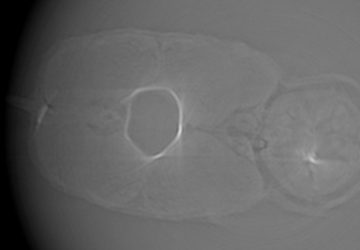

Supplement: Supplementary Dataset 4 [file srep16625-s5.zip › dataset4/1008.tif]

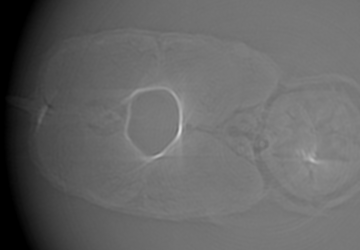

Supplement: Supplementary Dataset 4 [file srep16625-s5.zip › dataset4/1009.tif]

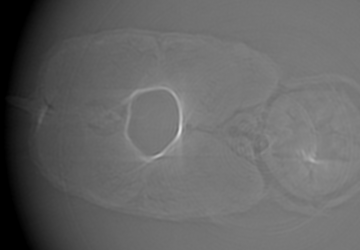

Supplement: Supplementary Dataset 4 [file srep16625-s5.zip › dataset4/1010.tif]

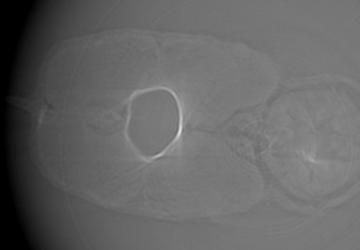

Supplement: Supplementary Dataset 4 [file srep16625-s5.zip › dataset4/1011.tif]

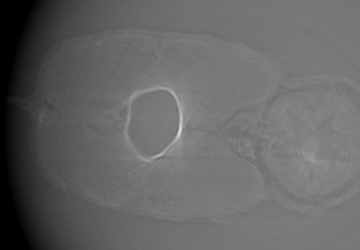

Supplement: Supplementary Dataset 4 [file srep16625-s5.zip › dataset4/1012.tif]

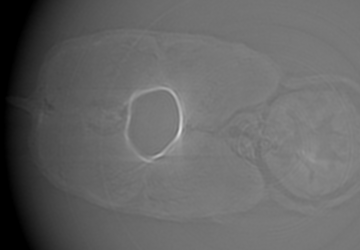

Supplement: Supplementary Dataset 4 [file srep16625-s5.zip › dataset4/1013.tif]

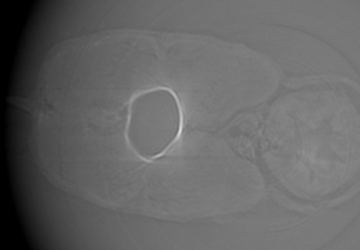

Supplement: Supplementary Dataset 4 [file srep16625-s5.zip › dataset4/1014.tif]

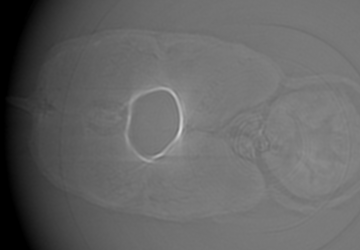

Supplement: Supplementary Dataset 4 [file srep16625-s5.zip › dataset4/1015.tif]

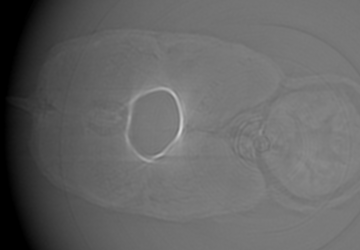

Supplement: Supplementary Dataset 4 [file srep16625-s5.zip › dataset4/1016.tif]

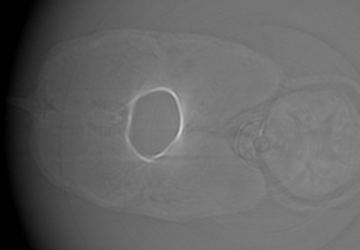

Supplement: Supplementary Dataset 4 [file srep16625-s5.zip › dataset4/1017.tif]

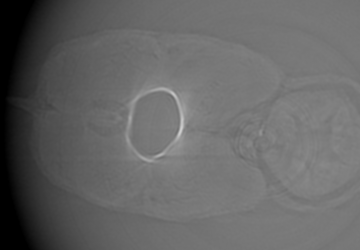

Supplement: Supplementary Dataset 4 [file srep16625-s5.zip › dataset4/1018.tif]

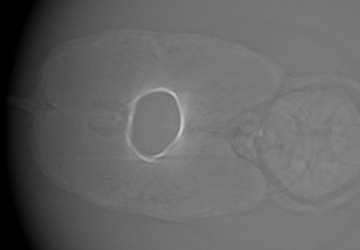

Supplement: Supplementary Dataset 4 [file srep16625-s5.zip › dataset4/1019.tif]

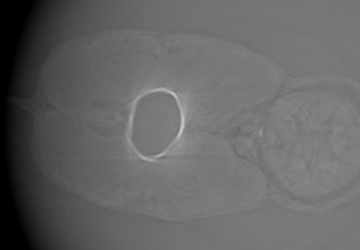

Supplement: Supplementary Dataset 4 [file srep16625-s5.zip › dataset4/1020.tif]

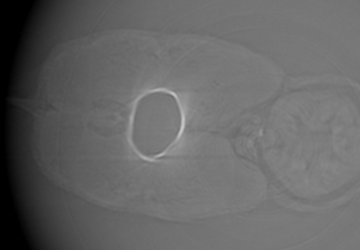

Supplement: Supplementary Dataset 4 [file srep16625-s5.zip › dataset4/1021.tif]

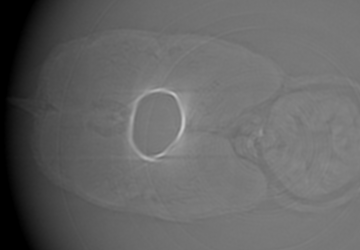

Supplement: Supplementary Dataset 4 [file srep16625-s5.zip › dataset4/1022.tif]

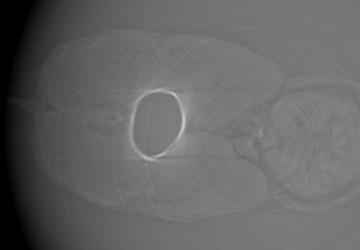

Supplement: Supplementary Dataset 4 [file srep16625-s5.zip › dataset4/1023.tif]

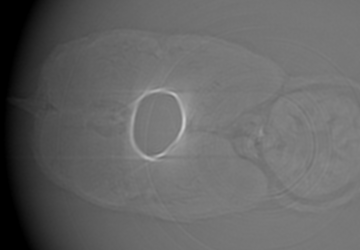

Supplement: Supplementary Dataset 4 [file srep16625-s5.zip › dataset4/1024.tif]

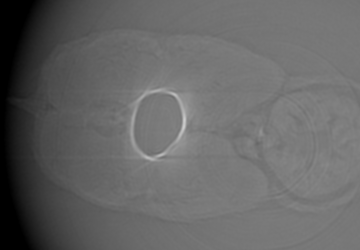

Supplement: Supplementary Dataset 4 [file srep16625-s5.zip › dataset4/1025.tif]

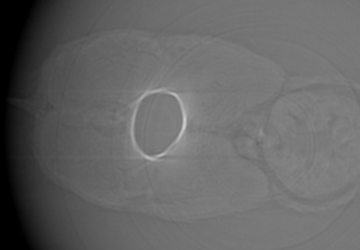

Supplement: Supplementary Dataset 4 [file srep16625-s5.zip › dataset4/1026.tif]

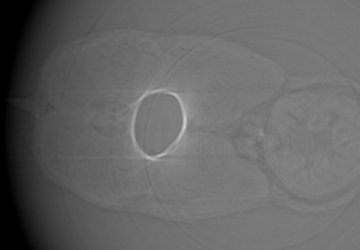

Supplement: Supplementary Dataset 4 [file srep16625-s5.zip › dataset4/1027.tif]

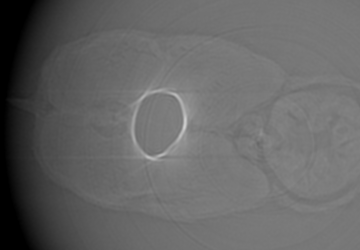

Supplement: Supplementary Dataset 4 [file srep16625-s5.zip › dataset4/1028.tif]

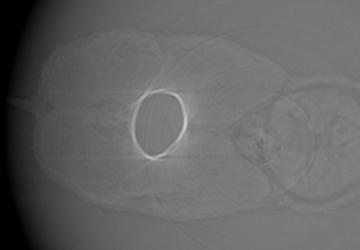

Supplement: Supplementary Dataset 4 [file srep16625-s5.zip › dataset4/1029.tif]

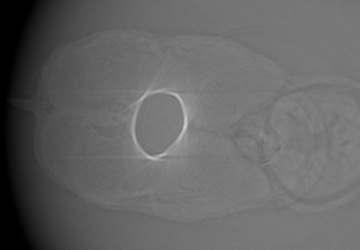

Supplement: Supplementary Dataset 4 [file srep16625-s5.zip › dataset4/1030.tif]

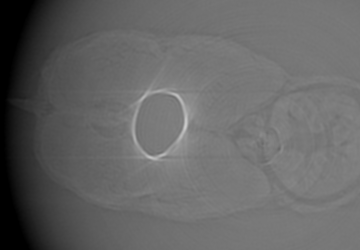

Supplement: Supplementary Dataset 4 [file srep16625-s5.zip › dataset4/1031.tif]

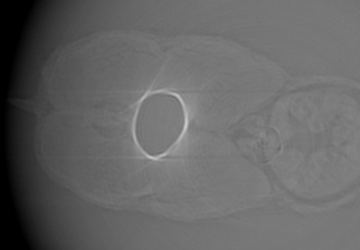

Supplement: Supplementary Dataset 4 [file srep16625-s5.zip › dataset4/1032.tif]

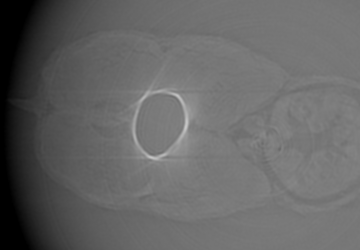

Supplement: Supplementary Dataset 4 [file srep16625-s5.zip › dataset4/1033.tif]

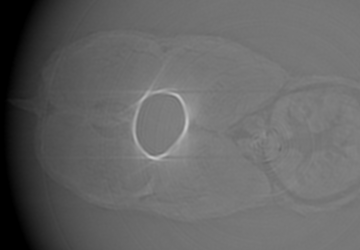

Supplement: Supplementary Dataset 4 [file srep16625-s5.zip › dataset4/1034.tif]

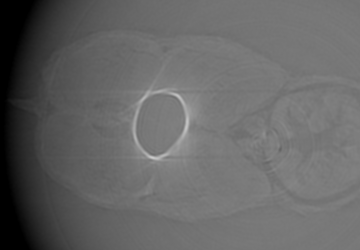

Supplement: Supplementary Dataset 4 [file srep16625-s5.zip › dataset4/1035.tif]

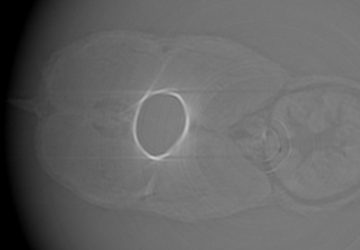

Supplement: Supplementary Dataset 4 [file srep16625-s5.zip › dataset4/1036.tif]

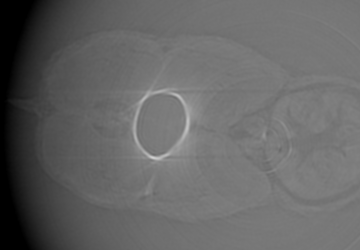

Supplement: Supplementary Dataset 4 [file srep16625-s5.zip › dataset4/1037.tif]

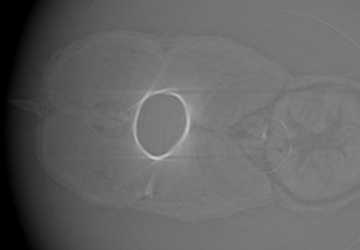

Supplement: Supplementary Dataset 4 [file srep16625-s5.zip › dataset4/1038.tif]

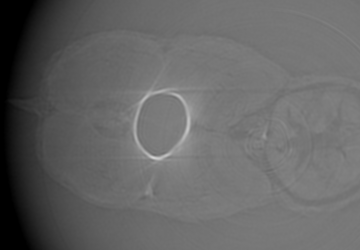

Supplement: Supplementary Dataset 4 [file srep16625-s5.zip › dataset4/1039.tif]

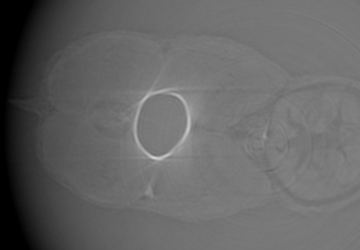

Supplement: Supplementary Dataset 4 [file srep16625-s5.zip › dataset4/1040.tif]

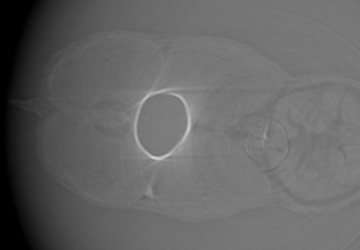

Supplement: Supplementary Dataset 4 [file srep16625-s5.zip › dataset4/1041.tif]

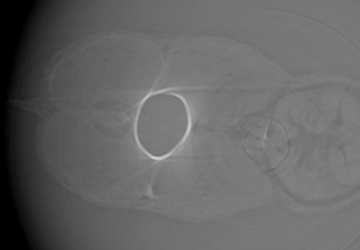

Supplement: Supplementary Dataset 4 [file srep16625-s5.zip › dataset4/1042.tif]

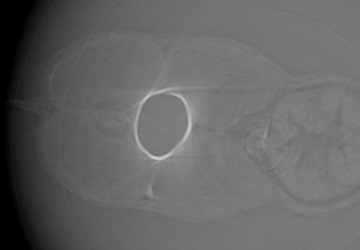

Supplement: Supplementary Dataset 4 [file srep16625-s5.zip › dataset4/1043.tif]

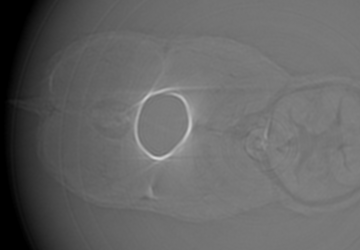

Supplement: Supplementary Dataset 4 [file srep16625-s5.zip › dataset4/1044.tif]

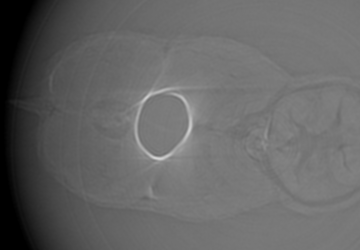

Supplement: Supplementary Dataset 4 [file srep16625-s5.zip › dataset4/1045.tif]

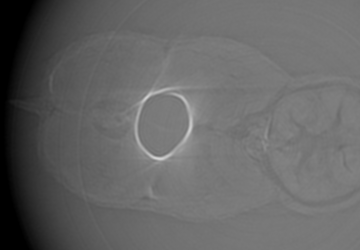

Supplement: Supplementary Dataset 4 [file srep16625-s5.zip › dataset4/1046.tif]

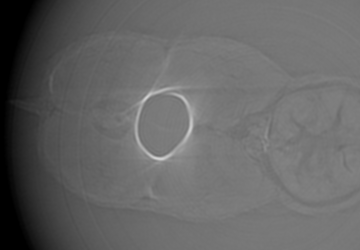

Supplement: Supplementary Dataset 4 [file srep16625-s5.zip › dataset4/1047.tif]

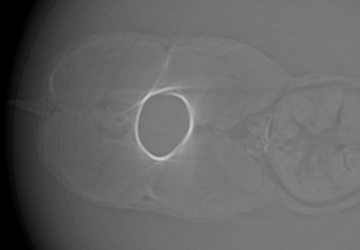

Supplement: Supplementary Dataset 4 [file srep16625-s5.zip › dataset4/1048.tif]

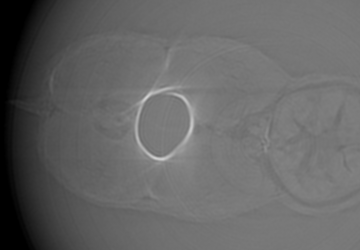

Supplement: Supplementary Dataset 4 [file srep16625-s5.zip › dataset4/1049.tif]

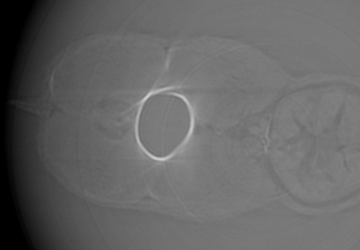

Supplement: Supplementary Dataset 4 [file srep16625-s5.zip › dataset4/1050.tif]

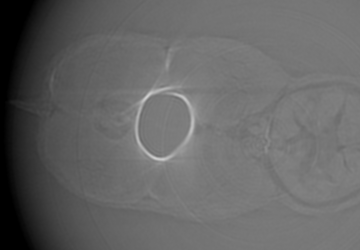

Supplement: Supplementary Dataset 4 [file srep16625-s5.zip › dataset4/1051.tif]

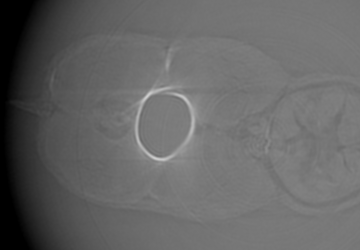

Supplement: Supplementary Dataset 4 [file srep16625-s5.zip › dataset4/1052.tif]

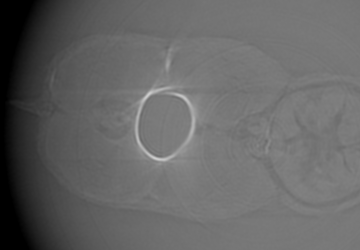

Supplement: Supplementary Dataset 4 [file srep16625-s5.zip › dataset4/1053.tif]

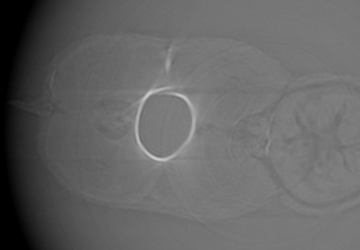

Supplement: Supplementary Dataset 4 [file srep16625-s5.zip › dataset4/1054.tif]

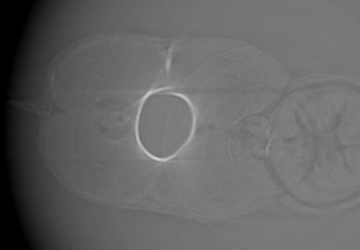

Supplement: Supplementary Dataset 4 [file srep16625-s5.zip › dataset4/1055.tif]

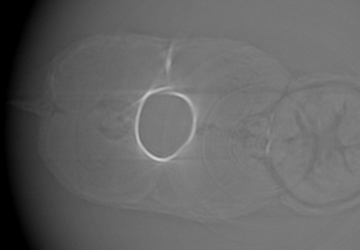

Supplement: Supplementary Dataset 4 [file srep16625-s5.zip › dataset4/1056.tif]

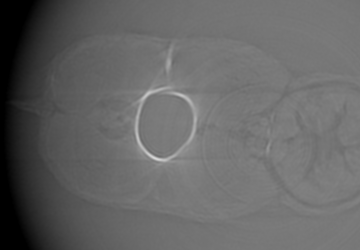

Supplement: Supplementary Dataset 4 [file srep16625-s5.zip › dataset4/1057.tif]

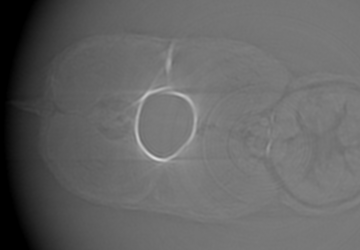

Supplement: Supplementary Dataset 4 [file srep16625-s5.zip › dataset4/1058.tif]

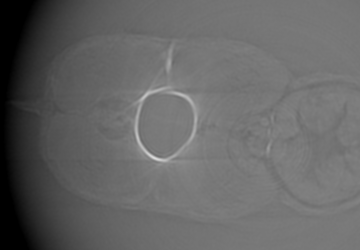

Supplement: Supplementary Dataset 4 [file srep16625-s5.zip › dataset4/1059.tif]

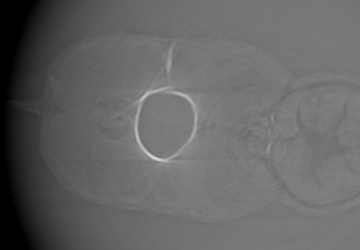

Supplement: Supplementary Dataset 4 [file srep16625-s5.zip › dataset4/1060.tif]

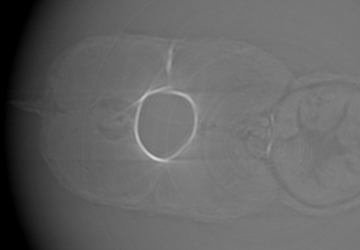

Supplement: Supplementary Dataset 4 [file srep16625-s5.zip › dataset4/1061.tif]

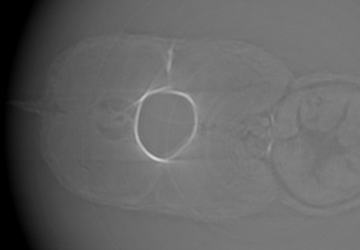

Supplement: Supplementary Dataset 4 [file srep16625-s5.zip › dataset4/1062.tif]

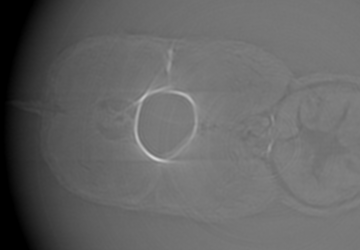

Supplement: Supplementary Dataset 4 [file srep16625-s5.zip › dataset4/1063.tif]

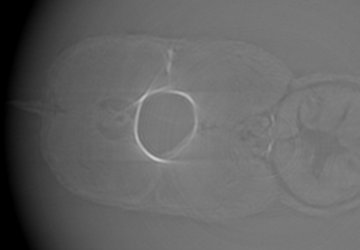

Supplement: Supplementary Dataset 4 [file srep16625-s5.zip › dataset4/1064.tif]

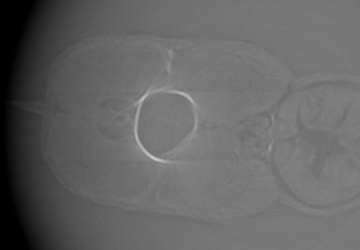

Supplement: Supplementary Dataset 4 [file srep16625-s5.zip › dataset4/1065.tif]

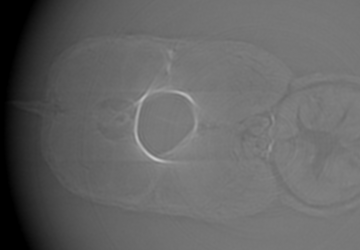

Supplement: Supplementary Dataset 4 [file srep16625-s5.zip › dataset4/1066.tif]

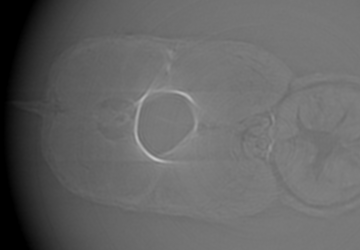

Supplement: Supplementary Dataset 4 [file srep16625-s5.zip › dataset4/1067.tif]

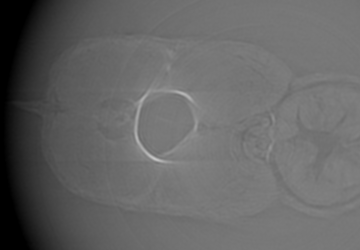

Supplement: Supplementary Dataset 4 [file srep16625-s5.zip › dataset4/1068.tif]

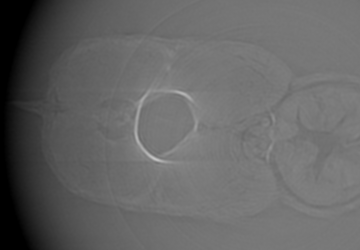

Supplement: Supplementary Dataset 4 [file srep16625-s5.zip › dataset4/1069.tif]

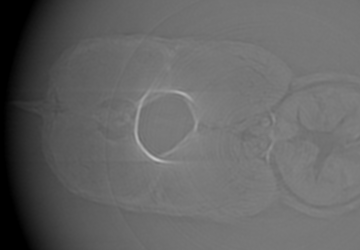

Supplement: Supplementary Dataset 4 [file srep16625-s5.zip › dataset4/1070.tif]

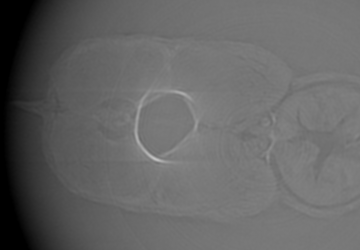

Supplement: Supplementary Dataset 4 [file srep16625-s5.zip › dataset4/1071.tif]

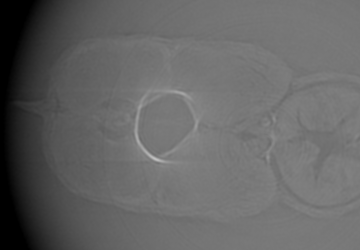

Supplement: Supplementary Dataset 4 [file srep16625-s5.zip › dataset4/1072.tif]

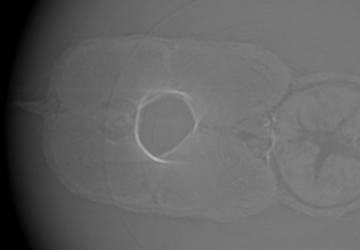

Supplement: Supplementary Dataset 4 [file srep16625-s5.zip › dataset4/1073.tif]

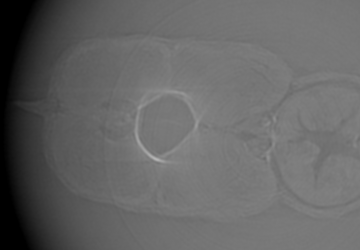

Supplement: Supplementary Dataset 4 [file srep16625-s5.zip › dataset4/1074.tif]

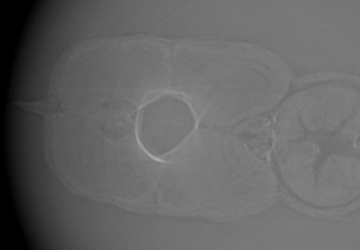

Supplement: Supplementary Dataset 4 [file srep16625-s5.zip › dataset4/1075.tif]

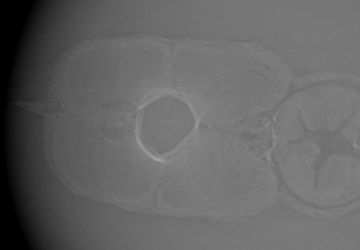

Supplement: Supplementary Dataset 4 [file srep16625-s5.zip › dataset4/1076.tif]

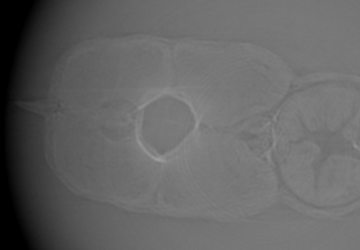

Supplement: Supplementary Dataset 4 [file srep16625-s5.zip › dataset4/1077.tif]

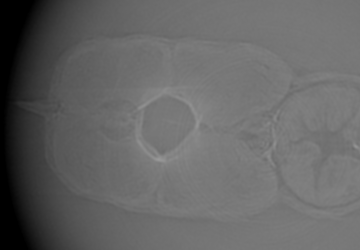

Supplement: Supplementary Dataset 4 [file srep16625-s5.zip › dataset4/1078.tif]

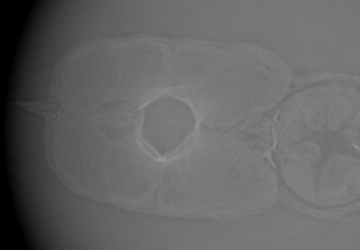

Supplement: Supplementary Dataset 4 [file srep16625-s5.zip › dataset4/1079.tif]

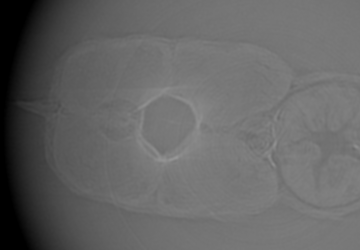

Supplement: Supplementary Dataset 4 [file srep16625-s5.zip › dataset4/1080.tif]

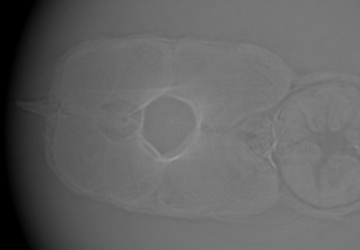

Supplement: Supplementary Dataset 4 [file srep16625-s5.zip › dataset4/1081.tif]

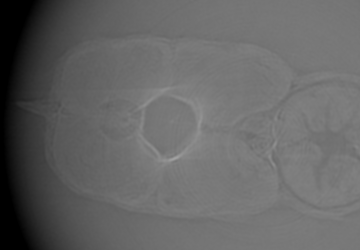

Supplement: Supplementary Dataset 4 [file srep16625-s5.zip › dataset4/1082.tif]

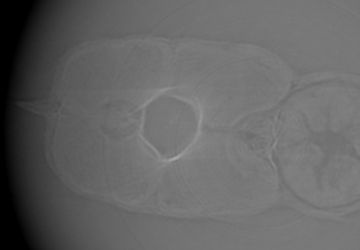

Supplement: Supplementary Dataset 4 [file srep16625-s5.zip › dataset4/1083.tif]

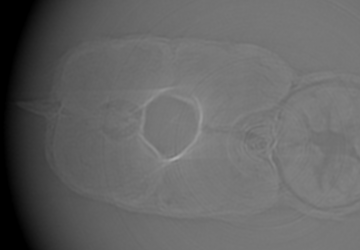

Supplement: Supplementary Dataset 4 [file srep16625-s5.zip › dataset4/1084.tif]

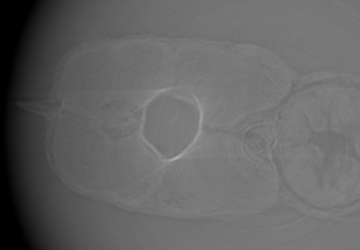

Supplement: Supplementary Dataset 4 [file srep16625-s5.zip › dataset4/1085.tif]
